# Canonical Antibodies Adopt Distinct Binding Modes to Recognize Viral Glycan Shields

**DOI:** 10.1002/advs.77172

**Published:** 2026-08-14

**Authors:** Jiaxuan Cheng, Evan M. Cale, Amirabbas Maghsoudi, Matthew S. Sutton, Nancy S. Longo, Rebecca A. Gillespie, Lingshu Wang, Ivan Kosik, Sue Chong, Atsuhiro Yasuhara, Tatsiana Bylund, Arne Schön, Prabhanshu Tripathi, Yaroslav Tsybovsky, Haotian Lei, Nicholas C. Morano, Allison B. Lupatkin, Abraham J. Morton, Zabrina C. Lang, Jordan E. Becker, Isabella R. Frascilla, Myungjin Lee, Ning Li, Cuiping Liu, Ryan S. Roark, Chen‐Hsiang Shen, I‐Ting Teng, David J. Van Wazer, Danyi Wang, Lingyuan Wu, Goran Ahlsen, Mike Castro, Haijuan Du, Michael J. Ernandes, Tiansheng Li, Bob C. Lin, Mark K. Louder, Krisha McKee, Jonah S. Merriam, Sijy O'Dell, Li Ou, Sergei Pletnev, David Prikryl, Qi Qiu, Sarah Rubin, Mallika Sastry, Stephen D. Schmidt, Asif Shajahan, Andrea R. Shiakolas, Sanjay Srivatsan, Baoshan Zhang, Qiong Zhou, Mark Connors, Jason G. Gall, Yicheng Guo, Rick K. Huang, Yaoxing Huang, Richard A. Koup, Q. Paula Lei, John R. Mascola, Reda Rawi, Leonid Serebryannyy, Lawrence Shapiro, Zizhang Sheng, David D. Ho, Patrick C. Wilson, Jonathan W. Yewdell, Theodore C. Pierson, Masaru Kanekiyo, Nicole A. Doria‐Rose, Peter D. Kwong, Tongqing Zhou

**Affiliations:** ^1^ Vaccine Research Center National Institute of Allergy and Infectious Diseases National Institutes of Health Bethesda Maryland USA; ^2^ Cellular Biology Section Laboratory of Viral Diseases National Institute of Allergy and Infectious Diseases Bethesda Maryland USA; ^3^ Department of Biochemistry and Molecular Biophysics and Zuckerman Mind Brain Behavior Institute Columbia University New York New York USA; ^4^ Aaron Diamond AIDS Research Center Columbia University Vagelos College of Physicians and Surgeons New York New York USA; ^5^ Drukier Institute for Children's Health Department of Pediatrics Weill Cornell Medicine New York New York USA; ^6^ Department of Biology Johns Hopkins University Baltimore Maryland USA; ^7^ Vaccine Research Center Electron Microscopy Unit Cancer Research Technology Program Frederick National Laboratory For Cancer Research Sponsored By the National Cancer Institute Frederick Maryland USA; ^8^ Research Technologies Branch National Institute of Allergy and Infectious Diseases National Institutes of Health Bethesda Maryland USA; ^9^ Laboratory of Cell Biology National Cancer Institute National Institutes of Health Bethesda Maryland USA; ^10^ Laboratory of Immunoregulation National Institute of Allergy and Infectious Diseases National Institutes of Health Bethesda Maryland USA

**Keywords:** broadly neutralizing antibody, cryo‐EM, glycan recognition, HIV‐1 envelope, homotypic assembly, multivalent binding, somatic hypermutation, viral glycan shield

## Abstract

Viral entry glycoproteins are often shielded from immune recognition by dense *N*‐linked glycans that limit antibody access to protein epitopes. While glycan‐reactive antibodies with unusual architectures have been described, how canonical Y‐shaped antibodies engage these glycan‐rich surfaces remains poorly defined. Here, we characterize two human antibodies, VRC35 and VRC36, isolated from an HIV‐1‐infected donor, that recognize diverse glycosylated viral glycoproteins. Cryo‐electron microscopy structural analyses of these antibodies in complex with viral entry glycoproteins, including HIV‐1 envelope, influenza hemagglutinin, SARS‐CoV‐2 spike, and the Lassa virus glycoprotein complex, reveal adaptive Fab stoichiometries ranging from single‐Fab binding to dimeric and higher‐order assemblies are mediated by intra‐ and inter‐IgG interactions that depend on local glycan organization. Dense glycan clustering on HIV‐1 and influenza glycoproteins supports multivalent Fab assemblies and correlates with neutralization activity, whereas sparse glycan environments on SARS‐CoV‐2 and Lassa virus favor weak or heterogeneous engagement without neutralization. Structural and mutational analyses further demonstrate that homotypic Fab–Fab interactions stabilize multivalent engagement and contribute to neutralizing activity. Together, these findings define a structural framework in which viral glycan organization constrains antibody valency and engagement, while somatic hypermutation contributes to the acquisition of homotypic Fab‐Fab interactions that facilitate multivalent recognition of viral glycan shields.

## Graphical abstract

Canonical Y‐shaped antibodies recognize viral glycan shields through adaptive Fab assembly states shaped by glycan organization and somatic hypermutation. Structural analyses ofbroadly neutralizing antibodies VRC35 and VRC36 across glycoproteins of HIV‐1, influenza, SARS‐CoV‐2, and Lassa viruses reveal distinct Fab assembly states, spanning monovalent, intra‐IgG, and inter‐IgG assemblies. Somatic hypermutation promotes homotypic Fab–Fab interactions that stabilize multivalent binding and contribute to neutralization.

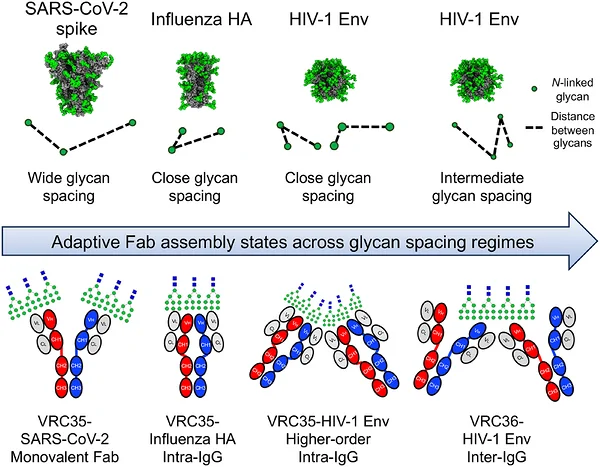

## 1 Introduction

Viral entry glycoproteins from diverse viruses, including HIV‐1 envelope (Env), influenza hemagglutinin (HA), SARS‐CoV‐2 spike, and Lassa virus (LASV) glycoprotein complex (GPC), are extensively coated with *N‐*linked glycans. These host‐derived glycans create a shielding layer that obscures underlying protein epitopes and facilitates immune evasion [1, 2]. Despite this barrier, the immune system can generate responses that target glycan‐rich surfaces, in some cases achieving broad and potent neutralization. Antibody recognition may involve composite protein–glycan interfaces or predominantly glycan epitopes, although such interactions are often limited by the relatively weak interactions between antibodies and carbohydrate moieties [3, 4].

Prior studies have described glycan‐reactive antibodies with atypical architectures that enable multivalent engagement of clustered glycans [5, 6, 7]. One well‐characterized example is the domain‐swapped broadly neutralizing antibody (bNAb) 2G12, in which heavy‐chain variable domains are exchanged between the two Fab arms, generating an I‐shaped architecture that enables cross‐neutralization of both HIV‐1 and influenza A viruses [8, 9]. In addition, Fab‐dimerized glycan‐reactive (FDG) I‐shaped antibodies, which do not undergo domain swapping, can adopt non‐canonical Fab arrangements that facilitate neutralization of HIV‐1 and binding to other glycosylated viral proteins [10]. These architectures expand the effective binding surface and promote multivalent engagement of clustered glycans. However, such non‐canonical configurations are rare and do not reflect the architecture of most circulating antibodies, which have a canonical Y‐shaped form. This raises a fundamental question of how canonical antibodies engage densely glycosylated viral surfaces, particularly in cases where recognition is dominated by glycans.

Although antibodies can engage antigens multivalently [11, 12, 13, 14], the mechanisms by which this occurs in heterogeneous glycan environments remain incompletely defined. Viral surfaces exhibit substantial variation in glycan density and spatial organization, suggesting that antibody engagement may be influenced not only by glycan identity but also by their geometric arrangement. However, it is unclear how glycan spacing influences the valency and mode of antibody binding. In addition, how antibodies themselves adapt to such glycan environments is not well defined, including whether somatic hypermutation can generate homotypic Fab–Fab interactions that enable multivalent binding on densely glycosylated surfaces. Moreover, the structural relationship between glycan organization and antibody binding mode has not been well characterized.

Here, we investigate how canonical Y‐shaped antibodies recognize glycan‐rich viral glycoproteins using two human antibodies, VRC35 and VRC36, isolated from an HIV‐1‐infected donor. By combining cryo‐electron microscopy (cryo‐EM) structural determination, binding analyses, and mutational studies across multiple viral systems with distinct glycan densities, including the HIV‐1 Env, influenza HA, SARS‐CoV‐2 spike, and LASV GPC, we show that these antibodies adopt distinct binding modes associated with differences in glycan organization. Fab engagement spans monovalent binding to dimeric and higher‐order assemblies, enabled by somatic hypermutation‐dependent homotypic Fab–Fab interactions that occur in both intra‐ and inter‐immunoglobulin G (IgG) configurations. Together, these results demonstrate that distinct Fab assembly states are associated with glycan organization, while somatic hypermutation promotes homotypic Fab–Fab interactions that facilitate multivalent recognition of viral glycan shields.

## 2 Results

### 2.1 VRC35 Forms Distinct Fab Assemblies Dictated by Viral Glycan Geometry

Three antibody families, VRC35, VRC36, and VRC37, were isolated from a person living with HIV‐1, donor N26, using high throughput B cell culture or by single‐cell sorting with scaffolded glycan‐V3 probes (Figure S1a–c). Among these, VRC35 and VRC36 exhibited broad HIV‐1 neutralizing activity and glycan‐reactive properties and were therefore selected for detailed structural and functional characterization in this study. In contrast, VRC37 showed limited neutralization breadth and was not investigated further (Figure S1d).

Members of the VRC35 family of antibodies were isolated from memory B cells of an HIV‐1‐infected individual by high‐throughput B cell culture or by single‐cell sorting using scaffolded glycan‐V3 probes [15, 16, 17] (Figure 1a and Figure S1a,b). The lineage, derived from IGHV4‐34^*^02 and IGKV2‐24^*^01, comprised three members with 28%–34% and 19%–20% divergence from germline in the heavy and light chains, respectively (Figure S1c and Table S1). The amplified heavy chains were IgG1. We selected antibody VRC35.01 (hereafter VRC35) for characterization. VRC35 neutralized ∼50% of a 208‐virus HIV‐1 panel with geometric mean IC50 of 2.08 µg/mL (Figure 1a and Figure S1d,e). As expected, its epitope mapped to the glycan‐V3 supersite: VRC35 competed with the glycan‐V3 bNAb 2G12 for Env binding (Figure 1b); its pattern of neutralization correlated with those of known glycan‐V3‐targeting bNAbs (Figure S2a); and its neutralization potency was reduced on Env‐pseudoviruses mutated to remove the glycan at position N332 (Figure S2b).

**FIGURE 1:**
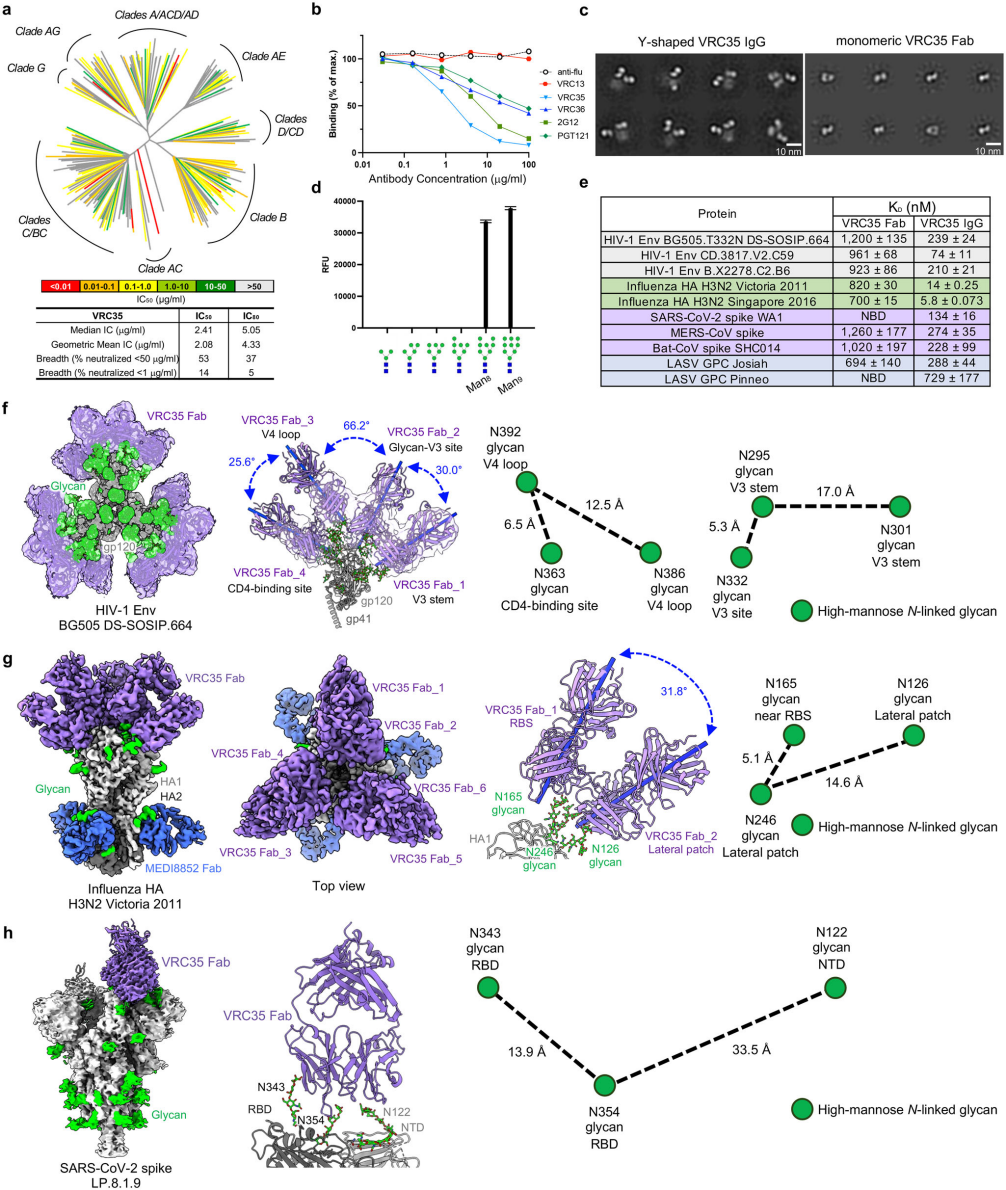
VRC35 adapts to distinct viral glycan landscapes. (a) Dendrogram of the 208‐strain virus panel colored by VRC35 IC50 titers. (b) VRC35 binding competition assay. Lines indicate binding of labeled VRC35 to TRO.11‐Env trimer‐expressing 293T cells in the presence of serially diluted unlabeled competitor antibodies as measured by flow cytometry. (c) Negative‐stain EM 2D class averages show canonical Y‐shaped VRC35 IgG and monomeric Fab. (d) Binding of VRC35 IgG to Man8 and Man9 glycans on a high‐density microarray. Error bars indicate standard deviation. (e) SPR analysis of VRC35 Fab and IgG binding to HIV‐1 Env, influenza HA, coronavirus spikes, and LASV GPC. (f) Cryo‐EM structure of HIV‐1 BG505.T332N DS‐SOSIP.664 Env bound by twelve VRC35 Fabs. Surface representations (left) and cartoon representations (center) illustrate four Fab binding sites per Env protomer, targeting the V3 stem, glycan‐V3 site, V4 loop, and CD4‐binding site. Dimeric Fabs exhibit rotational angles of ∼25°–30°. The glycan matrix (right) shows the Cα‐Cα distances between *N*‐linked glycans. (g) Cryo‐EM structure of influenza HA (H3N2 Victoria 2011) bound by six VRC35 Fabs and three MEDI8852 Fabs. Surface representations (left and center) show side and top views of the complex, whereas the cartoon representation (right) highlights the dimeric VRC35 Fab assembly. VRC35 Fab_1 is positioned adjacent to the receptor‐binding site (RBS) and engages glycan N165, whereas VRC35 Fab_2 is located near the lateral patch and engages glycans N126 and N246, forming a dimer with a 31.8° rotational angle. The glycan matrix (far right) shows the Cα‐Cα distances among N126, N165, and N246. (h) Cryo‐EM structure of SARS‐CoV‐2 spike (LP.8.1.9) bound by a single VRC35 Fab. The surface representation (left) shows the overall Fab‐bound spike complex, whereas the cartoon representation (center) highlights the VRC35 interaction with glycans N343 and N354 on the receptor‐binding domain (RBD) and glycan N122 on the N‐terminal domain (NTD) of an RBD‐down protomer. The glycan matrix (right) shows the Cα‐Cα distances among N343, N354, and N122.

Negative‐staining electron microscopy (NS‐EM) and sedimentation equilibrium analytical ultracentrifugation (AUC) showed that VRC35 IgG adopted a canonical Y‐shaped architecture and that Fab was monomeric (Figure 1c and Figure S3a). A high‐density glycan microarray binding analysis revealed exclusive binding to oligomannose glycans Man_8_GlcNAc_2_ (Man_8_) and Man_9_GlcNAc_2_ (Man_9_) (Figure 1d and Figure S4a,b). Consistent with the polyreactivity typical of VH4‐34 antibodies [18], VRC35 IgG showed low‐level reactivity in HEp‐2 cell staining and no detectable cardiolipin binding (Figure S5a,b). By surface plasmon resonance (SPR), VRC35 Fab bound HIV‐1 Env, influenza HA, coronavirus spike, and LASV GPC with micromolar affinity, whereas the IgG form displayed ∼10‐fold higher avidity (Figure 1e).

Strikingly, cryo‐EM analysis of HIV‐1 Env BG505.T332N DS‐SOSIP.664 in complex with VRC35 Fab revealed 12 Fabs bound per trimer (Figure 1f, Figure S6, and Table S4), a stoichiometry confirmed by AUC and isothermal titration calorimetry (ITC) (Figures S3c,d, and S7a). Because VRC35 weakly neutralized strain BG505.T332N, we also determined cryo‐EM structures of potently neutralized clade B, C, and CD strains (CD.3817.V2.C59, C.6785.V5.C14, B.X2278.C2.B6, and B.CNE14), which consistently revealed 2D class averages compatible with 12 Fabs per Env trimer (Figure S8a–c and Tables S2 and S3). Each protomer accommodated four Fabs targeting the V3 stem, glycan‐V3 site, V4 loop, and CD4‐binding site. Pairs of adjacent Fabs formed homotypic contacts with rotational angles of ∼25°–30°. Based on the reported HIV‐1 Env glycan profile of this trimer [19, 20], *N*‐linked oligomannose including N295, N301, N332, N363, N386, and N392 were positioned to interact with two pairs of VRC35 dimeric Fabs. Analysis of the relative spatial arrangement of glycan anchoring sites on the Env surface (here referred to as the glycan matrix) showed that N295‐N332 and N363‐N392 were separated by 5.3 and 6.5 Å, respectively, distances observed in the context of dimeric Fab assemblies, whereas longer distances (12.5 and 17 Å) were measured in neighboring regions bound by individual Fabs (Figure 1f).

Cryo‐EM structure of VRC35 Fab bound to influenza H3N2 HA (A/Victoria/361/2011) revealed three pairs of dimeric Fabs on the HA head (Figure 1g, Figure S9, and Table S5). Within each pair, one Fab was positioned near the receptor‐binding sites (RBS) and the other adjacent to the lateral patch. Localized refinement and distance constraints identified three *N*‐linked glycans (N126, N165, N246) as key targets. The glycan pair N165‐N246 was separated by 5.1 Å and was associated with a dimeric Fab assembly, whereas N126‐N246 was separated by 14.6 Å and was observed in a region occupied by an individual Fab. Liquid chromatography‐tandem mass spectrometry (LC‐MS/MS) analysis determined that these glycans were of the high‐mannose type. N126 carried Man_8_ as the major type, and N165 and N246 were heavily enriched in Man_9_ (Figure S10a–c). The rotational angle of VRC35 Fabs on HA (31.8°) was similar to that observed on HIV‐1 Env, indicating conserved yet flexible homotypic assembly.

In contrast, VRC35 bound SARS‐CoV‐2 spike (LP.8.1.9) with a single Fab on each protomer (Figure 1h, Figure S11, and Table S6). Cryo‐EM analysis placed the bound VRC35 Fab near the receptor‐binding domain (RBD) glycans at positions N343 and N354, as well as the N‐terminal domain (NTD) glycan N122, with this arrangement observed only on the RBD‐down protomer. In 2D classification, up to three VRC35 Fabs were observed bound to the spike, however, one Fab showing stronger density, whereas the two exhibiting weaker densities, indicating heterogeneous and transient engagement.

Site‐specific glycosylation analysis of the LP.8.1.9 spike showed that N122 and N354 were enriched in high‐mannose glycans, including Man_8_ and Man_9_, whereas N343 was predominantly occupied by Man_5_ with only limited amounts of Man_8_ and Man_9_ (Figure S10d–f), broadly consistent with the glycan‐binding preference of VRC35 (Figure 1d and Figure S4a,b). Because the local resolution of the VRC35 Fab density was limited, the structural model identified glycans in proximity to the antibody but did not permit assignment of the specific mannose species contacted at the individual glycosylation sites. The Cα‐Cα distance between N354 and N122 was 33.5 Å, substantially longer than the inter‐glycan distances observed in regions supporting homotypic Fab–Fab assemblies. Consistent with this spacing, VRC35 was observed as a single Fab bound to the spike surface in the corresponding structure.

Similarly, the cryo‐EM structure of LASV GPC (Josiah strain) bound by VRC35 revealed one dominant Fab density and additional weak Fab densities (Figures S12 and S13 and Table S7). LASV glycan profile [21] showed four neighboring *N*‐linked oligomannose, including N79 on GP1 and N365, N373, and N395 on GP2, that could contribute to VRC35 interaction. Among these, N79 and N365 were enriched in Man_8_ and Man_9_, making them the most plausible contributors. However, these glycans were spaced 15.5–30.4 Å apart, far exceeding the 5–6 Å spacing compatible with dimeric Fab pairing (Figure S13). This sparse glycan arrangement supported heterogeneous VRC35 engagement on LASV GPC, analogous to that observed on the SARS‐CoV‐2 spike (Figure 1h).

Collectively, these structures demonstrate that VRC35 adopts distinct Fab assembly states across diverse viral glycan landscapes, ranging from monovalent binding to dimeric and higher‐order assemblies on densely glycosylated surfaces. These observations indicate that antibody engagement is shaped by the spatial organization of glycans, with closely spaced glycans promoting homotypic Fab–Fab interactions and multivalent assembly, whereas more widely spaced glycans favor monovalent and heterogeneous binding.

### 2.2 Homotypic Fab–Fab Interactions Stabilize Multivalent Binding and Enable Neutralization

While VRC35 formed multivalent assemblies on HIV‐1 Env and influenza HA, it engaged SARS‐CoV‐2 spike and LASV GPC only weakly and heterogeneously. To determine whether this broad glycan‐binding activity translated into antiviral function, we evaluated VRC35 neutralization across heterologous viruses. Although VRC35 bound the entry glycoproteins of LASV and coronaviruses (Figure 1e), it did not neutralize LASV or SARS‐CoV‐2 variants (Figure S14c,d). In contrast, VRC35 neutralized a wide range of H3N2 influenza A viruses spanning four decades of isolation, from A/Philippines/2/1982 to A/Massachusetts/18/2022 (Figure 2a and Figure S14a,b).

**FIGURE 2:**
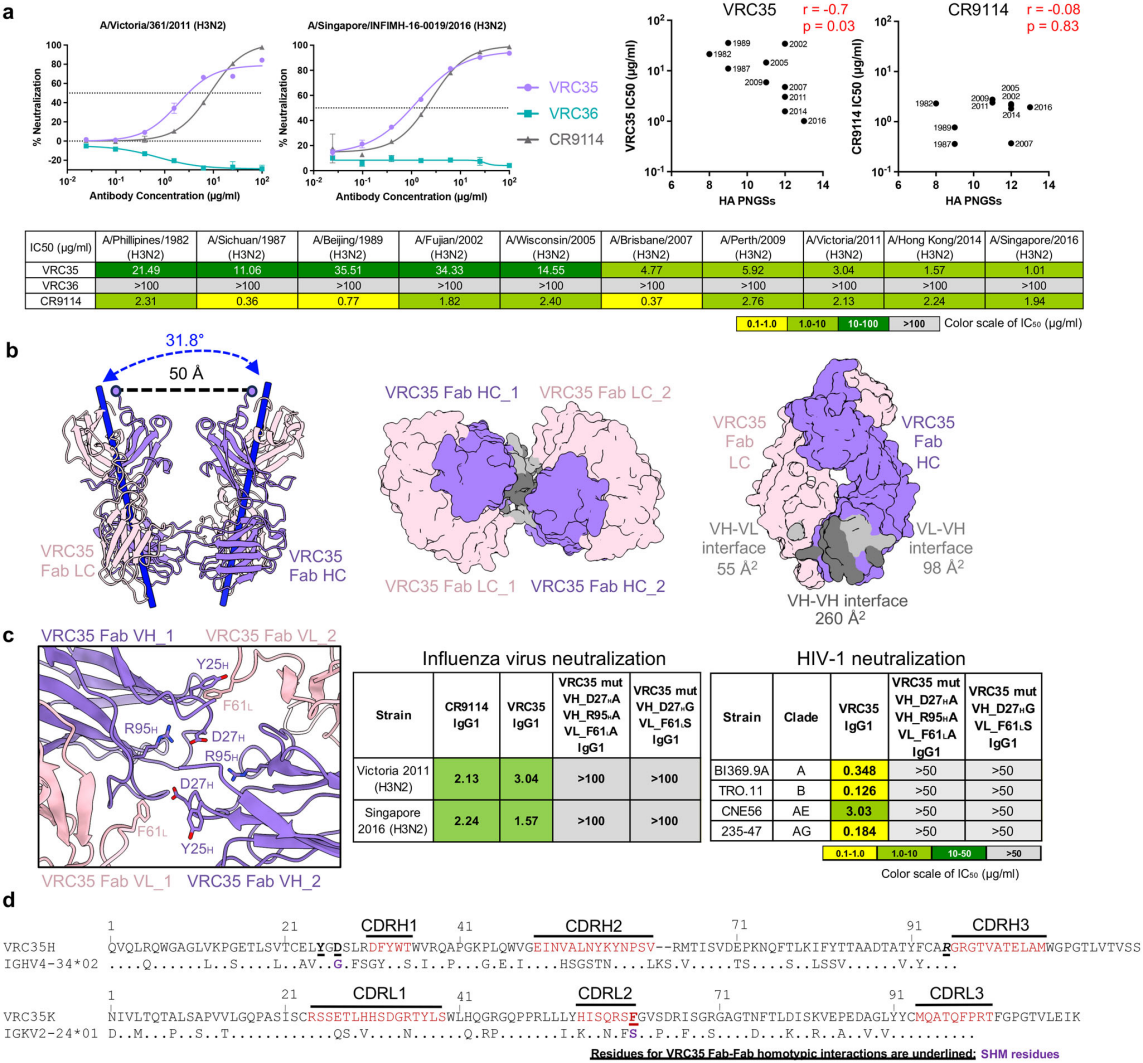
VRC35 neutralizes highly glycosylated viruses through homotypic assembly. (a) VRC35 neutralizes multiple influenza H3 strains in a reporter virus assay. Values are shown as IC_50_ (µg/mL). Spearman's rank correlation between the number of predicted *N*‐linked glycosylation sites (PNGSs) on HA and neutralization IC_50_ values revealed a significant correlation for VRC35 but not for CR9114. (b) Dimeric VRC35 Fabs are oriented at a 31.8° rotation and separated by ∼50 Å between CH1 termini. VH‐VH and VH‐VL homotypic interfaces are shown in dark and light gray, respectively, with buried surface areas (Å [2]) indicated. (c) Key residues mediating VRC35 Fab–Fab homotypic interactions. Structural model of the VRC35 homotypic interface (left). Neutralization analyses of alanine substitutions (D27A, R95A, and F61A) and germline reversion mutations (D27G and F61S) demonstrated marked reductions in neutralization against representative influenza and HIV‐1 strains. Values are shown as IC_50_ (µg/mL). (d) Sequence alignment of mature and germline VRC35 heavy and light chains. Complementarity‐determining regions (CDRs) are highlighted in red. Residues involved in homotypic interactions are underlined, and somatic hypermutation (SHM) residues contributing to these interactions are shown in purple.

Given that VRC35 recognized closely spaced glycan clusters on HIV‐1 Env and influenza HA, we hypothesized that its neutralization potency would correlate with glycan density. Consistent with this hypothesis, VRC35 neutralization potency (IC_50_ values) significantly correlated with the number of potential *N*‐linked glycosylation sites (PNGSs) on HA (Spearman r = −0.7, p = 0.03), indicating that increased glycan density enhanced VRC35 sensitivity among H3N2 isolates (Figure 2a). In contrast, no such correlation was observed for CR9114, a broadly neutralizing HA stem‐directed control antibody that binds independently of glycan [22]. Together, these results showed that VRC35 cross‐neutralized highly glycosylated influenza A viruses in a glycan‐dependent manner, and its potency correlated with HA PNGSs.

To define the structural basis for this glycan‐geometry dependence, we analyzed the dimeric VRC35 Fabs. The C‐termini of the heavy chains were separated by ∼50 Å, indicating that the two Fabs could originate from the same IgG1 and formed an intra‐IgG Fab–Fab homotypic assembly (Figure 2b). These interactions were primarily mediated by heavy‐chain variable domains, burying ∼260 Å^2^ of surface area. Minor contacts between VH‐VL regions contributed an additional 55 and 98 Å^2^ on the light and heavy chains, respectively. Using AlphaFold‐predicted VRC35 structures fitted into local cryo‐EM density, we identified the residues comprising the homotypic interface (Figure 2c,d). A pair of hydrophobic residues Y25_H_ and F61_L_ formed an outer hydrophobic surface, while hydrophilic residues D27_H_ and R95_H_ generated a central hydrophilic core. Together, these features were predicted to stabilize the dimeric VRC35 Fabs.

To assess the functional importance of this interface, we mutated contact residues including the hydrophobic residue (F61_L_) and hydrophilic residues (D27_H_ and R95_H_) to alanine. Neutralization assays using live influenza reporter viruses and a multi‐clade panel of HIV‐1 Env pseudoviruses showed that the combined mutant exhibited a marked loss of activity, supporting an important role for the Fab–Fab homotypic interface in neutralization (Figure 2c). Sequence comparison of mature and germline VRC35 chains revealed that interface residues D27 and F61 were acquired through somatic hypermutation (SHM) (Figure 2d). To directly assess the contribution of these SHM‐derived residues, we reverted D27 and F61 to their corresponding germline amino acids (D27_G_ and F61_S_). The combined germline‐reversion mutant completely abolished neutralization activity (Figure 2c), suggesting that SHM‐derived residues contribute to homotypic assembly and neutralization activity.

Together, these results indicated that VRC35 neutralization in multiple viral contexts is facilitated by a homotypic interface that enables multivalent engagement of viral glycan shields. The intra‐IgG Fab–Fab homotypic assembly therefore provides a structural basis for enhanced functional avidity, linking multivalent glycan engagement to effective viral neutralization.

### 2.3 Inter‐IgG Homotypic Assembly Enables Glycan‐Dependent Multivalent Engagement by VRC36

Unlike VRC35, which derived from a VH4‐34 lineage and displayed low‐level polyreactivity typical of that gene family [18], the VRC36 family of antibodies originated from a distinct VH3‐15/VK3‐11 lineage isolated from the same HIV‐1 donor (Figure S1a–c and Table S1). The most potent member, VRC36.01 (hereafter VRC36), was selected for further characterization. Despite a comparable level of somatic mutation, VRC36 exhibited greater HIV‐1 breadth than VRC35 (66% at IC_50_< 50 µg/mL) across a 208‐virus HIV‐1 pseudovirus panel [23], and showed no detectable autoreactivity, as assessed by HEp‐2 cell staining and cardiolipin binding assays (Figure 3a, Figures S1d,e and S5a,b). Competition profiling confirmed that VRC36 targeted the glycan‐V3 supersite, but its genetic origin and functional properties suggested different requirements for glycan engagement (Figure 3b and Figure S2).

**FIGURE 3:**
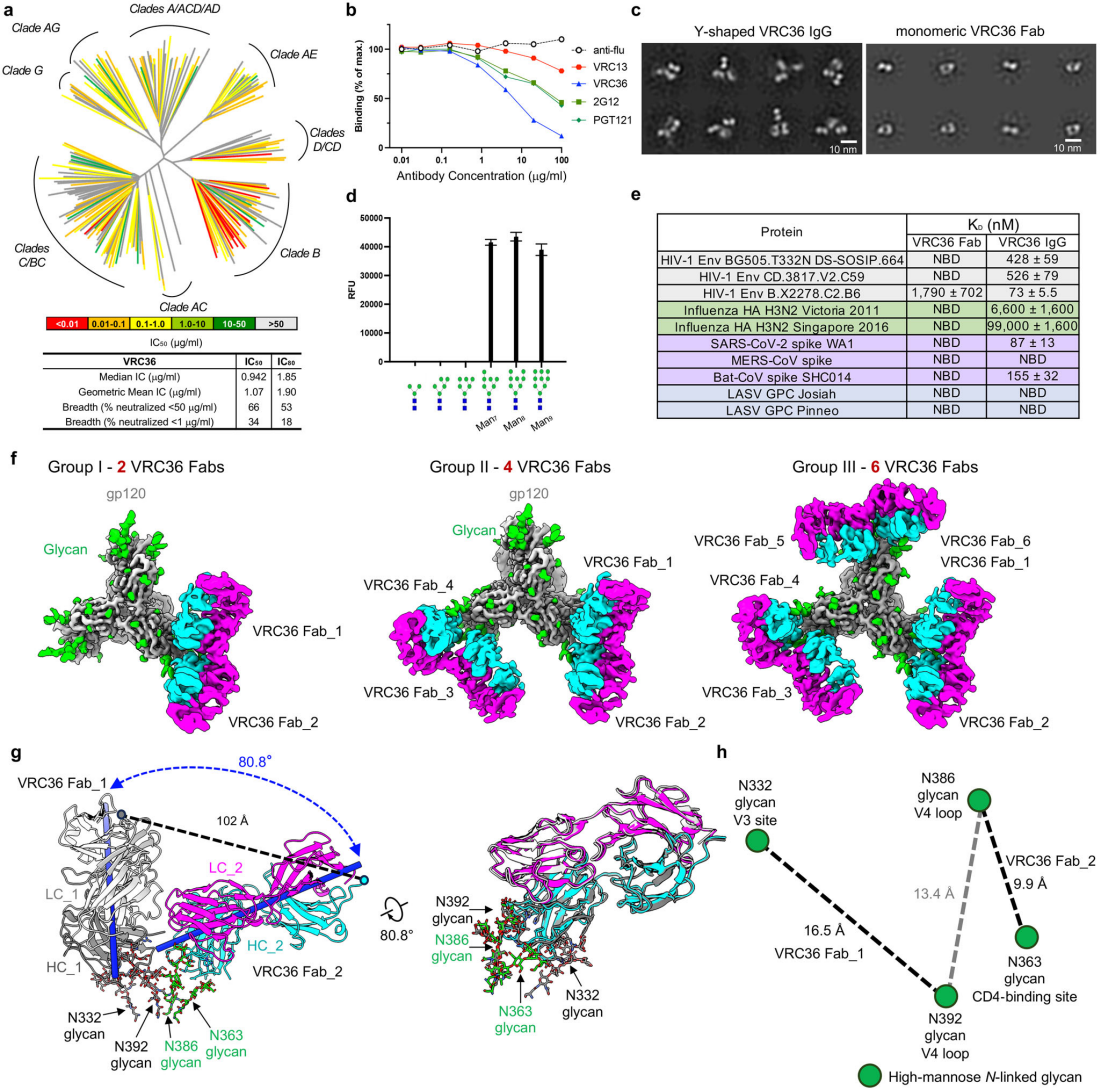
VRC36 forms inter‐IgG homotypic assemblies on HIV‐1 Env. (a) Neutralization dendrogram of the 208‐strain virus panel colored by VRC36 IC_50_ values. (b) VRC36 binding competition assay. Lines indicate binding of labeled VRC36 to TRO.11‐Env trimer‐expressing 293T cells in the presence of the indicated serially diluted unlabeled competitor antibodies as measured by flow cytometry. (c) Negative stain‐EM 2D class averages show canonical Y‐shaped VRC36 IgG and monomeric Fab. (d) Binding of VRC36 IgG to Man7, Man8 and Man9 glycans on a high‐density microarray. Error bars indicate standard deviations. (e) SPR analysis of VRC36 Fab and IgG binding to HIV‐1 Env, influenza HA, coronavirus spikes, and LASV GPC. (f) Cryo‐EM analysis revealed dimerized VRC36 Fabs bound to BG505 DS‐SOSIP.664 Env. Fabs bound to Env are classified into three groups with 2, 4, or 6 Fabs per Env trimer. The VRC36 Fab heavy chain (HC) and light chain (LC) are colored cyan and magenta, respectively. HIV‐1 Env gp120 and gp41 are shown in light gray and dark gray. Glycans are shown in green. (g) The dimeric VRC36 Fabs are oriented at an 80.8° rotation and separated by ∼102 Å between CH1 termini. Each Fab interacted with two glycans. After rotation, paired VRC36 Fabs and their interacting glycans align with each other. (h) Glycan matrix of N332, N392, N386, and N363. The Cα‐Cα distance between N332 and N392 is 16.5 Å, supporting VRC36 Fab_1 binding. The Cα‐Cα distance between N386 and N363 is 9.9 Å, supporting VRC36 Fab_2 binding. The observed 13.4 Å distance between merged glycans N386 and N392 corresponds to the homotypic interactions formed between two separate IgGs.

VRC36 also differed from VRC35 in its biophysical behavior. Although both antibodies adopted the canonical Y‐shaped IgG architecture and were monomeric as Fabs (Figure 3c and Figure S3b), and both recognized high‐mannose glycans with VRC36 binding Man_7_, Man_8_, and Man_9_ on a glycan microarray (Figure 3d and Figure S4a,c), VRC36 displayed a stronger avidity gain upon IgG bivalency: the Fab showed weak or undetectable binding to HIV‐1 Env, whereas the IgG bound with measurable affinity (Figure 3e). Moreover, the IgG, but not the Fab, bound influenza HA and coronavirus spikes, suggesting that VRC36 relied more heavily on multivalent engagement than VRC35.

Cryo‐EM analysis of VRC36 Fab in complex with HIV‐1 BG505.T332N DS‐SOSIP.664 Env revealed dimeric VRC36 Fab assemblies on Env. Based on the occupancy of the three symmetrical Fab binding sites, three major classes containing 2, 4, or 6 Fabs per trimer were identified (Figure 3f, Figure S15, and Table S8), representing distinct Fab occupancy states. The average occupancy of approximately three Fabs per Env trimer was consistent with AUC and ITC measurements (Figures S3c,d, and S7b). The distance between Fab heavy‐chain C‐termini was ∼102 Å, indicating that the two Fabs originated from different IgGs and thus formed an inter‐IgG homotypic assembly (Figure 3g).

The dimeric VRC36 Fabs engaged *N*‐linked glycans near the glycan‐V3 site. In each dimer, one Fab (Fab_1) contacted glycans N332 and N392, whereas the other (Fab_2) bound N363 and N386 (Figure 3g,h). Notably, the two dimeric VRC36 Fabs maintained a rotational angle of 80.8°, which aligned glycan N392 from VRC36 Fab_1 with glycan N386 from VRC36 Fab_2. However, N332 from VRC36 Fab_1 and N363 from VRC36 Fab_2 did not align and contacted different faces of the VRC36 heavy chain. Analysis of the glycan matrix revealed Cα‐Cα distances of 16.5 Å (N332‐N392), 9.9 Å (N386‐N363), comparable to those observed for VRC35. The distance between the aligned glycans N386 and N392 was 13.4 Å, representing the glycan arrangement observed in the inter‐IgG homotypic assembly (Figure 3h).

Together, these findings demonstrated that VRC36 engaged the HIV‐1 Env trimer through inter‐IgG homotypic assembly, distinct from the intra‐IgG assembly observed for VRC35. The glycan arrangement observed on Env supports this inter‐IgG configuration and expands the range of observed Fab assembly states.

### 2.4 Structural Basis of Glycan Recognition and Inter‐IgG Homotypic Fab–Fab Assembly

Glycan recognition by VRC36 was mediated primarily by the heavy chain. For the aligned glycans at positions N386 and N392, the same sets of CDR residues from both Fabs participated in binding, including N31_H_ and W33_H_ from CDRH1, D56_H_ from CDRH2, and H100_H_ and E103_H_ from CDRH3. Because these residues were used symmetrically by both Fabs upon superimposition, we refer to them here as “recurrent” glycan‐contact residues. In contrast, glycans that did not align between the two Fabs were engaged by distinct framework residues, including Y7_H_ from FR1 and D75_H_, R78_H_, and R80_H_ from FR3 contacted the non‐aligned glycan N332, while residues G26_H_‐V30_H_ from FR1 and D79_H_ from FR3 engaged non‐aligned glycan N363 (Figure 4a).

**FIGURE 4:**
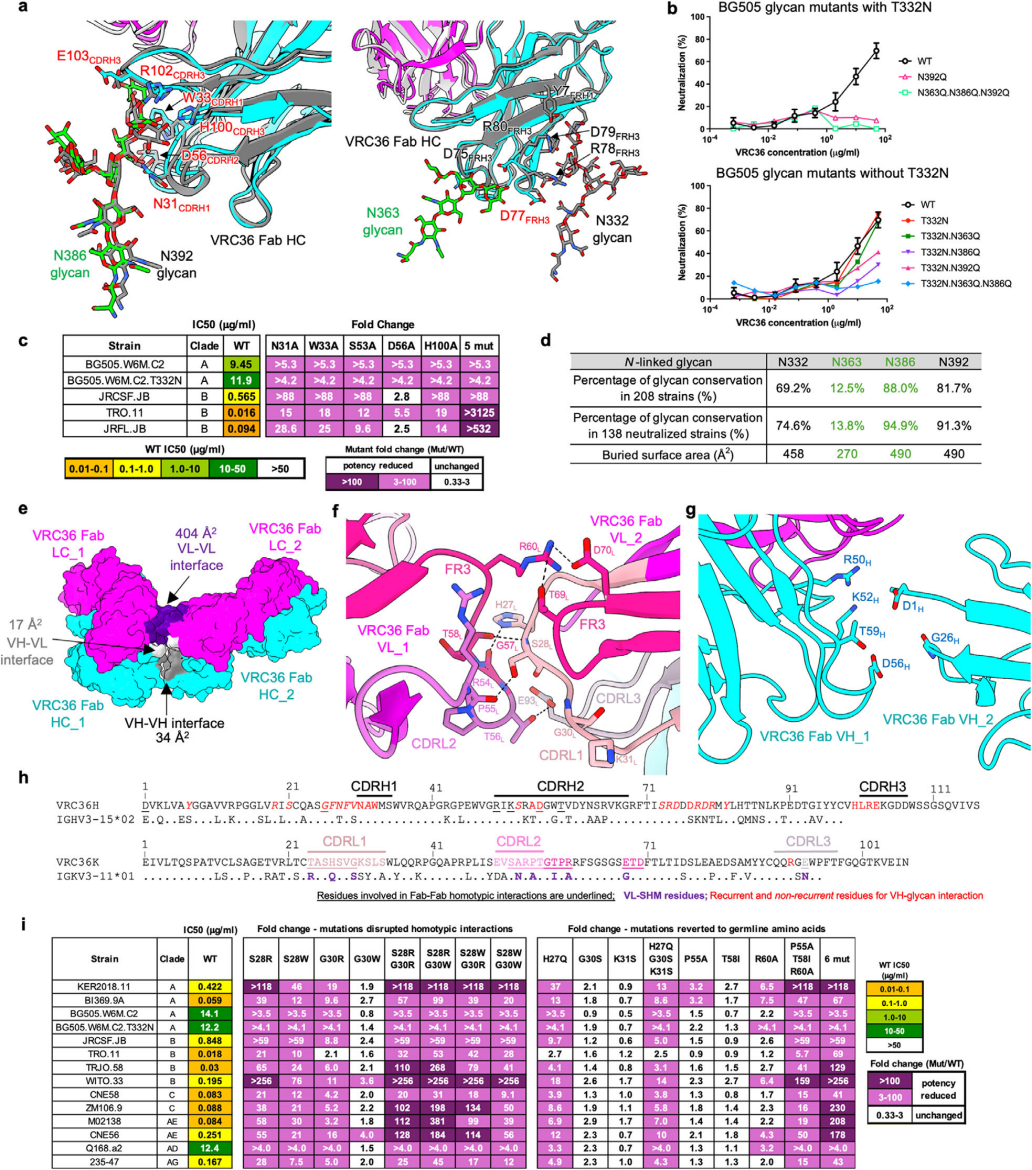
Inter‐IgG homotypic assembly contributes to VRC36 neutralization of HIV‐1. (a) Superimposition of N386 and N392. Two VRC36 Fabs use the same residues from CDRH1, CDRH2, and CDRH3 to interact with glycans N386 and N392. N363 and N332 are engaged by non‐repeated residues from FR1 and FR3 of the heavy chain. (b) Neutralization assessments of BG505 glycan mutants with and without T332N. (c) Neutralization assessments of VRC36 IgG mutants. (d) Conservation analysis of N332, N363, N386, and N392 glycans across 208 HIV‐1 strains and 138 neutralized strains, with corresponding buried surface areas. (e) Surface representation of dimeric VRC36 Fabs. VH‐VH, VH‐VL and VL‐VL homotypic interfaces are shown in dark gray, light gray, and purple, respectively. Buried surface areas (Å [2]) are indicated. Hydrogen bonds and salt bridges at the VL‐VL interface are shown as dotted lines. (f) Interactions at the VRC36 VL‐VL interface. Hydrogen bonds and salt bridges are shown as dotted lines. (g) Interactions at the VRC36 VH‐VH interface. Amino acid proximity indicates buried surface shown in (e). Hydrogen bonds and salt bridges were not observed. (h) Sequence alignment of mature and germline VRC36 heavy and light chains. Residues involved in Fab‐Fab homotypic interactions are underlined; residues involved in VH‐glycan interactions are colored red, and non‐recurrent residues are italicized. SHM residues involved in homotypic interactions are shown in bold purple. (i) Neutralization characteristics of VRC36 light chain mutants. Values are shown as fold change (Mut/WT). Left, mutations to disrupt or enhance VL‐VL homotypic interactions. Right, mutations to revert to germline amino acids. 6 mut indicates the mutations including H27Q, G30S, K31S, P55A, T58I, and R60A.

To assess the functional importance of these glycans, we generated a panel of BG505 Env pseudoviruses with altered *N*‐linked glycosylation. The wild‐type and T332N variants were equally sensitive to VRC36, whereas removal of glycans at N392 alone, or together with other glycans, completely abrogated neutralization. In the presence of the N332 glycan, deletion of N386 or N392 reduced sensitivity, while the N363 glycan was dispensable (Figure 4b and Figure S2b). While the presence of particular glycans affected neutralization potency, the total number of PNGSs did not correlate with neutralization sensitivity (Figure S2c). Consistent with these findings, alanine substitution of the recurrent heavy‐chain residues (N31_H_, W33_H_, D56_H_, H100_H_) or the non‐recurrent residue (S53_H_) impaired neutralization by 2.5‐ to >88‐fold, and the combined mutant lost activity against even the most sensitive HIV‐1 strains TRO.11 and JRFL.JB, identifying key residues required for glycan recognition (Figure 4c). Conservation analysis across the 208‐virus panel showed that the aligned glycans N386 and N392 were highly conserved (>80% of all strains; >90% of neutralized strains), whereas the non‐aligned glycans N332 and N363, which were not important for BG505 neutralization, were less conserved (74.6% and 13.8% in neutralized strains, respectively) (Figure 4d). Buried surface area analysis was consistent with these observations: the aligned glycans N386 and N392 buried 490 Å^2^ of surface, compared with 458 Å^2^ for N332 and 270 Å^2^ for N363. These results indicated that glycans with greater burial and higher conservation were most critical for neutralization.

The glycan‐induced inter‐IgG Fab–Fab homotypic assembly included three interfaces: light‐light, heavy‐heavy, and heavy‐light. The light‐light contact dominated, burying ∼404 Å^2^ of surface area, whereas the heavy‐heavy and heavy‐light interfaces contributed only 34 and 17 Å^2^, respectively (Figure 4e). At the light‐light surface, hydrogen bonds from CDRL2 and FR3 in Fab_1 and CDRL1 in Fab_2 formed the principal interactions, which included R60_L_ from FR3 with T69_L_ and D70_L_ from FR3, T58_L_ from FR3 with S28_L_ from CDRL1, T56_L_ from CDRL2 with E93_L_ from CDRL3, and R54_L_ from CDRL2 with S28_L_ from CDRL1 (Figure 4f). At the heavy chain‐heavy chain interface, several polar residues were present, but no hydrogen bonds were observed (Figure 4g). To determine whether these key interactions were encoded in the germline variable heavy and light chain genes or arose via somatic hypermutation (SHM), we compared the germline and mature antibody sequences at the contact sites. SHMs were mainly observed in the light chain‐light chain interface (Figure 4f,h). Overall, there was 26% (nucleotide) SHM on heavy chain and 20% (nucleotide) SHM on light chain (Figure S1c). To assess the functional importance of these contacts, mutations were introduced to disrupt the interface. Substituting small residues (S28_L_, G30_L_) with bulky side chains such as arginine or tryptophan reduced neutralization potency, particularly at S28_L_, which was located at the center of the interface (Figure 4f,i). We also assessed the impact of reverting SHM‐derived residues. While most single reversions produced modest effects, combinations of SHM reversions substantially reduced VRC36 neutralization, supporting a contribution of SHM‐derived residues to homotypic assembly and neutralization potency (Figure 4i). H27 directly participated in the VL‐VL interface, whereas G30 and K31 were SHM‐derived residues located adjacent to the interface and were included as internal comparisons. Consistent with the structural analysis, residues with greater interface involvement generally produced larger effects on neutralization upon germline reversion. For example, H27 and R60 exhibited greater buried surface areas and stronger functional effects, whereas G30, K31, P55, and T58 showed comparatively smaller structural and functional contributions (Figure 4f,h,i).

Together, these data define the structural basis of glycan recognition and inter‐IgG Fab‐Fab homotypic assembly by VRC36, a canonical Y‐shaped antibody, identifying key glycan‐contact residues and interface interactions that enable homotypic assembly and contribute to neutralization.

## 3 Discussion

Our study provides structural insight into how canonical antibodies engage glycan‐rich viral glycoproteins through context‐dependent multivalent binding. Across multiple viral systems, including HIV‐1, influenza, SARS‐CoV‐2, and Lassa virus, we observe that Fab binding modes range from monovalent engagement to higher‐order Fab assemblies (Figure 5a). These assembly states are associated with distinct patterns of glycan organization and are further supported by functional analyses, including neutralization and mutagenesis experiments.

**FIGURE 5:**
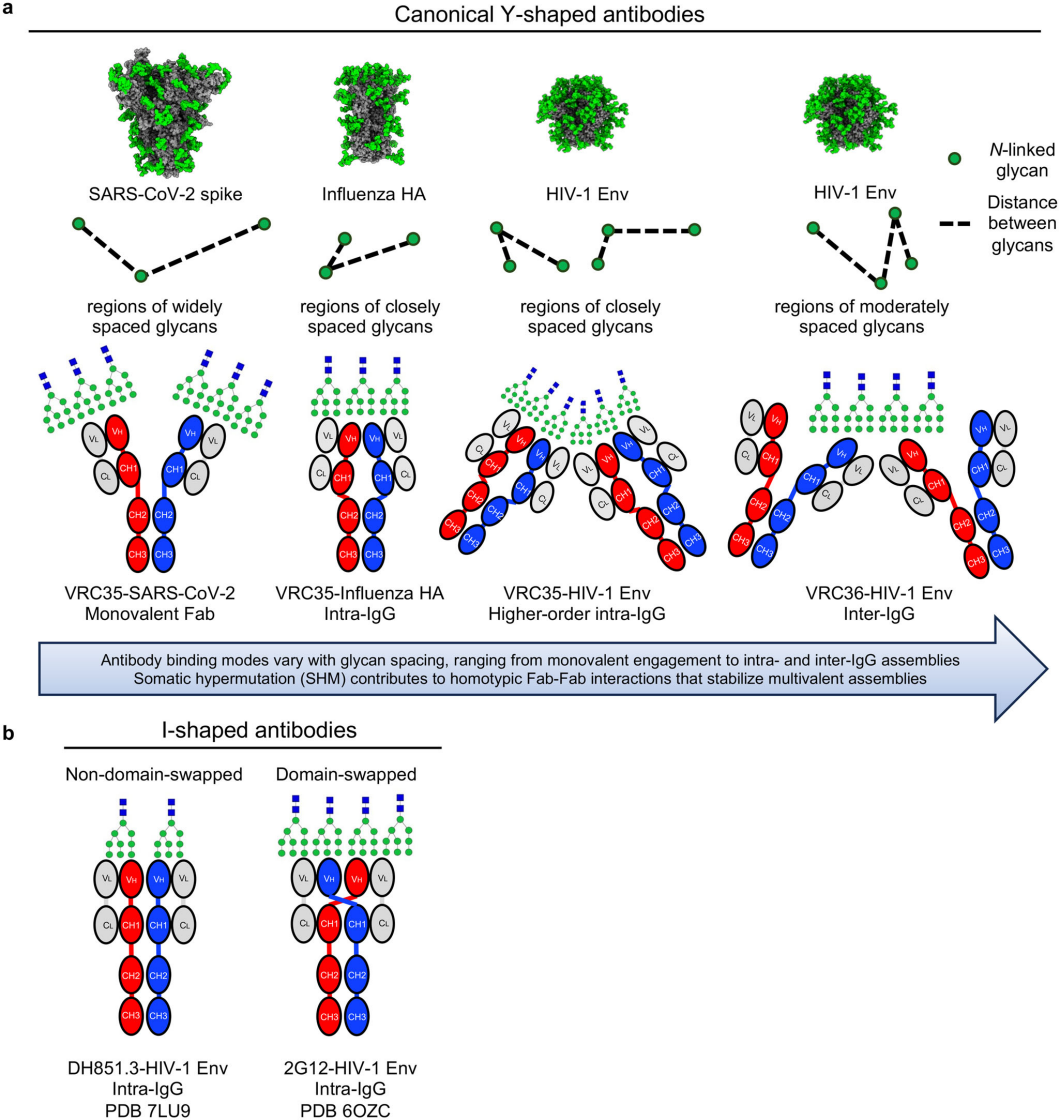
Structural models of glycan spacing‐dependent antibody binding modes. (a) Canonical Y‐shaped antibodies exhibit multiple binding configurations that vary with glycan organization on viral surfaces, reflecting the geometric constraints imposed by glycan spacing. Fully glycosylated models of SARS‐CoV‐2 spike (LP.8.1.9), influenza H3N2 hemagglutinin (Victoria 2011), and HIV‐1 Env (BG505 DS‐SOSIP.664) illustrate differences in glycan density and spatial distribution. Notably, heterogeneous glycan organization can occur across distinct regions of the same viral surface, as exemplified by HIV‐1 Env, which contains both closely spaced and more moderately spaced glycan regions. Across viral glycoproteins exhibiting different glycan spacing regimes, antibody engagement ranges from monovalent binding (widely spaced glycans) to intra‐IgG Fab‐Fab assemblies (closely spaced glycans), and to inter‐IgG assemblies at intermediate spacing. Representative models are shown for VRC35 bound to SARS‐CoV‐2 spike (single Fab), VRC35 bound to influenza hemagglutinin (intra‐IgG assembly), VRC35 bound to HIV‐1 Env (intra‐IgG with higher‐order assemblies), and VRC36 bound to HIV‐1 Env (inter‐IgG assembly). Antibody heavy chains are shown in red and blue to distinguish individual Fab molecules, whereas light chains are shown in gray. Green circles represent *N*‐linked glycans, and dashed lines indicate distances measured between neighboring glycans. These configurations illustrate that glycan organization constrains the range of accessible antibody binding modes. Fully glycosylated models were generated using GLYCAM‐Web prediction tools. (b) In contrast, I‐shaped glycan‐reactive antibodies adopt preconfigured Fab geometries that enable multivalent glycan engagement independent of glycan spacing. Examples include the non‐domain‐swapped antibody DH851.3 and the domain‐swapped antibody 2G12, both bound to HIV‐1 Env. Together, these models highlight distinct structural strategies for glycan recognition, in which canonical antibodies exhibit geometry‐dependent flexibility, whereas I‐shaped antibodies rely on preconfigured architectures. While glycan spacing provides a useful structural framework for interpreting these binding modes, additional factors such as glycan composition and epitope accessibility may also contribute.

Our results suggest that glycan organization acts as a structural constraint on antibody engagement. Densely glycosylated surfaces, such as those of HIV‐1 Env and influenza HA, were associated with multivalent Fab assemblies, whereas more sparsely distributed glycans on SARS‐CoV‐2 spike and LASV GPC were associated with predominantly monovalent or heterogeneous binding. Importantly, these structural observations were accompanied by functional differences across viral systems, including differences in neutralization sensitivity and the effects of glycan‐ and interface‐directed mutations.

In parallel, our results suggest somatic hypermutation contributes to multivalent engagement of glycan‐dense surfaces. Both VRC35 and VRC36 form homotypic Fab–Fab interactions that stabilize multivalent assemblies. Mutational analyses further indicate that these interfaces contribute to neutralizing activity, providing functional support for the biological relevance of the observed Fab assemblies. Notably, residues mediating these interfaces arise through somatic hypermutation and contribute to function, highlighting an additional layer of antibody optimization beyond antigen recognition. Together, these observations suggest that antibody recognition of glycan shields reflects an interplay between glycan organization and antibody maturation.

Homotypic Fab–Fab interfaces have previously been described in antibodies targeting the repetitive PfCSP [11, 12, 13] and CD20 [14], suggesting that such interfaces may represent a broader strategy for enhancing multivalent binding on repetitive or clustered epitopes. In this context, the structures described here extend these observations to glycan‐rich viral surfaces and demonstrate how canonical antibodies can achieve multivalent recognition without requiring unusual architectures. This is distinct from previously described glycan‐reactive antiviral antibodies such as 2G12 [8, 9] and FDG antibodies [10], which rely on non‐canonical architectures to achieve multivalent engagement (Figure 5b). The ability to form either intra‐ or inter‐IgG assemblies further illustrates the structural flexibility of canonical Y‐shaped antibodies in adapting to different viral glycan geometries.

While our analysis reveals an association between glycan spacing, Fab assembly, and neutralization, the extent to which glycan organization alone determines functional outcomes remains unresolved. Additional factors, including glycan composition, epitope accessibility, and viral surface dynamics, are likely to contribute. Future studies that directly manipulate glycan spacing or composition will be important to establish causal relationships.

In summary, our findings establish an association between viral glycan organization and canonical antibody binding modes, and demonstrate that somatic hypermutation contributes to homotypic Fab–Fab interactions that stabilize multivalent recognition of viral glycan shields. Together, these findings provide a structural framework for understanding how canonical antibodies recognize diverse viral glycan shields, with implications for antibody engineering and vaccine design targeting glycan‐rich viral surfaces.

## 4 Methods

### 4.1 Human Subjects

Peripheral blood mononuclear cells (PBMCs) and sera were obtained from HIV‐infected donor N26, a North American Caucasian male aged 39, who participated in an NIAID protocol (https://clinicaltrials.gov/study/NCT00029445; NIH IRB protocol 02‐I‐0086). At the time point studied, this donor was an antiretroviral therapy‐naïve slow progressor who had been diagnosed 18 years prior, was antiretroviral‐naïve, and exhibited broad serum neutralization, with viral load of 45,316 copies/µL, and 386 CD4+ T‐cells/µL, as previously described [17]. Normal human PBMC were collected under a separate protocol (https://clinicaltrials.gov/study/NCT00001846; NIH IRB protocol 99‐CC‐0168). All donors signed informed consent and protocols were approved by the National Institutes of Health (NIH) Institutional Review Board.

### 4.2 Cell Lines

Expi293F cells (RRID: CVCL_D615), TZM‐bl cells (RRID: CVCL_B478), NIH3T3‐mouse‐CD40L cells (3T3‐msCD40L, RRID: CVCL_1H10), and FreeStyle 293‐F cells (RRID: CVCL_D603) were used directly from commercial sources and cultured according to the manufacturer's directions and as described previously [24].

### 4.3 Titration of Flow Cytometry Reagents

Fluorophore‐conjugated antibodies specific to human leukocyte cell surface molecules were titrated on peripheral blood mononuclear cells (PBMC) obtained from healthy individuals as described previously [24]. Briefly, antibodies were diluted serially 2‐fold in PBS and mixed with PBMC for 30 min at 4°C, followed by washing in PBS and fixation in 0.05% paraformaldehyde in PBS. The appropriate staining titer was selected as the one that resulted in adequate staining separation over a non‐stained control sample.

### 4.4 Probe Preparation

For B‐cell probes, ST11 proteins were produced in cells treated with kifunensine or swainsonine. These proteins display a V3 miniprotein including glycans N301 and N332, on a heterologous scaffold. ST11‐kif was produced in the presence of kifunensine, and ST11‐swa in swainsonine, as described [25]. ST11‐kif was conjugated to streptavidin APC (Invitrogen) and ST11‐swa to streptavidin‐PE (Invitrogen), each at a molar ratio of 3:1 ST11: streptavidin. The streptavidin conjugate was added stepwise, one‐fifth volume at a time with mixing for 20 min at 4°C between additions.

### 4.5 B Cell Culture and Microneutralization Screening

For isolation of N26‐VRC35.01 and N26‐VRC36.01, .04, and .05, antigen‐specific B cells were isolated from cryopreserved peripheral blood mononuclear cells of donor N26 using a previously described strategy [26]. After 14 days of culture, supernatants were collected for antibody screening, and cells were preserved for downstream analysis. Neutralization activity was evaluated using a high‐throughput HIV‐1 pseudovirus assay following an established protocol (https://www.protocols.io/view/high‐throughput‐hiv‐1‐microneutralization‐assay‐j8nlk9p76v5r/v1). Pseudoviruses corresponding to strains BaL.01 and TRO.11 were used in the assay.

### 4.6 B Cell Sorting of Env‐Specific Clones

For isolation of N26‐VRC35.02‐.04 and N26‐VRC36.02‐.03, Env‐specific memory B cells were isolated from cryopreserved PBMCs of donor N26 using previously described primers and methods [24]. Briefly, cells were thawed, viability‐stained, and labeled with antibodies against lineage markers (CD3, CD8, CD14) and B cell markers (CD19, IgM, IgG). Antigen‐specific cells were identified using fluorophore‐conjugated ST11 probes displaying V3 glycans. Single Env‐specific memory B cells (CD3^−^CD8^−^CD14^−^CD19^+^IgM^−^IgG^+^ST11^+^) were sorted into 96‐well plates containing lysis buffer using a FACSAria II cell sorter (BD Biosciences). Sorting data were analyzed using FlowJo software.

### 4.7 Single Cell RT‐PCR of Ig Genes

Immunoglobulin heavy‐ and light‐chain genes were amplified from single B cells or culture‐expanded B cells as previously described [24]. Briefly, cellular mRNA was reverse transcribed using random hexamers and SuperScript III reverse transcriptase. Variable regions of IgG heavy, κ, and λ chains were amplified for 50 cycles by PCR using gene‐specific primer sets, followed by 50 additional cycles with primers with annealing sites nested within those of the first amplification cycles. Amplified products were subsequently used for cloning and sequence analysis.

### 4.8 Biotinylation of mAbs

Monoclonal antibodies were biotinylated using EZ‐Link Sulfo‐NHS‐Biotin (Thermo Fisher Scientific) as described previously [24]. Briefly, antibodies were buffer‐exchanged into bicarbonate buffer, reacted with biotin reagent at an optimized molar ratio, and excess biotin was removed by desalting chromatography. Biotinylated antibodies were collected and stored in Tris‐buffered saline.

### 4.9 Binding Competition Assay With Cell Surface‐Expressed HIV‐1 Env

Binding competition experiments were performed using HEK293T cells (RRID: CVCL_0063) engineered to express strain TRO.11 HIV‐1 Env by transient transfection as described previously [24]. Transfected cells were harvested, stained with a viability dye, and incubated with serial dilutions of unlabeled antibodies in the presence of biotinylated reference antibodies. Following incubation and formaldehyde fixation, bound antibodies were detected using streptavidin‐PE and analyzed by flow cytometry (BD LSRII). Mean fluorescence intensity values were background‐subtracted and used to assess competitive binding.

### 4.10 Production of HIV‐1 Env‐Pseudotyped Viruses

HIV‐1 Env‐pseudotyped viruses were generated in HEK293T cells by co‐transfection of an Env‐expressing plasmid and an Env‐deficient backbone plasmid (pSG3ΔEnv), as previously described [24]. Briefly, supernatants containing pseudovirus were harvested, filtered, aliquoted, and stored at −80°C. Site‐directed mutagenesis was performed to introduce specific mutations into Env plasmids. Glycan knockouts were generated by mutating residues at the first position (Asn) or third position (Ser or Thr) of *N*‐linked glycosylation sequons.

### 4.11 TZM‐bl HIV‐1 Env‐Pseudotyped Virus Neutralization Assay

Neutralization assays were performed using TZM‐bl cells as described previously [24]. Briefly, serial dilutions of antibodies or serum samples were incubated with HIV‐1 pseudovirus prior to the addition of TZM‐bl cells. After incubation, infection levels were quantified by measuring luciferase activity. Neutralization was calculated relative to virus‐only controls, and inhibitory concentrations or titers (IC_50_/IC_80_ or ID_50_/ID_80_) were determined using nonlinear regression. High‐throughput 208‐virus neutralization assays were performed in 384‐well format using an automated liquid handling system as described previously [24].

### 4.12 Complex Assembly

Antibody heavy and light chain variable region DNA sequences were codon‐optimized and synthesized (Gene Universal Inc., Newark, DE). The heavy and light chain genes were subcloned into our pVRC8400‐based IgG heavy chain and light chain expression vectors respectively. Paired heavy/light chain constructs were transfected (Turbo293 transfection reagent, Speed BioSystems) into Expi293F cells for antibody production following manufacturer's protocol. Secreted antibodies in the culture medium were purified through Protein A (0.5 mL; GE Health Science) affinity columns. Protein A‐captured antibodies were eluted off the columns with a low‐pH IgG elution buffer (Pierce). Antibodies in low pH buffer were then dialyzed into phosphate‐buffered saline (PBS) and stored at −80°C.

For Fab fragment production, each heavy chain variable region was subcloned into pVRC8400 vector engineered to contain an HRV3C protease cleavage site in the hinge region. Following the same procedure as regular antibody production, the heavy chain HRV3C construct was paired with corresponding light chain construct and transfected into Expi293F cells. As described above, culture supernatants were harvested, clarified by centrifugation, and applied to Protein A affinity columns. Instead of low‐pH buffer elution, the HRV3C site containing antibodies on column were digested to release Fab fragments. The Fab was subsequently washed off the Protein A column and further purified by size‐exclusion chromatography using a Superose 6 Increase column (Cytiva) equilibrated in PBS.

For HIV‐1 Env trimer purification, BG505.T332N DS‐SOSIP.664 Env trimer was produced from a stable CHO‐DG44 cell line (RRID: CVCL_7180) and purified by non‐affinity chromatography methods (VRC Production Program) [27]. BG505.T332N DS‐SOSIP.664 Env trimer, B.X2278.C2.B6 Env trimer, B.CNE14 Env trimer, CD.3817.V2.C59 Env trimer, C.6785.V5.C14 Env trimer and glycan mutants Env trimers used for analysis were produced as described previously [28, 29] with minor modifications. Briefly, 150 µg of furin plasmid DNA and 600 µg of a construct encoding the N‐terminal single chain Fc tag, HRV3C cleavage site, and the BG505 DS‐SOSIP or individual glycan mutant Env trimers were co‐transfected into 1 liter of 293 FreeStyle cells using 2.25 mL of Turbo293 transfection reagent. After 6 days of incubation at 37°C, 120 rpm, and 9% CO_2_, the supernatants were harvested. Soluble Env trimers were captured using PBS‐equilibrated Protein A resin, rinsed with PBS, and cleaved from the column with HRV3C enzyme at 4°C overnight. The liberated Env trimers were then applied to a Superdex 200 16/600 gel filtration column. The fractions corresponding to Env trimers were pooled and subjected to negative selection with a V3 cocktail column [30]. The purified Env trimers were concentrated to 1 mg/mL in PBS containing 10% glycerol and kept in −80°C for long‐term storage.

Similarly, 1 mg of a construct encoding His‐tagged influenza hemagglutinin trimer [31] or 1 mg of a construct encoding His‐tagged Lassa GPC trimer [32] or 1 mg of construct encoding His‐tagged SARS‐CoV‐2 spike trimer [33] was transfected into 1 liter of Expi 293F cells at a density of 2.5 × 10^6^ cells/mL, using 3 mL of Turbo293 transfection reagent. After 6 days of incubation at 37°C, 120 rpm, and 9% CO_2_, the supernatants were harvested. His‐tagged trimers were captured using PBS‐equilibrated Ni‐NTA resin. After washing with 25 mm imidazole, Ni‐NTA resin‐captured trimers were eluted with 250 mm imidazole and then applied to a Superdex 200 16/600 gel filtration column. Fractions corresponding to the target trimers were pooled, concentrated to 1 mg/mL, and stored at −80°C for long‐term storage.

To prepare assembled complexes, VRC36 Fab and HIV‐1 BG505.T332N DS‐SOSIP.664 Env trimer were mixed at 8 to 1 molar ratio. 0.025% GA was added at a final total protein concentration of ∼0.05 mg/mL [34, 35, 36, 37]. After waiting for 10 min, the crosslinking reaction was quenched by addition of Tris‐HCl (pH 8.0) buffer to a final concentration of 40 mM. Similarly, VRC35 Fab was mixed with HIV‐1 BG505.T332N DS‐SOSIP.664 Env trimer at a 15:1 molar ratio, with influenza H3N2 Victoria 2011 hemagglutinin at an 8:1 ratio, with SARS‐CoV‐2 spike trimer at an 8:1 ratio, and with Lassa virus GPC trimer at an 8:1 ratio. All complexes were crosslinked prior to vitrification on cryo‐EM grids.

### 4.13 Electron Microscopy

For negative staining, VRC35 IgG, VRC35 Fab, VRC36 IgG, or VRC36 Fab was diluted with buffer composed of 10 mM HEPES, pH 7.0, and 150 mM NaCl to 0.01‐0.02 mg/mL. A 4.8 µL drop of the diluted sample was placed for 10–15 s on a glow‐discharged carbon‐coated copper grid. The drop was then removed with filter paper, and the grid was washed three times with drops of the same buffer, which was followed by negative stain with three drops of 0.75% uranyl formate. Data were collected using a 4k × 4k Ceta camera (Thermo‐Fisher Scientific) on a Talos F200C electron microscope (Thermo‐Fisher Scientific) operated at 200 kV. The nominal magnification was 57,000, which corresponded to a pixel size of 2.53 Å. RELION 4.0 was used for particle picking and 2D classification [38].

For cryo grids preparation, the assembled complexes were adjusted to have a final concentration of 0.005% (w/v) n‐Dodecyl β‐D‐maltoside (DDM) to prevent preferred orientation and aggregation during vitrification. Aliquots (4 µL) of samples, at a concentration of 0.5 mg/mL, were applied to glow‐discharged C‐flat Au grids (R1.2/1.3, 300 mesh) within the chamber of an FEI Vitrobot Mark IV (4°C and 100% humidity), followed by rapid vitrification in liquid ethane bath. VRC35‐BG505.T332N DS‐SOSIP.664 dataset, VRC35‐MEDI8852‐H3N2 Victoria 2011 HA dataset, VRC35‐SARS‐CoV‐2 LP.8.1.9 spike dataset, and VRC35‐LASV Josiah GPC dataset were acquired on a Titan Krios microscope operated at 300 kV. 20 eV energy filtered image stacks were recorded by a BioQuantum K3 camera (Gatan) with SerialEM [39] in the super‐resolution dose‐fractionated electron counting mode. A magnification of 105,000× was used, which rendered a final pixel size of 0.415 Å at object scale (super‐resolution). Each image stack consisted of 50 frames, collected at a defocus range of −0.7–−2.0 µm, with a total electron exposure of 54.5 e^−^/Å^2^. VRC36‐BG505.T332N DS‐SOSIP.664 dataset was acquired on a ThermoFisher Glacios microscope operated at 200 kV. Similarly, 20 eV energy filtered image stacks were recorded by a BioQuantum K3 camera (Gatan) with SerialEM [39] in the super‐resolution counting and movie mode. A magnification of 45,000× was used, yielding a final pixel size of 0.44 Å at the object scale (super‐resolution). Each movie was fractionated into 50 frames at a defocus range of −0.7–−2.0 µm, with a total electron exposure of 70.53 e^−^/Å^2^.

### 4.14 Image Processing

For VRC35‐BG505.T332N DS‐SOSIP.664 dataset, 11,020 movie stacks were imported into cryoSPARC 3.3 [40]. Motion correction was performed on each movie stack using Patch Motion Correction with the pixel size of 0.83 Å. CTF parameters were estimated using patch CTF, excluding micrographs with a CTF fitting resolution worse than 10 Å. Ultimately, a total of 10,358 qualified movie stacks were selected for image processing.

From these movie stacks, 3,272K particles were picked and extracted using a box size of 460. After one round of 2D classification, 1,299K particles were retained to generate an initial model via Ab‐Initio Reconstruction. Simultaneously, bad references were also generated from poor‐quality classes. Using these references, all selected particles were subjected to rounds of heterogenous refinement, resulting in 299K particles.

Given the symmetrical binding sites of VRC35 Fabs on the HIV‐1 Env trimer, non‐uniform refinement with C3 symmetry was applied, yielding a 3.39 Å resolution map. To select the particles displaying stronger VRC35 Fab local density, global 3D classification was performed, isolating 67K particles. To further enhance the local density of the VRC35 Fab region, C3 symmetry expansion was used to triple the particle count. This was followed by particle subtraction, re‐centered local refinement and focused 3D classification on the VRC35 Fab region. Two major conformational subsets for VRC35 Fabs were identified: one with two VRC35 Fabs visible (66,429 particles, 4.17 Å local map) and another with four VRC35 Fabs visible (66,136 particles, 4.20 Å local map).

Similar image processing workflows were applied to VRC35‐B.X2278.C2.B6, VRC35‐B.CNE14, VRC35‐CD.3817.v2.c59, and VRC35‐C.6785.v5.c14 datasets. The resulting 2D class averages confirmed a universal stoichiometry of 12 VRC35 Fabs bound per HIV‐1 Env trimer across diverse strains.

For VRC35‐MEDI8852‐H3N2 Victoria 2011 HA dataset, 12,065 qualified movie stacks were selected for image processing. Around 3,775K particles were auto‐picked, and screened by a cascade of 2D and global 3D classifications, resulting in a dataset of 2,006K particles for further processing. Similar to the VRC35‐BG505.T332N DS‐SOSIP.664 dataset, particles were initially refined with C3 symmetry imposed. Since weak VRC35 Fabs density was observed when the entire map was displayed at 1 σ, particle subtraction and 3D classification were applied to the VRC35‐HA head region to enhance VRC35 Fab density. This process isolated 1,325K particles with stronger VRC35 Fab density. Given the symmetrical VRC35 binding sites on HA head, C3 symmetry expansion was employed to triple the particle count. Subsequently, particle subtraction and multiple rounds of 3D classification further isolated 123K particles for dimeric VRC35 Fabs local refinement.

For VRC35‐SARS‐CoV‐2 LP.8.1.9 spike dataset, 8,575 qualified movie stacks were selected for image processing. Approximately 1,500K particles were auto‐picked, and screened by a cascade of 2D and global 3D classifications, yielding a subset of 511K particles for further processing. Although the global map reached an overall resolution of 3.08 Å, the density corresponding to the VRC35 Fab density was weak at a contour level of 3 σ, but became relatively enhanced at 1 σ. Focused particle subtraction and local 3D classification were applied to classify VRC35 region. 75.2% of the particles (384K) were discarded and 24.8% of theparticles (127K) were retained for further refinement. The selected particles were re‐centered and re‐extracted for C1 homogenous refinement, with VRC35 Fab density significantly improved. Subsequent rounds of defocus refinement, global CTF refinement and Ewald sphere correction produced a final reconstruction at 3.68 Å overall resolution, with a local resolution of approximately 6 Å in the VRC35 Fab region.

For VRC35‐LASV Josiah GPC dataset, 8,206 qualified movie stacks were selected for image processing. 1,046K particles were selected for 2D classification. The 2D class averages displayed several Fab‐binding states, including unbound trimers, complexes with one Fab bound, classes with one strong Fab density accompanied by one or two weaker densities. Subsequent ab‐initio reconstruction and heterogenous refinement revealed that ∼19% of the particles (197K) contained one Fab bound, whereas ∼81% of the particles (832K) were reconstructed without Fab density. Further global 3D classification yielded a final subset of 62K particles, reconstructed to an overall resolution of ∼7.2 Å. Distinct density was observed between the LASV GPC trimer and the VRC35 Fab, consistent with a glycan moiety extending from the outer surface of the GPC trimer.

For VRC36‐BG505.T332N DS‐SOSIP.664 dataset, 4,125 qualified movie stacks were selected for image processing. Initial processing included 2,840 micrographs, from which 515K particles were auto‐picked for multiple rounds of 2D and global 3D classification. This process isolated 130K particles for an initial Non‐uniform Refinement with C1 symmetry.

Unexpectedly, the initial C1 reconstruction displayed heterogeneous Fab occupancy, with stronger Fab densities visible at higher contour levels and additional weaker densities emerging at lower contour levels. This observation suggested the presence of multiple Fab‐binding states and prompted further rounds of 3D classification. Further rounds of Ab‐initio Reconstruction and Heterogeneous Refinement identified two major particle populations. Class I (75K particles, 57.7%) displayed a single dimeric VRC36 Fab assembly per Env trimer, corresponding to two bound Fabs. Class II (53K particles, 40.8%) contained additional Fab densities consistent with multiple occupied binding sites. These classifications established the presence of distinct Fab occupancy states prior to subsequent local refinement.

Simultaneously, 2D classification was performed based on the initial model, producing 2D references for template‐based picking across the entire dataset. Subsequently, 1,092K particles were auto‐picked from 4,125 micrographs. After additional rounds of 2D and 3D classification, 111K particles were selected for final Non‐uniform Refinement. Given the symmetrical binding sites of VRC36 Fabs on the HIV‐1 Env trimer, Non‐uniform Refinement with C3 symmetry was applied, resulting in a 3.73 Å resolution map. After CTF refinement and Bayesian polishing, the final density map achieved an overall resolution of 3.59 Å. Similar to VRC35‐BG505.T332N DS‐SOSIP.664 dataset, C3 symmetry expansion was applied. Subtraction of the dimeric VRC36 Fab followed by focused 3D classification isolated 76% of the particles (253K) for the local refinement, yielding a 3.62 Å resolution local map.

The resolution estimation was determined using gold‐standard FSC at a threshold of 0.143. The local resolution maps were generated using CryoSPARC 3.3 [40]. Structural analysis was performed using UCSF Chimera 1.11.2 [41] and Pymol (http://pymol.org).

### 4.15 Atomic Model Building and Refinement

For structural analysis, an initial model of the antibody Fab fragment was predicted using AlphaFold2. The coordinates of the BG505.T332N DS‐SOSIP.664 (PDB: 4ZMJ) and H3N2 Vic11 (PDB: 5JW4) were used as initial model for fitting the cryo‐EM map. Glycosylation of Man_8_ in the VRC35‐HA‐MEDI8852 complex was performed by CHARMM‐GUI Glycan Reader & Modeler [42, 43, 44, 45]. Individual domains were first docked into the density map manually in UCSF Chimera, followed by iterative model adjustment in Coot 0.9.5 [46]. Subsequent real‐space refinement was carried out using Phenix.real_space_refine [47] with restraints applied to secondary structure and overall geometry. Model quality was assessed using MolProbity 1.19.2 [48]. Interfaces were analyzed with PISA [49]. Structural visualization and figure preparation were performed in PyMOL (Schrodinger; http://www.pymol.org) and UCSF ChimeraX [50]. Detailed statistics for the final models are provided in the tables.

### 4.16 Isothermal Titration Calorimetry (ITC)

Thermodynamic binding parameters were determined at 25°C using a MicroCal VP‐ITC (Malvern Panalytical, Northampton, MA, USA). All proteins were dissolved in and exhaustively dialyzed against PBS, pH 7.4 prior to the experiments. The solution of Man_9_ was prepared by dissolving the lyophilized Man_9_ in the dialysate. For the experiments with Man_9_, the calorimetric cell was filled with ∼2.5 µM IgG (expressed per antigen binding sites) and a solution of Man_9_, prepared at a concentration of 500 µM, was added stepwise in 10 µL aliquots. For the remaining experiments, the calorimetric cell was filled with HIV‐1‐Env trimer (DS‐SOSIP), influenza‐hemagglutinin head, or SARS‐CoV‐2 spike, at 0.3‐1 µM and stepwise additions were made of IgG or Fab in 8–10 µL aliquots. The concentration of antigen binding sites or Fab in the syringe was ∼25 µM for VRC01 IgG and Fab, ∼45 µM for VRC36 IgG and Fab, and 70 µM for VRC35 IgG and Fab. The injections were spaced at 500 s intervals, and the experiments were carried out at 300 rpm stirring speed and low feedback. For each experiment, the exact concentrations of the proteins were determined from the protein absorbance at 280 nm. The concentration of Man_9_ was based on dry weight.

The heat accompanying the injections was obtained by integration of the calorimetric heat flow, dQ/dt, and the heat associated with binding was obtained after subtraction of the heat of dilution. The association constant, *K_a_* (the dissociation constant, *K_D_* = 1/*K_a_*), the enthalpy change, *ΔH*, and the stoichiometry, *N*, were obtained directly by nonlinear regression of the data to a single‐site binding polynomial. The change in Gibbs energy, *ΔG*, was calculated from the binding affinity through *ΔG* = ‐*RT*ln *K_a_*, where *R* is the gas constant (1.987 cal/(K × mol) and *T* is the absolute temperature in kelvin. The entropy contribution to Gibbs energy, *‐TΔS*, was calculated using *ΔG* = *ΔH ‐TΔS*.

### 4.17 Analytical Ultracentrifugation Sedimentation Velocity (AUC)

AUC measurements were performed at 25°C using a Beckman Optima XL‐A/I analytical ultracentrifuge from Beckman‐Coulter (Palo Alto, CA, USA), using interference detection at 660 nm in two‐channel cells with 12 mm path length and sapphire windows. The rotor speed was 20,000 rpm, and scans were taken every 60 s. All samples were dialyzed into PBS at +8°C overnight, and the protein concentrations were for BG505 and VRC01: 2.2 mM BG505 and 11 mM VRC01 Fab, for BG505 and VRC35 Fab: 2.1 mM BG505 and 55 mM VRC35 Fab, and for BG505 and VRC36: 2.1 mm BG505 and 27 mM VRC36 Fab. Buffer density, viscosity and protein v‐bar were calculated using the program SednTerp (Alliance Protein Laboratories, Corte Canyon, Thousand Oaks, CA, USA), data were analyzed with Sedfit software (https://sedfitsedphat.nibib.nih.gov/software/default.aspx)

### 4.18 Sedimentation Equilibrium Measurements

Sedimentation equilibrium experiments were conducted using a Beckman XL‐A/I analytical ultracentrifuge (Beckman‐Coulter, Palo Alto, CA, USA). Six‐channel centerpieces with straight walls, a 12 mm optical path length, and sapphire windows were employed. Prior to analysis, all protein samples were dialyzed overnight and subsequently diluted in PBS (pH 7.4). Final concentrations of 2.0, 1.3, and 0.67 mg/mL were prepared in channels A, B, and C, respectively, with the corresponding buffer serving as a reference. Each sample was analyzed at four rotor speeds: 15,000, 19,000, 23,000, and 27,000 rpm. The lowest speed was maintained for 20 h, followed by four scans collected at 1‐h intervals. The second‐lowest speed was held for 10 h, after which four scans were acquired in the same manner. The two higher speeds were conducted under identical conditions as the second‐lowest speed. All measurements were performed at 25°C, and interference optics were used for detection at 675 nm. Solvent densities were calculated using SednTerp (Alliance Protein Laboratories, Thousand Oaks, CA, USA), and the partial specific volume (v̄) of the protein was set to 0.73 mL/g. Apparent molecular weights were determined by global fitting of all relevant datasets using SedPhat (National Institutes of Health, USA).

### 4.19 High‐Throughput Affinity Measurements Using Carterra

A medium‐density SAHC30M sensor chip was conditioned by sequential 1 min injections of 50 mm NaOH, 1 M NaCl, and 10 mm glycine (pH 2.0). Antigens were diluted to 1 µg/mL in running buffer consisting of 10 mM HEPES (pH 7.4), 150 mM NaCl, 3 mM EDTA, and 0.01% (v/v) Tween‐20 (HBSTE), supplemented with 0.5 mg/mL BSA, and captured on the chip surface for 5 min. VRC35 and VRC36 Fab samples were prepared at 2,000 nM and serially diluted threefold in HBSTE containing 0.5 mg/mL BSA before injection. Each concentration was injected with a 5 min association phase followed by a 10 min dissociation phase. IgG samples were processed in the same manner, except the initial concentration for the dilution series was 1,000 nM. Injections were performed in order of increasing analyte concentration. To correct for background and nonspecific binding, responses from local reference spots were subtracted from active spot signals, and buffer blank responses were further subtracted to obtain double‐referenced data. The resulting datasets were analyzed using a 1:1 Langmuir binding model in Carterra's Kinetic Inspection Tool.

### 4.20 Glycan Microarray Assay

An N‐glycan microarray slide was used to determine N‐glycan binding characteristics of antibodies. Each antibody sample was diluted in glycan array assay buffer (GAAB) to a concentration of 20 µg/mL. Anti‐Human IgG (Fc) Cy3 was then added to each mixture at a concentration of 10 µg/mL. The precomplex antibody mixtures were then allowed to incubate on ice for 1 h. After 1 h, each mixture was further diluted in a serial dilution to concentrations of 5, 1.25, and 0.3125 µg/mL. The N‐glycan array slide was first treated with glycan array blocking buffer (GABB) for 30 min. After blocking, the slide was washed three times with GAAB prior to adding the antibody samples to the slide. After addition to the slide, the antibody samples were incubated for 1 h at room temperature before a final wash and scan of the slide. After the assay, the microarray was scanned at 532 nm using low laser intensity (1 PMT). Subsequently, Innopsys's Mapix software was employed to further analyze the array scan. The binding signals were determined by subtracting background signals and signals from negative control spots.

### 4.21 N‐Glycosylation Analysis by LC‐MS/MS

The LC‐MS/MS data acquisition was performed on an Orbitrap Q Exactive HF mass spectrometer connected to Waters Acquity UPLC H‐Class. For the protein digestion, about 50 µg of each protein sample was taken and diluted to 50 µL with 50 mM Ammonium bicarbonate buffer (ABC). To the samples, 1.5 µL of dithiothreitol (500 mM) was added and incubated at 60°C for 20 min. Further, 4.5 µL of iodoacetamide (500 mM) was added to the samples and kept in dark at room temperature for 15 min. The samples were desalted by passing through 10 kDa ultra centrifugal filters (Amicon) and the filters were washed twice with 400 µL of ABC. After recovering the proteins from the filter, the volume of the samples was adjusted to 50 µL with ABC. For the protease digestion, 25 µg of desalted samples were taken and 1.25 µL of trypsin was added (1 µg/µL) followed by 1.25 µL of elastase (1 µg/µL). The samples were incubated at 37°C for 12 hrs. After the digestion the samples were transferred to a total recovery LC‐vial and analyzed by LC‐MS/MS.

The LC‐MS/MS data files were processed by Byos software for glycosylation profile identification and quantification. The data files were searched against H3N2 fasta sequence using Byos PTM identification and quantification module. Trypsin and elastase cleavage sites (RK and AGSVIL, respectively) with semi‐specific cleavage option was selected for the search. 5 ppm precursor and 10 ppm fragment mass tolerance were chosen. Carbamidomethylation of cysteine as fixed modification, and oxidation of methionine, deamidation of asparagine and common human 182 N‐glycan database as variable modifications were selected for the search. Automatic score cut, fast XIC (extracted ion chromatogram), and 1% FDR (false discovery rate) were also enabled. During the Byos software search, the glycosylation profile at each site was identified and quantified by extracting the peak area of glycopeptides. Further, the assigned MS spectra were validated manually to eliminate false assignments.

### 4.22 Antibody Autoreactivity Assessment

Antibody autoreactivity was evaluated using HEp‐2 cell staining and anti‐cardiolipin ELISA kits (ZEUS Scientific and Inova Diagnostics, respectively) following the manufacturers’ instructions. For HEp‐2 assays, antibodies were tested at two concentrations, and fluorescence images were acquired by microscopy under identical exposure settings. Reactivity scores were assigned by comparing fluorescence intensity to a panel of control antibodies with predefined reference levels. Antibodies with scores above a defined threshold were classified as autoreactive, while lower scores were considered weak or non‐reactive. For anti‐cardiolipin ELISA, antibodies were tested in serial dilutions, and phospholipid reactivity was quantified based on standard curves. Responses were categorized into non‐reactive, low‐positive, or high‐positive according to established GPL unit ranges.

### 4.23 Influenza Reporter Virus Microneutralization

Replication‐restricted influenza reporter viruses (R3ΔPB1) representing multiple strains were generated as previously described [51]. Briefly, the PB1 segment was replaced with a fluorescent reporter gene, and viruses were rescued using reverse genetics and propagated in PB1‐complementing cell lines. Virus stocks were titrated prior to use by infecting MDCK‐SIAT1 cells (RRID: CVCL_Z936) engineered to complement PB1 and quantifying fluorescent signal using automated imaging. For neutralization assays, serial dilutions of antibodies were incubated with reporter virus before addition of target cells. Following incubation, infection levels were determined by fluorescence readout. Neutralization percentages were calculated relative to virus‐only and cell‐only controls, and dose response curves were fitted using nonlinear regression to determine IC_50_ values. The reporter virus panel was used to evaluate VRC35 neutralization across a broad collection of H3N2 influenza strains spanning multiple decades of antigenic evolution. Because the available virus panel differed from that used in conventional microneutralization assays, the two assay formats were considered complementary approaches for assessing influenza‐neutralizing activity rather than direct comparisons of individual viral isolates.

### 4.24 Influenza Virus Propagation and Reverse Genetics

A/H3N2 and A/H1N1 influenza viruses used in vitro microneutralization assays were grown in‐house in specific pathogen free (SPF) eggs, harvested, purified, and titered [52]. The pPolI plasmids encoding HA (the polybasic amino acid cleavage site was modified to be LPAI strain) and NA derived from A/Texas/37/2024, the pPolI PB2(120)GFP(120) plasmid [53], and the five pPolI plasmids encoding the remaining segments of high‐yield A/Puerto Rico/8/34 virus were used [54]. The pPolI PB2(120)GFP(120) plasmid contains the A/Puerto Rico/8/34‐derived 3′ PB2 non‐coding region, 120 nt that correspond to the PB2 coding sequence at the 3′ end of the vRNA followed by the GFP coding sequence, 120 nt that correspond to the PB2 coding sequence at the 5′ end of the vRNA and finally the 5′ PB2 non‐coding region. These eight pPolI plasmids along with protein expression plasmids for PB2, PB1, PA, and NP derived from A/Puerto Rico/8/34 were transfected into 293T cells by use of Lipofectamine 3000 Transfection Reagent (Invitrogen) according to the manufacturer's instructions. At 48 hrs post‐transfection, the supernatants were harvested and inoculated into AX4 cells [55], derived from MDCK cells and transfected with the cDNA of human 2,6‐sialyltransferase, stably expressing the PB2 protein derived from A/Puerto Rico/8/34.

### 4.25 In Vitro Influenza Microneutralization Assay

The neutralization potency of the monoclonal antibodies was assessed by in vitro microneutralization assay. For in vitro microneutralization assay, the serially diluted antibodies were mixed with a certain amount of influenza virus and incubated prior to addition to AX4 cells stably expressing PB2 protein. The cells were incubated at 37°C with 5% CO_2_ for 24 hrs. Cells were treated with ice‐cold acetone to inactivate virus and fix/permeabilize AX4 cells. Fixed cells were subsequently washed with PBS, blocked with BSA, incubated with detection antibody, and developed with colorimetric substrate to visualize neutralization potency. IC_50_ values as the inhibitory concentrations of antibodies needed to lower by 50% the viral infection were calculated. Conventional microneutralization assays were performed using representative contemporary H3N2 strains and served as an independent validation of the influenza‐neutralizing activity observed in the reporter virus assay.

### 4.26 Flow Cytometry‐Based SARS‐CoV‐2 Virus Neutralization

A total of 30,000 Vero‐TMPRSS2 cells (JCRB1819, RRID: CVCL_YQ48) were seeded into each well of a 96‐well plate. The following day, 50 µL of 10‐fold serially diluted monoclonal antibodies (mAbs) were incubated with 10,000 PFU^*^ of SARS‐CoV‐2 isolates WA.1 or JN.1 in 50 µL for 30 min at 37°C. The virus–mAb mixtures were then added to the Vero‐TMPRSS2 cells and incubated overnight. The next day, cells were washed with PBS and detached using TrypLE. Cells were subsequently fixed with 10% neutral buffered formalin for 30 min before being transferred out of the BSL‐3 facility for immunostaining. Next, cells in polypropylene round‐bottom 96‐well plates were pelleted (500 RCF, 5 min, 4°C), supernatants aspirated, and fixed/permeabilized with 40 µL Fixation/Permeabilization buffer (Invitrogen eBioscience Foxp3/Transcription Factor kit) for 30 min at 4°C. Cells were washed with 200 µL Permeabilization buffer, pelleted (500 RCF, 5 min, 4°C), and stained with 30 µL anti–SARS‐CoV‐2 nucleoprotein mAb N18 directly conjugated to Alexa Fluor 647 (1:200) for 1 h on ice. After adding 200 µL Permeabilization/Wash buffer and centrifugation (500 RCF, 5 min, 4°C), cells were resuspended in 250 µL PBS containing 0.1% FBS and 0.1 mMm EDTA. Samples were acquired on a BD LSRFortessa X‐20 flow cytometer, including no‐virus and no‐antibody controls in each run. We normalized the frequencies of NP‐positive cells to the non–antibody‐treated samples or to the plateau observed for at least the two highest Ab dilutions (set to 100%) and fitted nonlinear regression curves using the dose‐response inhibition model to derive neutralization titers in GraphPad Prism 9.

### 4.27 SARS‐CoV‐2 Pseudovirus Neutralization

SARS‐CoV‐2 pseudoviruses were produced by co‐transfecting of packing plasmid pCMVdR8.2, transducing plasmid pHR’ CMV‐Luc and codon‐optimized spike plasmids from WA1, BA.2.86, JN.1, KP.3, KP.3.1.1, LF.7.2.1, LP.8.1.9, NB.1.8.1 and BA.3.2 into 293T/17 [HEK 293T/17] cells (ATCC Catalogue CRL‐11268, RRID: CVCL_1926). Cells were transfected overnight using Lipofectamine 3000 transfection reagent (ThermoFisher Scientific Catalogue L3000‐001) and then replaced with fresh medium. 48 h later, supernatants were harvested, filtered through a 0.45 µm syringe filter, aliquoted and frozen at ‐80°C before use. The neutralization assays were carried out as follows: serial dilutions of the monoclonal antibodies were mixed with titrated SARS‐CoV‐2 pseudoviruses and incubated for 45 min at 37°C in 96‐well white/black Isoplates (Revvity/PerkinElmer Catalogue 6005060) and 293‐TMPRSS2‐ACE2 cells at 10,000 cells per well were added. After 72 h, cells were lysed and luciferase activity was measured with MicroBeta2 MicroPlate Counter (Revvity/PerkinElmer, Waltham, MA). Percent neutralization and neutralization IC_50_ and IC_80_ values were calculated by Excel and Prism GraphPad 9.0.

### 4.28 LASV Pseudovirus Production

HEK293T cells were seeded 24 h prior to transfection to achieve 80%–90% confluency. Cells were transfected with a plasmid encoding the appropriate LASV GPC using Lipofectamine 3000 (Thermo Fisher Scientific) according to the manufacturer's instructions. After overnight incubation at 37°C with 5% CO_2_, the medium was replaced and cells were infected with luciferase‐expressing VSV‐G pseudovirus at a MOI of 3. Following 1 h of infection at 37°C, cells were washed twice with 1X DPBS. Fresh culture medium (supplemented with fetal bovine serum and penicillin‐streptomycin) was added, and cells were further incubated for 24 h. The supernatant was collected, clarified by centrifugation at 2000 rpm for 10 min, and filtered through 0.45 µm polyethersulfone (PES) syringe filters. To neutralize residual VSV‐G virions, I1‐hybridoma supernatant was added at 20% of the collected volume. Clarified pseudovirus were aliquoted and stored at −80°C for subsequent use.

### 4.29 LASV Pseudovirus Neutralization

Antibodies were serially diluted 5‐fold in triplicate and incubated with pseudovirus in 37°C with 5% CO_2_ for 1 h. Vero‐E6 cells (ATCC CRL‐1586, RRID: CVCL_0574) were added at 3 × 10^4^ cells per well and incubated for 24 h under the same conditions. Neutralization activity was quantified by measuring luciferase expression using the Firefly Luciferase Assay System (Promega). The 50% inhibitory concentration (IC_50_) was defined as the antibody concentration that reduced relative luminescence units (RLU) by 50% compared to virus control wells (pseudovirus + cells) after subtracting background signal from cell control wells. All calculations were completed with nonlinear regression analysis in GraphPad Prism 10.

## Author Contributions

J.C. conceived and designed the study. J.C., E.M.C., A.M., M.S.S., N.S.L., R.A.G., L.W., I.K., S.C., A.Y., T.B., A.S., P.T., Y.T., H.L., N.C.M., A.B.L., A.J.M., Z.C.L., J.E.B., I.R.F., M.L., N.L., C.L., R.S.R., C.S., I.T.T., D.J.V.W., D.W., L.W., G.A., M.C., H.D., M.J.E., T.L., B.C.L., M.K.L., K.M., J.S.M., S.O., L.O., S.P., D.P., Q.Q., S.R., M.S., S.S., A.S., A.R.S., B.Z., Q.Z., M.C., J.G.G., Y.G., R.K.H., Y.H., R.A.K., Q.P.L., J.R.M., R.R., L.S., Z.S., D.D.H., P.C.W., J.W.Y., T.C.P., M.K., N.A.D.R., P.D.K., and T.Z. performed experiments, analyzed data, and prepared figures. J.C. wrote the original draft of the manuscript. N.A.D.R., P.D.K., and T.Z. reviewed and edited the manuscript. All authors reviewed and approved the final version of the manuscript and assume responsibility for its content, including the accuracy of the data and the fidelity of the statistical analyses.

## Conflicts of Interest

The authors declare no conflicts of interest.

## Supporting information




**Supporting File**: advs77172‐sup‐0001‐SuppMat.pdf.

## Data Availability

Plasmids generated in this study are available upon request. Heavy and light chain sequences of antibodies VRC35.01‐.03 and VRC36.01‐.05 are available in GenBank under accession numbers PZ673057‐PZ673058 and PZ698527‐PZ698540. The antibody neutralization data on the 208‐pseudovirus panel is available in CATNAP (https://www.hiv.lanl.gov/components/sequence/HIV/neutralization/). Cryo‐EM structures of six VRC35 Fabs bound to influenza H3N2 Victoria 2011 hemagglutinin and six VRC36 Fabs bound to HIV‐1 BG505.T332N DS‐SOSIP.664 Env trimer were deposited in the Protein Data Bank (https://www.rcsb.org) under IDs 9OM5 and 9NPM, respectively. Cryo‐EM maps were deposited in the Electron Microscopy Data Bank (https://www.ebi.ac.uk/emdb/) as follows. For VRC35–HIV‐1 Env complexes: global map of twelve VRC35 Fabs (EMD‐48131), local map of four VRC35 Fabs (EMD‐48133), and local map of two VRC35 Fabs (EMD‐48134). For VRC35–influenza HA complexes: composite map of six VRC35 Fabs and three MEDI8852 Fabs bound to H3N2 Victoria 2011 hemagglutinin (EMD‐70607), global map of six VRC35 Fabs and three MEDI8852 Fabs (EMD‐49964), local map of six VRC35 Fabs bound to the hemagglutinin head (EMD‐48426), and local map of dimeric VRC35 Fabs bound to glycans N126, N165, and N246 on the hemagglutinin head (EMD‐48427). For VRC35 bound to SARS‐CoV‐2 spike: cryo‐EM map of VRC35 Fab bound to SARS‐CoV‐2 LP.8.1.9 spike (EMD‐74798), and local density map of VRC35 Fab bound to spike glycans (EMD‐74801). For VRC35 bound to LASV GPC: cryo‐EM map of VRC35 Fab bound to LASV Josiah GPC (EMD‐74843). For VRC36–HIV‐1 Env complexes: composite map of six VRC36 Fabs (EMD‐49628), global map of six VRC36 Fabs (EMD‐49633), global map of four VRC36 Fabs (EMD‐47895), global map of two VRC36 Fabs (EMD‐47893), and local map of dimeric VRC36 Fabs (EMD‐47885).
