# Supplementary material for: Canonical Antibodies Adopt Distinct Binding Modes to Recognize Viral Glycan Shields
Source: Adv Sci (Weinh). 2026 Aug 14;13(61):e77172. doi: 10.1002/advs.77172 (PMC13474179; doi:10.1002/advs.77172)
Supplement: Supplementary file 1 [file ADVS-13-e77172-s001.pdf]

Canonical Antibodies Adopt Distinct Binding Modes to Recognize Viral Glycan Shields

Jiaxuan Cheng[1], Evan M. Cale[1], Amirabbas Maghsoudi[1], Matthew S. Sutton[1], Nancy S. Longo[1], Rebecca A. Gillespie[1], Lingshu Wang[1], Ivan Kosik[2], Sue Chong[3,4], Atsuhiro Yasuhara[5], Tatsiana Bylund[1], Arne Schön[6], Prabhanshu Tripathi[1], Yaroslav Tsybovsky[7], Haotian Lei[8], Nicholas C. Morano[3,4], Allison B. Lupatkin[9], Abraham J. Morton[9], Zabrina C. Lang[9], Jordan E. Becker[3,4], Isabella R. Frascilla[1], Myungjin Lee[1], Ning Li[7], Cuiping Liu[1], Ryan S. Roark[3,4], Chen-Hsiang Shen[1], I-Ting Teng[1], David J. Van Wazer[1], Danyi Wang[1], Lingyuan Wu[1], Goran Ahlsen[3,4], Mike Castro[1], Haijuan Du[1], Michael J. Ernandes[1], Tiansheng Li[2], Bob C. Lin[1], Mark K. Louder[1]§, Krisha McKee[1], Jonah S. Merriam[1], Sijy O'Dell[1], Li Ou[1], Sergei Pletnev[1], David Prikryl[2], Qi Qiu[1], Sarah Rubin[1], Mallika Sastry[1], Stephen Schmidt[1], Asif Shajahan[1], Andrea R. Shiakolas[1], Sanjay Srivatsan[1], Baoshan Zhang[1], Qiong Zhou[1], Mark Connors[10], Jason G. Gall[1], Yicheng Guo[3,4], Rick K. Huang[9], Yaoxing Huang[3,4], Richard A. Koup[1], Q. Paula Lei[1], John R. Mascola[1], Reda Rawi[1], Leonid Serebryannyy[1], Lawrence Shapiro[3,4], Zizhang Sheng[3,4], David D. Ho[3,4], Patrick C. Wilson[5], Jonathan W. Yewdell[2], Theodore C. Pierson[1], Masaru Kanekiyo[1], Nicole A. Doria-Rose[1*], Peter D. Kwong[3,4*], and Tongqing Zhou[1*]

[1] Vaccine Research Center, National Institute of Allergy and Infectious Diseases, National Institutes of Health, Bethesda, MD 20892, USA
[2] Cellular Biology Section, laboratory of Viral diseases, National institute of Allergy and infectious diseases, Bethesda, MD 20892, USA
[3] Department of Biochemistry and Molecular Biophysics and Zuckerman Mind Brain Behavior Institute, Columbia University, New York, NY 10027, USA
[4] Aaron Diamond AIDS Research Center, Columbia University Vagelos College of Physicians and Surgeons, New York, NY 10032, USA
[5] Drukier Institute for Children's Health, Department of Pediatrics, Weill Cornell Medicine, New York, NY 10021, USA
[6] Department of Biology, Johns Hopkins University, Baltimore, Maryland, USA
[7] Vaccine Research Center Electron Microscopy Unit, Cancer Research Technology Program, Frederick National Laboratory for Cancer Research sponsored by the National Cancer Institute, Frederick, MD 21702, USA
[8] Research Technologies Branch, National Institute of Allergy and Infectious Diseases, National Institutes of Health, Bethesda, MD 20892, USA
[9] Laboratory of Cell Biology, National Cancer Institute, National Institutes of Health, Bethesda, MD 20892, USA
[10] Laboratory of Immunoregulation, National Institute of Allergy and Infectious Diseases, National Institutes of Health, Bethesda, MD, 20892, USA
§ Deceased

*Correspondence: nicole.doriarose@nih.gov (N.D.R.); pdk3@cumc.columbia.edu (P.D.K.); tzhou@nih.gov (T.Z.)

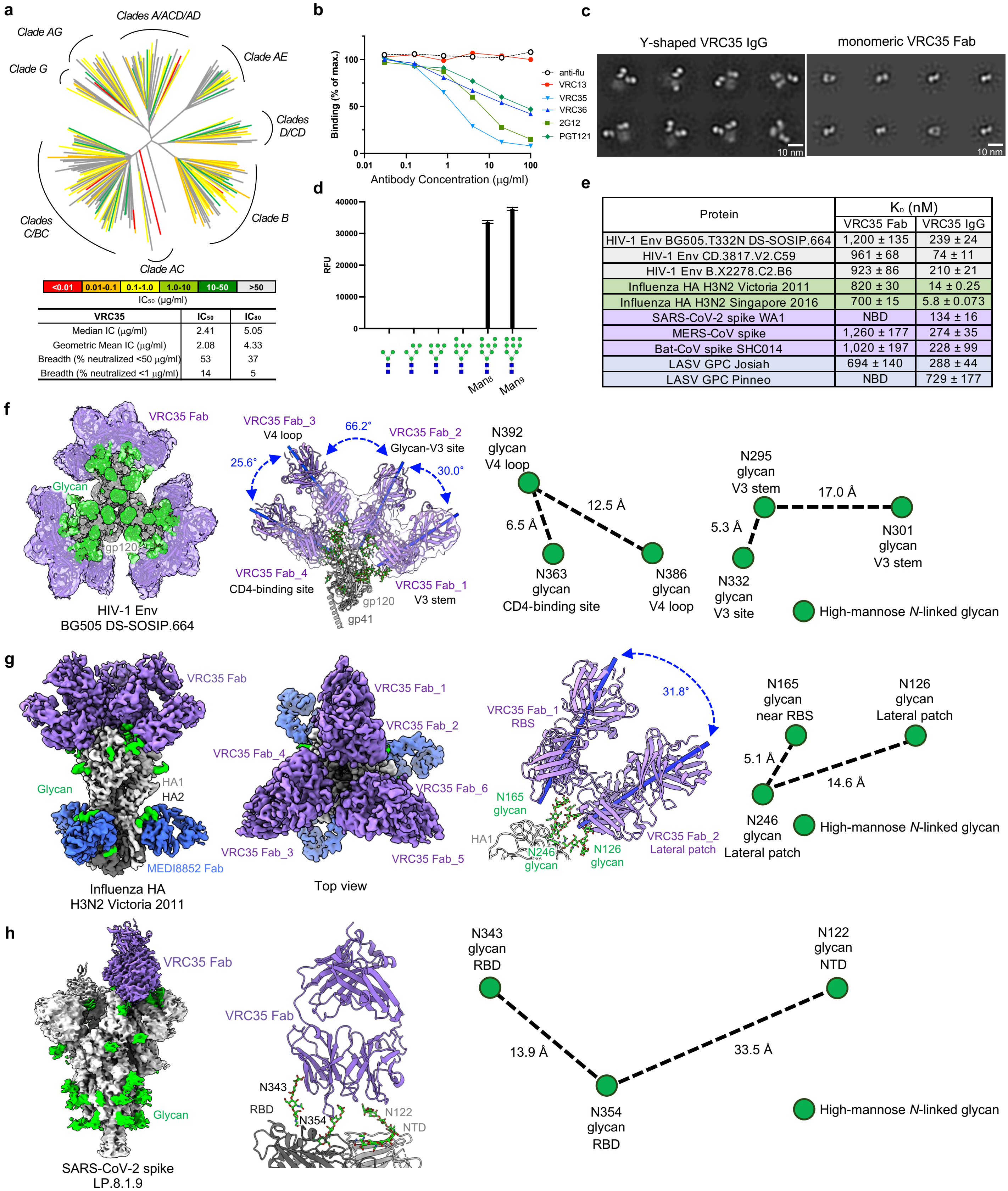

VRC35	IC_{50}	IC_{80}
Median IC (µg/ml)	2.41	5.05
Geometric Mean IC (µg/ml)	2.08	4.33
Breadth (% neutralized <50 µg/ml)	53	37
Breadth (% neutralized <1 µg/ml)	14	5

Protein	K_D (nM)	
	VRC35 Fab	VRC35 IgG
HIV-1 Env BG505.T332N DS-SOSIP.664	1,200 ± 135	239 ± 24
HIV-1 Env CD.3817.V2.C59	961 ± 68	74 ± 11
HIV-1 Env B.X2278.C2.B6	923 ± 86	210 ± 21
Influenza HA H3N2 Victoria 2011	820 ± 30	14 ± 0.25
Influenza HA H3N2 Singapore 2016	700 ± 15	5.8 ± 0.073
SARS-CoV-2 spike WA1	NBD	134 ± 16
MERS-CoV spike	1,260 ± 177	274 ± 35
Bat-CoV spike SHC014	1,020 ± 197	228 ± 99
LASV GPC Josiah	694 ± 140	288 ± 44
LASV GPC Pinneo	NBD	729 ± 177

Fig. 1 VRC35 adapted to distinct viral glycan landscapes. a Dendrogram of the 208-strain virus panel colored by VRC35 IC_{50} titers. **b** VRC35 binding competition assay. Lines indicate binding of labeled VRC35 to TRO.11-Env trimer-expressing 293T cells in the presence of serially diluted unlabeled competitor antibodies as measured by flow cytometry. **c** Negative-stain EM 2D class averages showed canonical Y-shaped VRC35 IgG and monomeric Fab. **d** Binding of VRC35 IgG to Man_8 and Man_9 glycans on a high-density microarray. Error bars indicated standard deviation. **e** SPR analysis of VRC35 Fab and IgG binding to HIV-1 Env, influenza HA, coronavirus spikes, and LASV GPC. **f** Cryo-EM structure of HIV-1 BG505.T332N DS-SOSIP.664 Env bound by twelve VRC35 Fabs. Surface representations (left) and cartoon representations (center) illustrate four Fab binding sites per Env protomer, targeting the V3 stem, glycan-V3 site, V4 loop, and CD4-binding site. Dimeric Fabs exhibited rotational angles of ~25°-30°. The glycan matrix (right) shows the Cα-Cα distances between *N*-linked glycans. **g** Cryo-EM structure of influenza HA (H3N2 Victoria 2011) bound by six VRC35 Fabs and three MEDI8852 Fabs. Surface representations (left and center) show side and top views of the complex, whereas the cartoon representation (right) highlights the dimeric VRC35 Fab assembly. VRC35 Fab_1 was positioned adjacent to the receptor-binding site (RBS) and engaged glycan N165, whereas VRC35 Fab_2 was located near the lateral patch and engaged glycans N126 and N246, forming a dimer with a 31.8° rotational angle. The glycan matrix (far right) shows the Cα-Cα distances among N126, N165, and N246. **h** Cryo-EM structure of SARS-CoV-2 spike (LP.8.1.9) bound by a single VRC35 Fab. The surface representation (left) shows the overall Fab-bound spike complex, whereas the cartoon representation (center) highlights the VRC35 interaction with glycans N343 and N354 on the receptor-binding domain (RBD) and glycan N122 on the N-terminal domain (NTD) of an RBD-down protomer. The glycan matrix (right) shows the Cα-Cα distances among N343, N354, and N122.

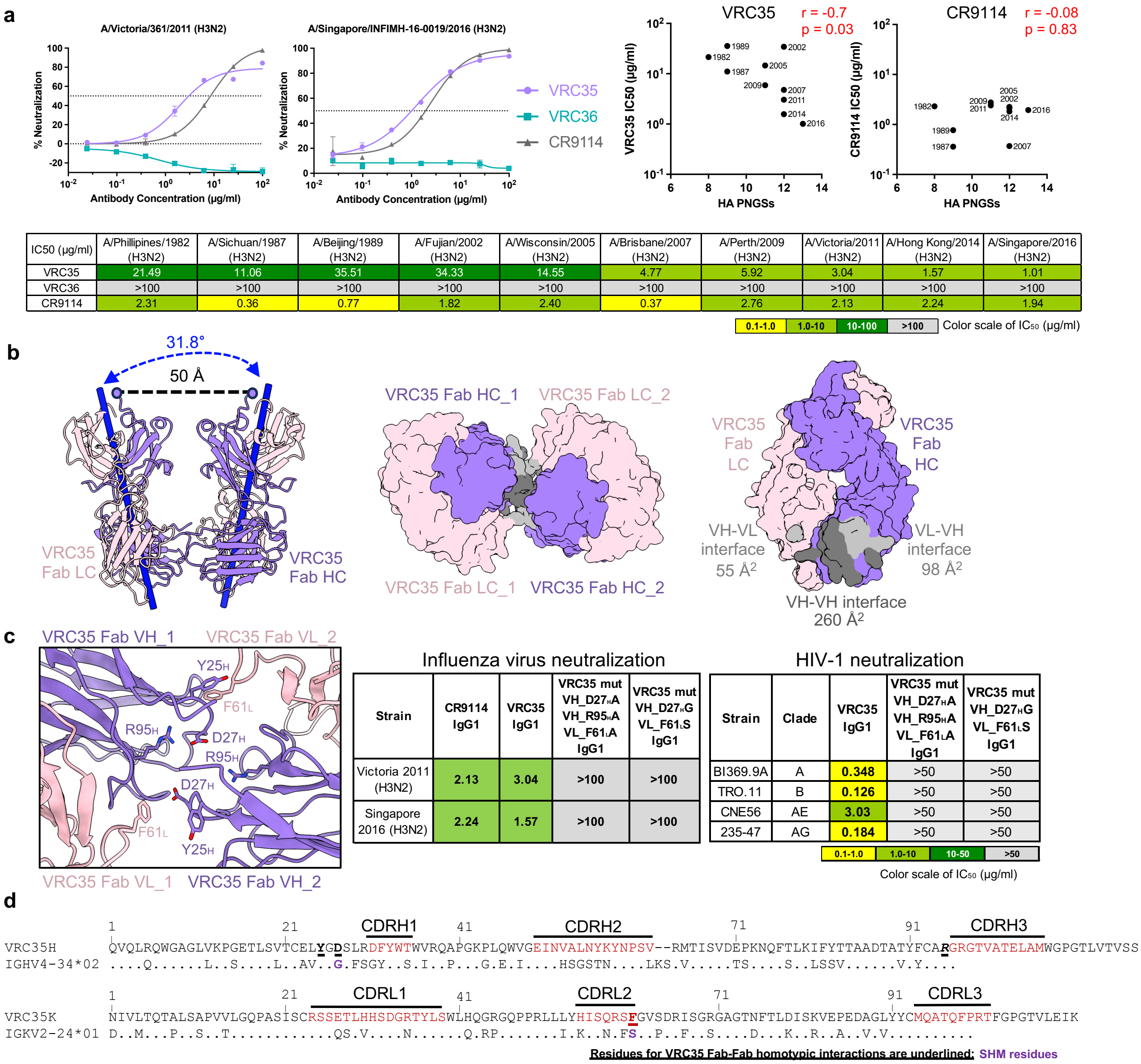

IC50 (µg/ml)	A/Phillipines/1982 (H3N2)	A/Sichuan/1987 (H3N2)	A/Beijing/1989 (H3N2)	A/Fujian/2002 (H3N2)	A/Wisconsin/2005 (H3N2)	A/Brisbane/2007 (H3N2)	A/Perth/2009 (H3N2)	A/Victoria/2011 (H3N2)	A/Hong Kong/2014 (H3N2)	A/Singapore/2016 (H3N2)
VRC35	21.49	11.06	35.51	34.33	14.55	4.77	5.92	3.04	1.57	1.01
VRC36	>100	>100	>100	>100	>100	>100	>100	>100	>100	>100
CR9114	2.31	0.36	0.77	1.82	2.40	0.37	2.76	2.13	2.24	1.94

0.1-1.0 | 1.0-10 | 10-100 | >100 Color scale of IC50 (µg/ml)

Influenza virus neutralization

Strain	CR9114 IgG1	VRC35 IgG1	VRC35 mut VH_D27HA VH_R95HA VL_F61LA IgG1	VRC35 mut VH_D27HG VL_F61LS IgG1
Victoria 2011 (H3N2)	2.13	3.04	>100	>100
Singapore 2016 (H3N2)	2.24	1.57	>100	>100

HIV-1 neutralization

Strain	Clade	VRC35 IgG1	VRC35 mut VH_D27HA VH_R95HA VL_F61LA IgG1	VRC35 mut VH_D27HG VL_F61LS IgG1
BI369.9A	A	0.348	>50	>50
TRO.11	B	0.126	>50	>50
CNE56	AE	3.03	>50	>50
235-47	AG	0.184	>50	>50

0.1-1.0 | 1.0-10 | 10-50 | >50 Color scale of IC50 (µg/ml)

Fig. 2 VRC35 neutralized highly glycosylated viruses through homotypic assembly. a VRC35 neutralizes multiple influenza H3 strains in reporter virus assay. Values were shown as IC_{50} (µg/ml). Spearman's rank correlation between the number of predicted *N*-linked glycosylation sites (PNGSs) on HA and neutralization IC_{50} values revealed a significant correlation for VRC35 but not for CR9114. **b** Dimeric VRC35 Fabs were oriented at a 31.8° rotation and separated by ~50 Å apart between CH1 termini. VH-VH and VH-VL homotypic interfaces were shown in dark and light gray, respectively, with buried surface areas (Å^2) indicated. **c** Key residues mediating VRC35 Fab–Fab homotypic interactions. Structural model of the VRC35 homotypic interface (left). Neutralization analyses of alanine substitutions (D27A, R95A, and F61A) and germline reversion mutations (D27G and F61S) demonstrated marked reductions in neutralization against representative influenza and HIV-1 strains. Values are IC_{50} (µg/ml). **d** Sequence alignment of mature and germline VRC35 heavy and light chains. Complementarity-determining regions (CDRs) are highlighted in red. Residues involved in homotypic interactions are underlined, and somatic hypermutation (SHM) residues contributing to these interactions are shown in purple.

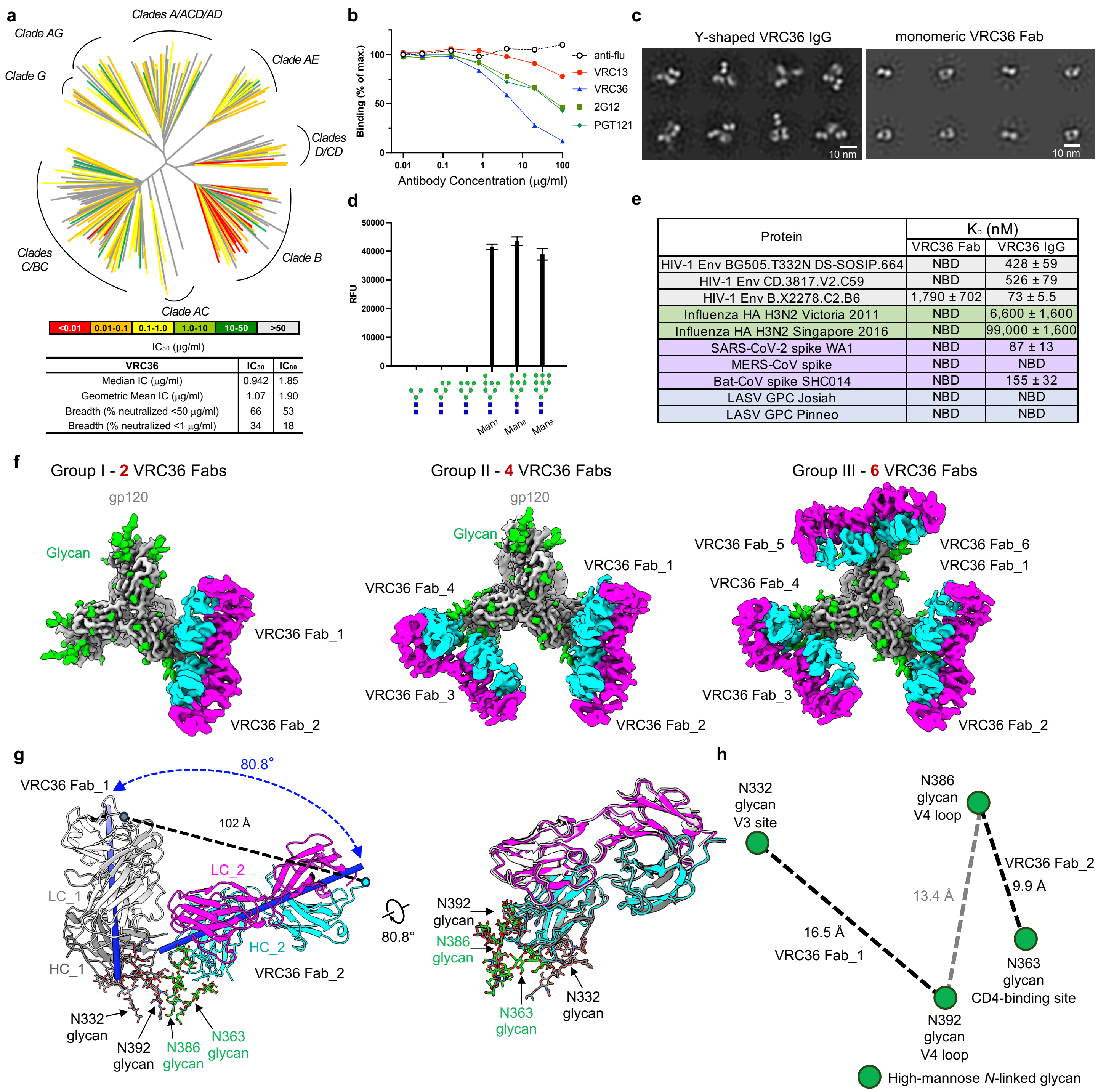

VRC36	IC_{50}	IC_{80}
Median IC (μg/ml)	0.942	1.85
Geometric Mean IC (μg/ml)	1.07	1.90
Breadth (% neutralized <50 μg/ml)	66	53
Breadth (% neutralized <1 μg/ml)	34	18

Protein	K_D (nM)	
	VRC36 Fab	VRC36 IgG
HIV-1 Env BG505.T332N DS-SOSIP.664	NBD	428 ± 59
HIV-1 Env CD.3817.V2.C59	NBD	526 ± 79
HIV-1 Env B.X2278.C2.B6	1,790 ± 702	73 ± 5.5
Influenza HA H3N2 Victoria 2011	NBD	6,600 ± 1,600
Influenza HA H3N2 Singapore 2016	NBD	99,000 ± 1,600
SARS-CoV-2 spike WA1	NBD	87 ± 13
MERS-CoV spike	NBD	NBD
Bat-CoV spike SHC014	NBD	155 ± 32
LASV GPC Josiah	NBD	NBD
LASV GPC Pinneo	NBD	NBD

Fig. 3 VRC36 formed inter-IgG homotypic assemblies on HIV-1 Env. a Neutralization dendrogram of the 208-strain virus panel colored by VRC36 IC50 titers. **b** VRC36 binding competition assay. Lines indicate binding of labeled VRC36 to TRO.11-Env trimer-expressing 293T cells in the presence of the indicated serially diluted unlabeled competitor antibodies as measured by flow cytometry. **c** Negative staining-EM 2D class averages showed canonical Y-shaped VRC36 IgG and monomeric Fab. **d** Binding of VRC36 IgG to Man_7, Man_8 and Man_9 glycans on a high-density microarray. Error bars indicated standard deviations. **e** SPR analysis of VRC36 Fab and IgG binding to HIV-1 Env, influenza HA, coronavirus spikes, and LASV GPC. **f** Cryo-EM analysis revealed dimerized VRC36 Fabs bound to BG505 DS-SOSIP.664 Env. Fabs bound to Env were classified into three groups with 2, 4, or 6 Fabs per Env trimer. The VRC36 Fab heavy chain (HC) and light chain (LC) were colored cyan and magenta, respectively. HIV-1 Env gp120 and gp41 were shown in light grey and dark grey. Glycans were shown in green. **g** The dimeric VRC36 Fabs were oriented at an 80.8° rotation and separated by ~102 Å between CH1 termini. Each Fab interacted with two glycans. After rotation, paired VRC36 Fabs and their interacting glycans aligned with each other. **h** Glycan matrix of N332, N392, N386, and N363. The Cα-Cα distance between N332 and N392 was 16.5 Å, supporting VRC36 Fab_1 binding. The Cα-Cα distance between N386 and N363 was 9.9 Å, supporting VRC36 Fab_2 binding. The observed 13.4 Å distance between merged glycans N386 and N392 corresponded to the homotypic interactions formed between two separate IgGs.

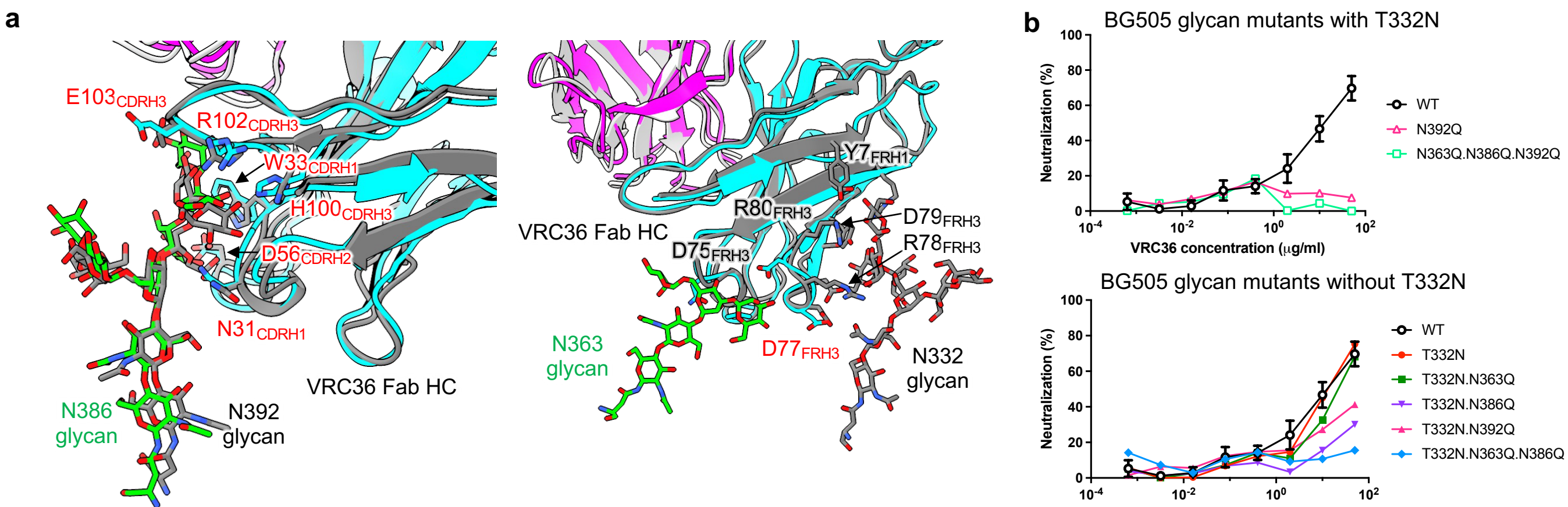

c

Strain	Clade	WT IC50 (μg/ml)	Fold Change N31A	W33A	S53A	D56A	H100A	5 mut
BG505.W6M.C2	A	9.45	>5.3	>5.3	>5.3	>5.3	>5.3	>5.3
BG505.W6M.C2.T332N	A	11.9	>4.2	>4.2	>4.2	>4.2	>4.2	>4.2
JRCSF.JB	B	0.565	>88	>88	>88	2.8	>88	>88
TRO.11	B	0.016	15	18	12	5.5	19	>3125
JRFL.JB	B	0.094	28.6	25	9.6	2.5	14	>532

WT IC50 (μg/ml)

0.01-0.1	0.1-1.0	1.0-10	10-50	>50

Mutant fold change (Mut/WT)

potency reduced		unchanged
>100	3-100	0.33-3

d

N-linked glycan	N332	N363	N386	N392
Percentage of glycan conservation in 208 strains (%)	69.2%	12.5%	88.0%	81.7%
Percentage of glycan conservation in 138 neutralized strains (%)	74.6%	13.8%	94.9%	91.3%
Buried surface area (Å^2)	458	270	490	490

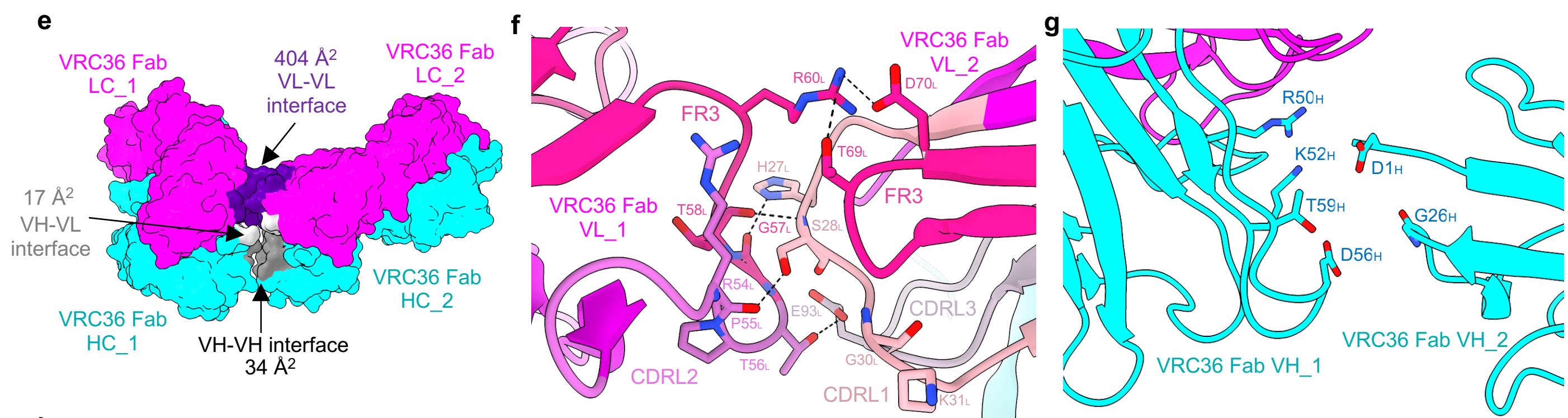

h

```
           1                   21   CDRH1         41                      CDRH2                 71                  91       CDRH3    111
VRC36H     DVKLVAYGGAVVRPGGLVRISCQASGFNFVNAWMSWVRQAPGRGPEWVGRIKSRADGWTVDYNSRVKGRFTISRDDDRDRMYLHTTNLKPEDTGIYYCVHLREKGDDWSSGSQVIVS
IGHV3-15*02 E.Q..ES...L.K...SL.L..A....T.S............K.L........KT..G.T..AAP..........SKNTL..QMNS..T...AV...

           1                   21   CDRL1         41        CDRL2           71                  CDRL3   101
VRC36K     EIVLTQSPATVCLSAGETVRLTCTASHSVGKSLSWLQQRPGQAPRPLISEVSARPTGTPRRFSGSGSETDFTLTIDSLEAEDSAMYYCQQRGEWPFTFGQGTKVEIN
IGKV3-11*01 ..........LS..P..RAT.S.R..Q..SSY.A.Y..K......L..YDA.N.A..I.A.......G.......S...P..F.V......SN..
```

Residues involved in Fab-Fab homotypic interactions are underlined; **VL-SHM residues;** Recurrent and *non-recurrent* residues for VH-glycan interaction

i

Strain	Clade	WT IC50 (μg/ml)	Fold change - mutations disrupted homotypic interactions: S28R	S28W	G30R	G30W	S28R G30R	S28R G30W	S28W G30R	S28W G30W
KER2018.11	A	0.422	>118	46	19	1.9	>118	>118	>118	>118
BI369.9A	A	0.059	39	12	9.6	2.7	57	99	39	20
BG505.W6M.C2	A	14.1	>3.5	>3.5	>3.5	0.8	>3.5	>3.5	>3.5	>3.5
BG505.W6M.C2.T332N	A	12.2	>4.1	>4.1	>4.1	1.4	>4.1	>4.1	>4.1	>4.1
JRCSF.JB	B	0.848	>59	>59	8.8	2.4	>59	>59	>59	>59
TRO.11	B	0.018	21	10	2.1	1.6	32	53	42	28
TRJO.58	B	0.03	65	24	6.0	2.1	110	268	79	41
WITO.33	B	0.195	>256	76	11	3.6	>256	>256	>256	>256
CNE58	C	0.083	21	12	4.2	2.0	20	31	18	9.1
ZM106.9	C	0.088	38	21	5.2	2.2	102	198	134	50
M02138	AE	0.084	58	30	3.2	1.8	112	381	99	39
CNE56	AE	0.251	55	21	16	4.0	128	184	114	56
Q168.a2	AD	12.4	>4.0	>4.0	>4.0	1.5	>4.0	>4.0	>4.0	>4.0
235-47	AG	0.167	28	7.5	5.0	2.0	25	45	17	12

Strain	Fold change - mutations reverted to germline amino acids: H27Q	G30S	K31S	H27Q G30S K31S	P55A	T58I	R60A	P55A T58I R60A	6 mut
KER2018.11	37	2.1	0.9	13	3.2	2.7	6.5	>118	>118
BI369.9A	13	1.8	0.7	8.6	3.2	1.7	7.5	47	67
BG505.W6M.C2	>3.5	0.9	0.5	>3.5	1.5	0.7	2.2	>3.5	>3.5
BG505.W6M.C2.T332N	>4.1	1.9	0.7	>4.1	2.2	1.3	>4.1	>4.1	>4.1
JRCSF.JB	9.7	1.2	0.6	5.0	1.5	0.9	2.6	>59	>59
TRO.11	2.7	1.6	1.2	2.5	0.9	0.9	1.2	5.7	69
TRJO.58	4.1	1.4	0.8	3.1	1.6	1.5	2.7	41	129
WITO.33	18	2.6	1.7	14	2.3	2.7	6.4	159	>256
CNE58	3.9	1.3	1.0	3.8	1.3	0.8	1.7	15	41
ZM106.9	8.6	1.9	1.1	5.8	1.8	1.4	2.3	16	230
M02138	6.9	2.9	1.7	7.0	1.4	1.4	2.2	19	208
CNE56	12	2.3	0.7	10	2.1	1.8	4.3	50	178
Q168.a2	3.3	2.3	0.7	>4.0	1.3	1.1	3.2	>4.0	>4.0
235-47	4.9	2.3	1.0	4.3	1.3	1.3	2.0	15	43

WT IC50 (μg/ml)

0.01-0.1
0.1-1.0
1.0-10
10-50
>50

Fold change (Mut/WT)

>100	potency reduced
3-100	potency reduced
0.33-3	unchanged

Fig. 4 Inter-IgG homotypic assembly underlay VRC36 neutralization of HIV-1. a Superimposition of N386 and N392. Two VRC36 Fabs used the same residues from CDRH1, CDRH2 and CDRH3 to interact with glycans N386 and N392. Glycans N363 and N332 were engaged by non-repeated residues from FR1 and FR3 of the heavy chain. **b** Neutralization assessments of BG505 glycan mutants with and without T332N. **c** Neutralization assessments of VRC36 IgG mutants. **d** Conservation analysis of N332, N363, N386, and N392 glycans across 208 HIV-1 strains and 138 neutralized strains, with corresponding buried surface areas. **e** Surface representation of dimeric VRC36 Fabs. VH-VH, VH-VL and VL-VL homotypic interfaces were shown in dark grey, light grey, and purple, respectively. Buried surface area (Å^2) were indicated. Hydrogen bonds and salt bridges at the VL-VL interface were shown as dotted lines. **f** Interactions at the VRC36 VL-VL interface. Hydrogen bonds and salt bridges were shown as dotted lines. **g** Interactions at the VRC36 VH-VH interface. Amino acid proximity indicated buried surface shown in **e.** Hydrogen bonds and salt bridges were not observed. **h** Sequence alignment of mature and germline VRC36 heavy and light chains. Residues involved in Fab-Fab homotypic interactions were underlined; Residues involved in VH-glycan interactions were colored red, and non-recurrent residues were italicized. SHM residues involved in homotypic interactions were shown in bold purple. **i** Neutralization characteristics of VRC36 light chain mutants. Values are shown as fold change (Mut/WT). Left, mutations to disrupt or enhance VL-VL homotypic interactions. Right, mutations to revert to germline amino acids. 6 mut indicates the mutations including H27Q, G30S, K31S, P55A, T58I, and R60A.

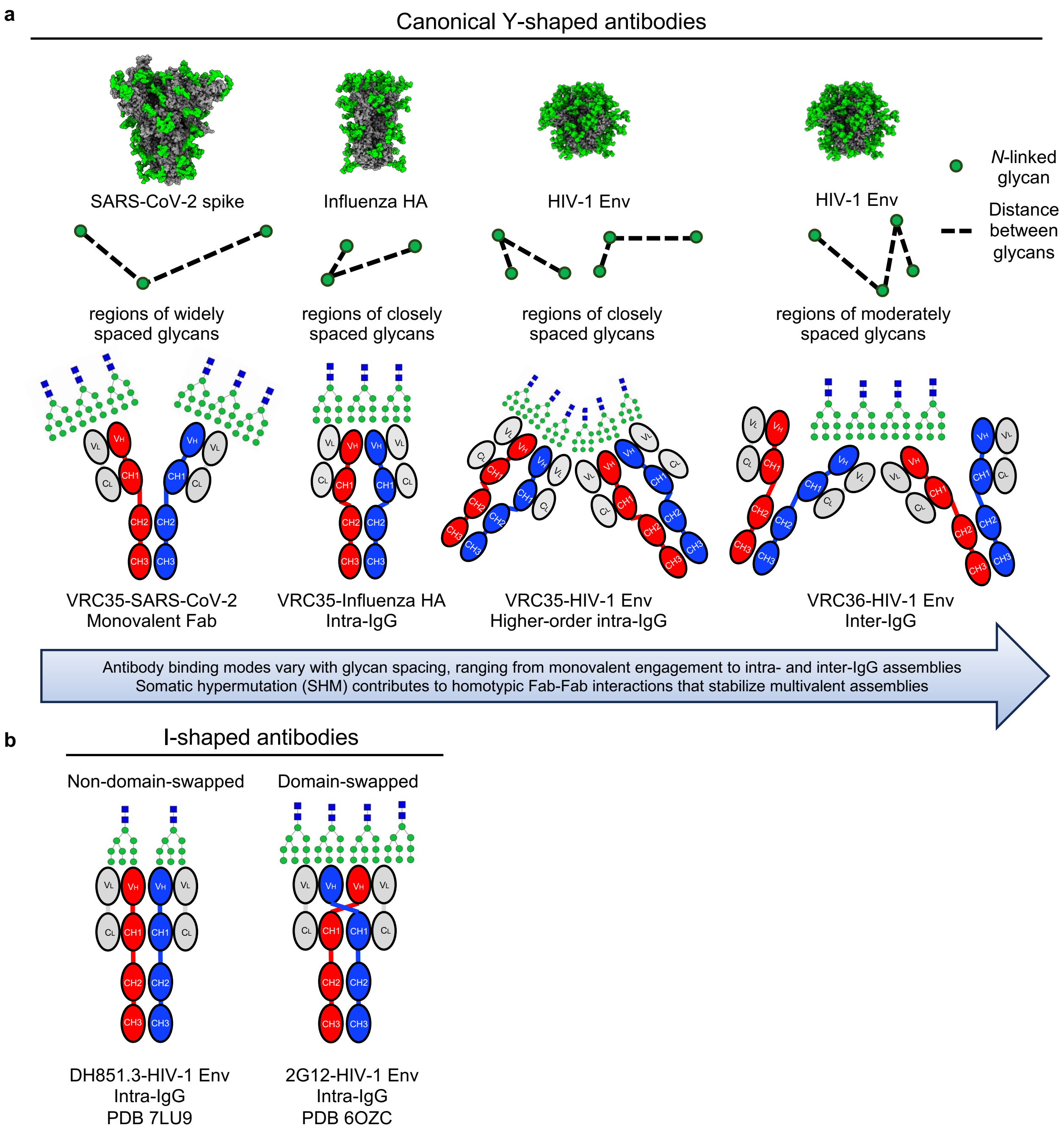

Fig. 5 Structural models of glycan spacing-dependent antibody binding modes. a Canonical Y-shaped antibodies exhibit multiple binding configurations that vary with glycan organization on viral surfaces, reflecting the geometric constraints imposed by glycan spacing. Fully glycosylated models of SARS-CoV-2 spike (LP.8.1.9), influenza hemagglutinin (H3N2 Victoria 2011), and HIV-1 Env (BG505 DS-SOSIP.664) illustrate differences in glycan density and spatial distribution. Notably, heterogeneous glycan organization can occur across distinct regions of the same viral surface, as exemplified by HIV-1 Env, which contains both closely spaced and moderately spaced glycan regions. Across viral glycoproteins exhibiting different glycan spacing regimes, antibody binding modes range from monovalent binding (widely spaced glycans), to intra-IgG Fab-Fab assemblies (closely spaced glycans), and to inter-IgG assemblies (moderately spaced glycans). Representative models are shown for VRC35 bound to SARS-CoV-2 spike (monovalent Fab), VRC35 bound to influenza hemagglutinin (intra-IgG assembly), VRC35 bound to HIV-1 Env (intra-IgG with higher-order assembly), and VRC36 bound to HIV-1 Env (inter-IgG assembly). Antibody heavy chains are shown in red and blue to distinguish individual Fab molecules, whereas light chains are shown in gray. Green circles represent *N*-linked glycans, and dashed lines indicate distances measured between neighboring glycans. These configurations illustrate that glycan organization constrains the range of accessible antibody binding modes. Fully glycosylated models were generated using GLYCAM-Web prediction tools. **b** In contrast, I-shaped glycan-reactive antibodies adopt preconfigured Fab geometries that enable multivalent glycan engagement independent of glycan spacing. Examples include the non-domain-swapped antibody DH851.3 and the domain-swapped antibody 2G12, both bound to HIV-1 Env. Together, these models highlight distinct structural strategies for glycan recognition, in which canonical antibodies exhibit geometry-dependent flexibility, whereas I-shaped antibodies rely on preconfigured architectures. While glycan spacing provides a useful structural framework for interpreting these binding modes, additional factors such as glycan composition and epitope accessibility may also contribute.

a Characterization of N26 Serum

Clade	Virus ID	N26 Serum (ID50)
A	KER2018.11	449
	Q23.17	402
	Q769.h5	359
	RW020.2	173
	BG505.W6M.C2	88
	0439.v5.c1	142
	0260.v5.c36	71
	BB539.2B13	125
	Q842.d12	555
AD	Q168.a2	667
B	6101.1	298
	Bal.01	1532
	BG1168.01	119
	CAAN.A2	631
	JRCSF.JB	717
	JRFL.JB	3666
	PVO.04	1875
	THRO.18	166
	TRJO.58	150
	TRO.11	1701
	YU2.DG	309
C	DU156.12	139
	DU422.01	418
	ZA012.29	100
	ZM106.9	431
	ZM55.28a	100
	CAP244.D3	187
	CAP256.206.c9	37
	96ZM651.02	386
	286.36	760
	ZM233.6	<20
	breadth	97%
	geometric mean ID50	309

Epitope fingerprint

CD4 binding site	VRC01-like	
	b12-like	
	CD4-like	
	HJ16-like	0.17
gp120:gp41 interface	8ANC195-like	
V2 apex	PG9-like	0.03
Glycan-V3	PGT128-like	
	2G12-like	0.73
MPER	2F5-like	0.08
	10E8-like	

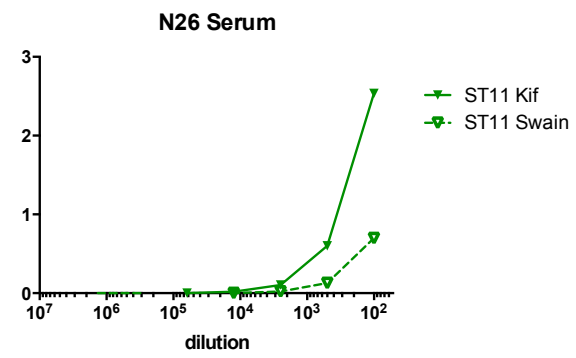

ID50
>1000
500-1000
100-500
20-100
<20

b Isolation of VRC35 and VRC36 families from donor N26

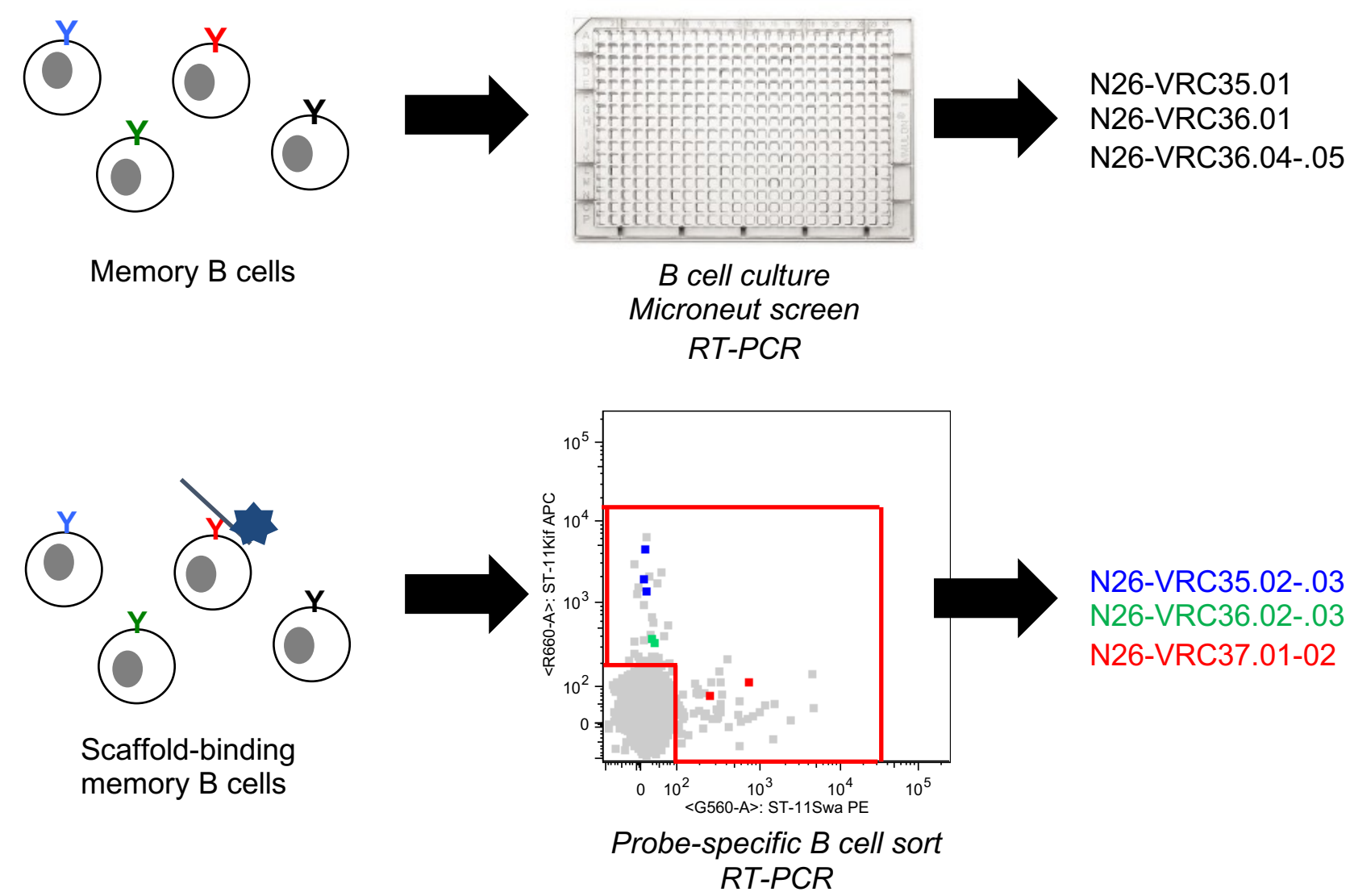

c

Antibody	VH Gene	Vκ Gene	Somatic Mutations (nt) VH Gene	Somatic Mutations (nt) Vκ Gene	CDRH3 Sequence	CDRH3 Length (aa)	CDRL3 Sequence	CDRL3 Length (aa)
N26-VRC35.01	4-34	2-24	28%	20%	ARGRGTVATELAM	13	MQATQFPRT	9
N26-VRC35.02			32%	19%	ARGRGTVASEIGL		MQGTQFPRT	
N26-VRC35.03			34%	20%	ARGRGTVASEIGL		MQGTQFPRT	
N26-VRC35.04			22%	23%	ARGRGVVPTEIAV		MQATEFPRT	
N26-VRC36.01	3-15	3-11/NL5	26%	20%	VHLREKGDD	9	QQRGEWPYT	9
N26-VRC36.02			25%	22%	VHLRERGDD		QQRGEWPFT	
N26-VRC36.03			25%	20%	VHLRERGDD		QQRGEWPFT	
N26-VRC36.04			25%	23%	VHLREKGDD		QQRGEWPYT	
N26-VRC36.05			25%	22%	VHLREKGDD		QQRGEWPYT	
N26-VRC37.01	1-69	3-20	29%	15%	ARMSGSYESGHYAGMDV	17	QQYGLSPYT	9
N26-VRC37.02			26%	16%	ARMSGSYGSGHYSGMDV		QQYALSPFT	

d

Clade	Virus	N26-VRC35.01	N26-VRC35.02	N26-VRC35.03	N26-VRC36.01	N26-VRC36.02	N26-VRC36.03	N26-VRC36.04	N26-VRC36.05	N26-VRC37.01	N26-VRC37.02
A	KER2018.11	>50	>50	>50	0.124	0.106	0.272	0.220	0.967	>50	>50
	Q23.17	0.859	28.6	>50	1.18	1.84	5.21	1.14	3.13	>50	24.2
	Q769.h5	>50	>50	>50	6.95	1.36	0.758	1.71	>50	>50	>50
	RW020.2	0.546	1.43	18.7	>50	>50	>50	>50	>50	>50	>50
	BG505.W6M.C2	0.986	19.3	>50	1.70	0.663	1.40	1.60	1.64	>50	>50
	0439.v5.c1	2.53	8.06	>50	0.202	0.475	0.931	0.200	0.394	>50	>50
	0260.v5.c36	1.08	2.85	10.4	0.802	0.813	1.58	0.631	1.28	>50	>50
	BB539.2B13	>50	>50	>50	>50	>50	>50	>50	>50	>50	>50
	Q842.d12	0.529	34.9	>50	1.93	1.07	3.04	2.63	5.48	>50	>50
AD	Q168.a2	>50	>50	>50	4.60	3.19	2.53	2.81	9.32	>50	>50
B	6101.1	0.392	2.40	6.41	1.53	1.76	6.43	2.47	5.22	3.21	6.73
	Bal.01	2.10	19.7	>50	0.611	0.502	1.90	0.755	3.19	10.3	22.8
	BG1168.01	>50	>50	>50	2.29	2.96	10.9	4.67	7.40	>50	>50
	CAAN.A2	3.68	38.8	>50	0.105	0.200	0.426	0.174	1.14	>50	>50
	JRCSF.JB	1.25	30.9	>50	0.613	0.419	0.897	0.269	3.19	>50	>50
	JRFL.JB	5.09	>50	>50	0.128	0.451	1.24	0.370	0.465	35.0	18.7
	PVO.04	0.568	2.76	41.9	1.01	1.32	2.10	0.833	3.57	>50	>50
	THRO.18	3.56	>50	>50	13.4	6.87	>50	>50	>50	>50	>50
	TRJO.58	2.33	>50	>50	0.138	0.511	0.834	0.223	0.297	>50	>50
	TRO.11	0.212	0.905	0.600	0.020	0.047	0.087	0.041	0.092	8.93	10.4
	YU2.DG	0.400	0.601	1.63	7.58	>50	>50	9.24	15.8	>50	>50
C	DU156.12	1.57	>50	>50	>50	>50	>50	>50	>50	>50	>50
	DU422.01	>50	>50	>50	>50	>50	>50	>50	>50	>50	>50
	ZA012.29	>50	>50	>50	>50	>50	>50	>50	>50	>50	>50
	ZM106.9	9.63	>50	>50	0.165	0.186	0.231	0.153	0.335	>50	>50
	ZM55.28a	>50	>50	>50	>50	>50	>50	>50	>50	>50	>50
	CAP244.D3	2.07	>50	>50	0.747	0.690	1.12	0.709	1.34	>50	>50
	CAP256.206.c9	2.22	3.22	>50	>50	18.5	>50	17.0	>50	>50	>50
	96ZM651.02	14.6	>50	>50	>50	>50	>50	>50	>50	>50	>50
	286.36	0.185	0.258	0.279	>50	>50	>50	>50	>50	>50	>50
	ZM233.6	1.13	9.77	>50	>50	>50	>50	>50	>50	>50	>50
	% Neutralized at IC50<50	74%	52%	23%	68%	68%	61%	64.5%	61%	13%	16%
	Geomean IC50 of neutralized viruses	1.36	5.34	3.92	0.73	0.81	1.22	0.81	1.68	10.08	14.85

IC50 μg/ml
<0.001
0.001-0.01
0.01-0.1
0.1-1
1-10
10-50
>50

e

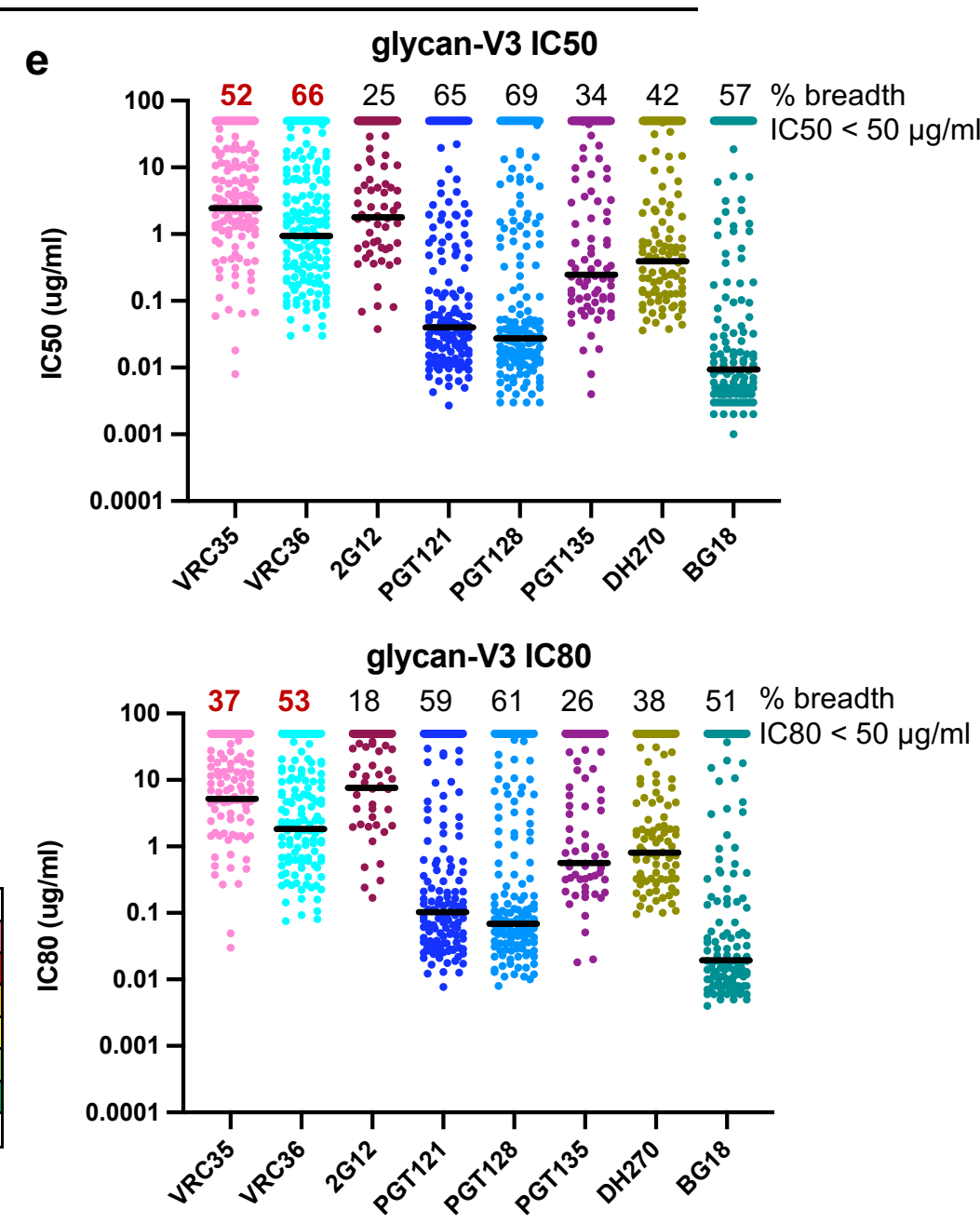

Supplementary Figure 1. Isolation of VRC35, VRC36, and VRC37 families from donor N26. a Characterization of N26 serum. Serum was tested for neutralization of 31 Env-pseudoviruses. Data for 20 viruses was used to predict epitopes by a fingerprinting algorithm, which compares neutralization patterns of a serum of interest with those of HIV-1 bNAbs targeting known epitopes. Darker fields/higher numbers indicate greater similarity to a given bNAb's virus neutralization. Serum was tested by ELISA for binding to scaffolded mini-V3 protein ST11 produced in cells treated with swainsonine (ST11-swain) or kifunensine (ST11-kif). **b** VRC35, VRC36, VRC37 families were isolated from donor N26 memory B cells by high-throughput B cell culture and microneutralization, or by staining with ST11-kif-APC and ST11-swa-PE probes and single-cell sort, followed by multiplex RT-PCR. **c** Immunogenetics of VRC35, VRC36, and VRC37 lineages. **d** Neutralization activity of VRC35, VRC36, and VRC37 antibodies. VRC35.01 and VRC36.01 are referred to as VRC35 and VRC36, respectively, in other figures. **e** Neutralization IC50 (μg/ml) and IC80 (μg/ml) of VRC35, VRC36, and other glycan-V3 bNAbs against 208 Env-pseudoviruses.

a Neutralization pattern correlations and antibody clustering

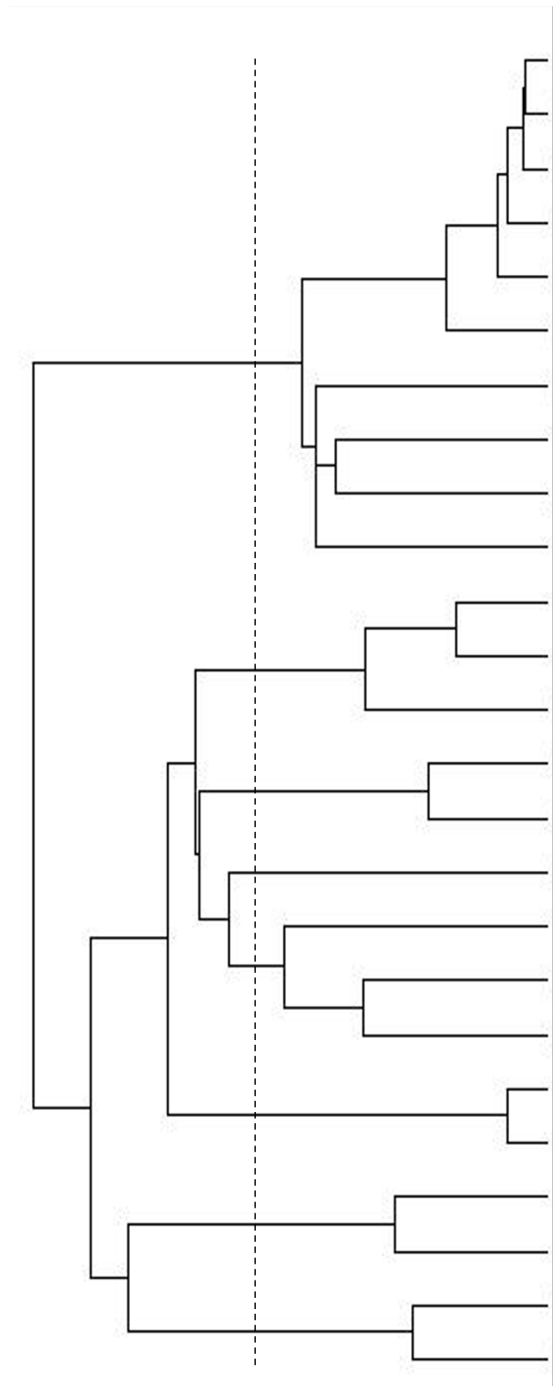

Antibody	10-1074	PGT121	VRC41.01	DH270	BG18	PGT128	PGT135	VRC36	2G12	VRC35	3BNC117	VRC01	VRC13	VRC44.01	35O22	8ANC195	VRC-PG05	SF12	2F5	10E8	4E10	PG9	CAP256-VRC26.25	VRC34.01	PGT151	Epitope
10-1074	1.00	0.86	0.84	0.77	0.81	0.63	0.43	0.22	0.32	0.34	0.09	0.05	-0.02	0.05	0.06	-0.03	-0.16	-0.15	-0.25	0.12	0.05	-0.13	0.00	0.16	0.18	Glycan-V3
PGT121	0.86	1.00	0.79	0.74	0.71	0.62	0.40	0.19	0.25	0.27	0.09	0.08	-0.02	0.02	0.05	-0.01	-0.12	-0.14	-0.29	0.08	-0.01	0.01	0.08	0.21	0.19	
VRC41.01	0.84	0.79	1.00	0.75	0.76	0.67	0.43	0.21	0.22	0.33	0.09	0.06	-0.06	-0.04	-0.03	-0.02	-0.14	-0.21	-0.29	0.06	-0.03	-0.03	0.04	0.15	0.22	
DH270	0.77	0.74	0.75	1.00	0.72	0.61	0.36	0.21	0.26	0.35	0.17	0.10	-0.01	0.02	0.07	0.06	-0.08	-0.06	-0.23	0.07	-0.03	-0.13	-0.07	0.13	0.15	
BG18	0.81	0.71	0.76	0.72	1.00	0.60	0.53	0.43	0.47	0.43	0.06	0.04	-0.09	-0.01	-0.02	0.04	-0.12	-0.09	-0.18	0.07	-0.01	-0.07	-0.09	0.12	0.17	
PGT128	0.63	0.62	0.67	0.61	0.60	1.00	0.20	0.21	0.30	0.27	0.13	0.03	-0.03	0.02	0.09	0.17	-0.10	0.08	-0.02	0.07	0.05	0.00	-0.01	0.11	0.13	
PGT135	0.43	0.40	0.43	0.36	0.53	0.20	1.00	0.39	0.27	0.29	0.14	0.13	0.03	-0.03	0.01	-0.06	-0.05	-0.19	-0.09	0.01	-0.05	-0.07	-0.07	0.02	0.04	
VRC36	0.22	0.19	0.21	0.21	0.43	0.21	0.39	1.00	0.39	0.14	0.02	-0.03	-0.08	-0.11	0.00	0.05	0.12	0.08	0.11	0.07	-0.03	-0.06	-0.16	0.01	0.06	
2G12	0.32	0.25	0.22	0.26	0.47	0.30	0.27	0.39	1.00	0.35	0.15	0.04	0.10	0.15	0.05	0.08	-0.17	0.11	0.17	0.18	0.07	-0.11	-0.24	0.05	0.07	
VRC35	0.34	0.27	0.33	0.35	0.43	0.27	0.29	0.14	0.35	1.00	0.18	0.06	-0.04	0.04	0.09	0.10	-0.26	-0.13	-0.11	0.07	-0.07	-0.14	-0.13	0.00	0.05	
3BNC117	0.09	0.09	0.09	0.17	0.06	0.13	0.14	0.02	0.15	0.18	1.00	0.66	0.48	0.13	0.25	0.13	0.03	0.13	0.21	0.09	0.00	0.05	-0.18	-0.01	0.01	CD4bs
VRC01	0.05	0.08	0.06	0.10	0.04	0.03	0.13	-0.03	0.04	0.06	0.66	1.00	0.37	0.05	0.15	0.00	-0.01	0.11	0.04	-0.02	-0.11	0.17	0.03	0.11	0.11	
VRC13	-0.02	-0.02	-0.06	-0.01	-0.09	-0.03	0.03	-0.08	0.10	-0.04	0.48	0.37	1.00	0.14	0.12	0.12	0.08	0.11	0.25	0.14	0.10	0.05	-0.02	-0.18	0.02	
VRC44.01	0.05	0.02	-0.04	0.02	-0.01	0.02	-0.03	-0.11	0.15	0.04	0.13	0.05	0.14	1.00	0.51	0.16	0.03	0.16	0.13	0.10	0.08	-0.01	-0.06	0.03	-0.13	gp120-gp41 Interface
35O22	0.06	0.05	-0.03	0.07	-0.02	0.09	0.01	0.00	0.05	0.09	0.25	0.15	0.12	0.51	1.00	0.06	0.04	0.20	0.09	0.05	0.02	0.07	-0.01	0.15	0.00	
8ANC195	-0.03	-0.01	-0.02	0.06	0.04	0.17	-0.06	0.05	0.08	0.10	0.13	0.00	0.12	0.16	0.06	1.00	0.02	0.22	0.18	0.19	0.11	-0.09	-0.08	-0.21	-0.08	
VRC-PG05	-0.16	-0.12	-0.14	-0.08	-0.12	-0.10	-0.05	0.12	-0.17	-0.26	0.03	-0.01	0.08	0.03	0.04	0.02	1.00	0.30	0.17	0.11	0.11	0.06	0.01	-0.15	-0.12	Silent Face
SF12	-0.15	-0.14	-0.21	-0.06	-0.09	0.08	-0.19	0.08	0.11	-0.13	0.13	0.11	0.11	0.16	0.20	0.22	0.30	1.00	0.41	0.14	0.07	-0.03	-0.08	-0.07	-0.05	
2F5	-0.25	-0.29	-0.29	-0.23	-0.18	-0.02	-0.09	0.11	0.17	-0.11	0.21	0.04	0.25	0.13	0.09	0.18	0.17	0.41	1.00	0.25	0.25	-0.04	-0.20	-0.16	-0.05	MPER N-term
10E8	0.12	0.08	0.06	0.07	0.07	0.07	0.01	0.07	0.18	0.07	0.09	-0.02	0.14	0.10	0.05	0.19	0.11	0.14	0.25	1.00	0.81	-0.08	-0.14	-0.16	-0.22	MPER C-term
4E10	0.05	-0.01	-0.03	-0.03	-0.01	0.05	-0.05	-0.03	0.07	-0.07	0.00	-0.11	0.10	0.08	0.02	0.11	0.11	0.07	0.25	0.81	1.00	-0.01	-0.04	-0.15	-0.22	
PG9	-0.13	0.01	-0.03	-0.13	-0.07	0.00	-0.07	-0.06	-0.11	-0.14	0.05	0.17	0.05	-0.01	0.07	-0.09	0.06	-0.03	-0.04	-0.08	-0.01	1.00	0.46	0.16	0.07	V2 Apex
CAP256-VRC26.25	0.00	0.08	0.04	-0.07	-0.09	-0.01	-0.07	-0.16	-0.24	-0.13	-0.18	0.03	-0.02	-0.06	-0.01	-0.08	0.01	-0.08	-0.20	-0.14	-0.04	0.46	1.00	0.15	0.12	
VRC34.01	0.16	0.21	0.15	0.13	0.12	0.11	0.02	0.01	0.05	0.00	-0.01	0.11	-0.18	0.03	0.15	-0.21	-0.15	-0.07	-0.16	-0.16	-0.15	0.16	0.15	1.00	0.49	Fusion Peptide
PGT151	0.18	0.19	0.22	0.15	0.17	0.13	0.04	0.06	0.07	0.05	0.01	0.11	0.02	-0.13	0.00	-0.08	-0.12	-0.05	-0.05	-0.22	-0.22	0.07	0.12	0.49	1.00	

b Fine epitope mapping: neutralization of Env mutants

	JRCSF.DB mutant							
**Antibody**	**N262A**	**N295**	**N301A**	**N332A**	**N339A**	**N386A**	**N392A**	**T450A**
VRC35	0.6	>45	9.7	>45	2.1	1.6	2.7	>30
VRC36	0.5	2.2	1.0	3.1	1.7	>300	>300	1.3
PGT121	0.4	1.1	1.0	>1900	1.0	1.2	1.5	1.0
PGT128	ND	1.3	5.8	8.2	2.0	1.3	2.3	1.3
2G12	ND	>80	1.2	>80	18	13	>80	2.6
PGT135	ND	17	>600	>130	1.5	2.6	>130	2.1

Mutant fold change (IC50 Mut/WT)		
potency knocked down		Unchanged
>100	3-100	0.33-3

c Correlations between the HIV-1 Env PNGSs and neutralization IC50

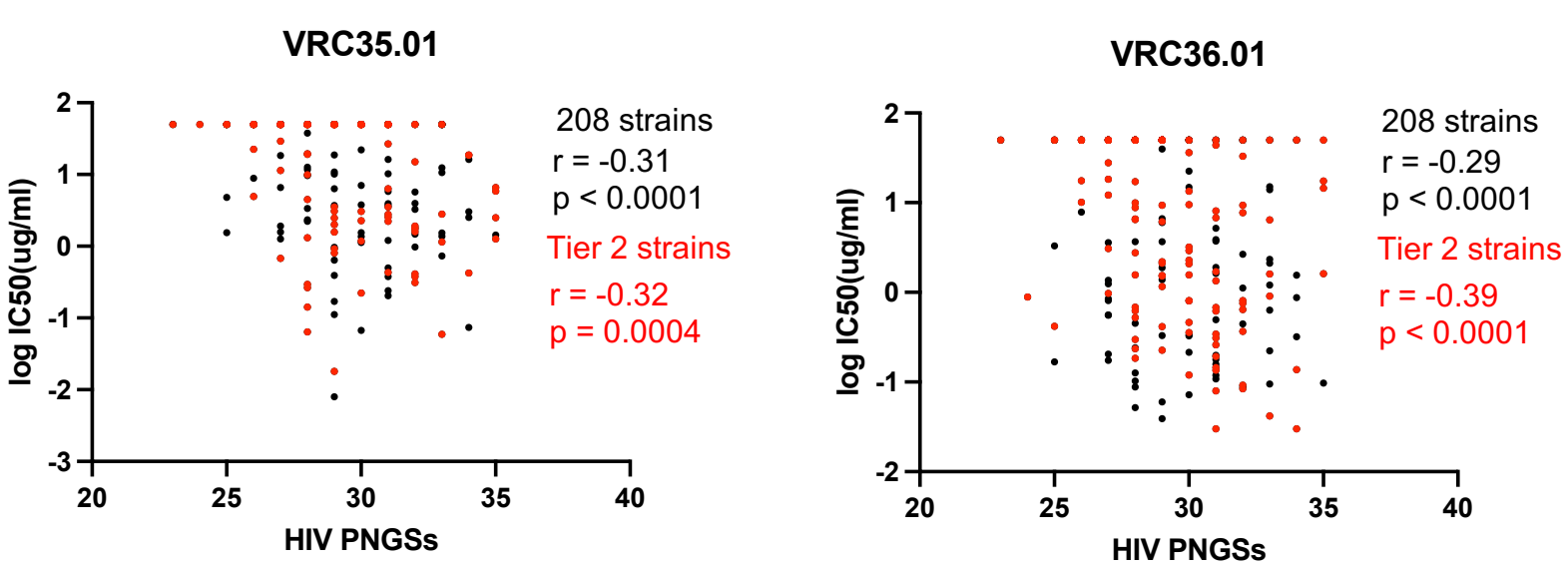

Supplementary Figure 2. VRC35 and VRC36 target HIV-Env glycan-V3 supersite. a Spearman rank correlations between IC50s of VRC35, VRC36, and other HIV-1-specific bnAbs targeting different epitopes in q 208-virus panel. Higher values and darker shades of red indicate higher similarity of neutralization patterns. Correlation results were used as input for generating the neutralization-based antibody clustering tree shown on the left. The intersection of the vertical dotted line with horizontal lines defines the different epitope clusters. VRC35 and VRC36 cluster among other glycan-V3 antibodies and most closely with 2G12. **b** Fold-change in neutralization potency of glycan-V3 mutants of HIV strain JRCSF.DB relative to wildtype. **c** Correlations between the number of predicted *N*-linked glycosylation sites (PNGSs) on HIV-1 Env and neutralization IC50s for VRC35 and VRC36 in 208-virus strains (black) and Tier 2 strains (red), respectively. Values are shown as log IC50 (μg/ml). Spearman's rank correlation was performed, yielding the r value and its associated rho-value.

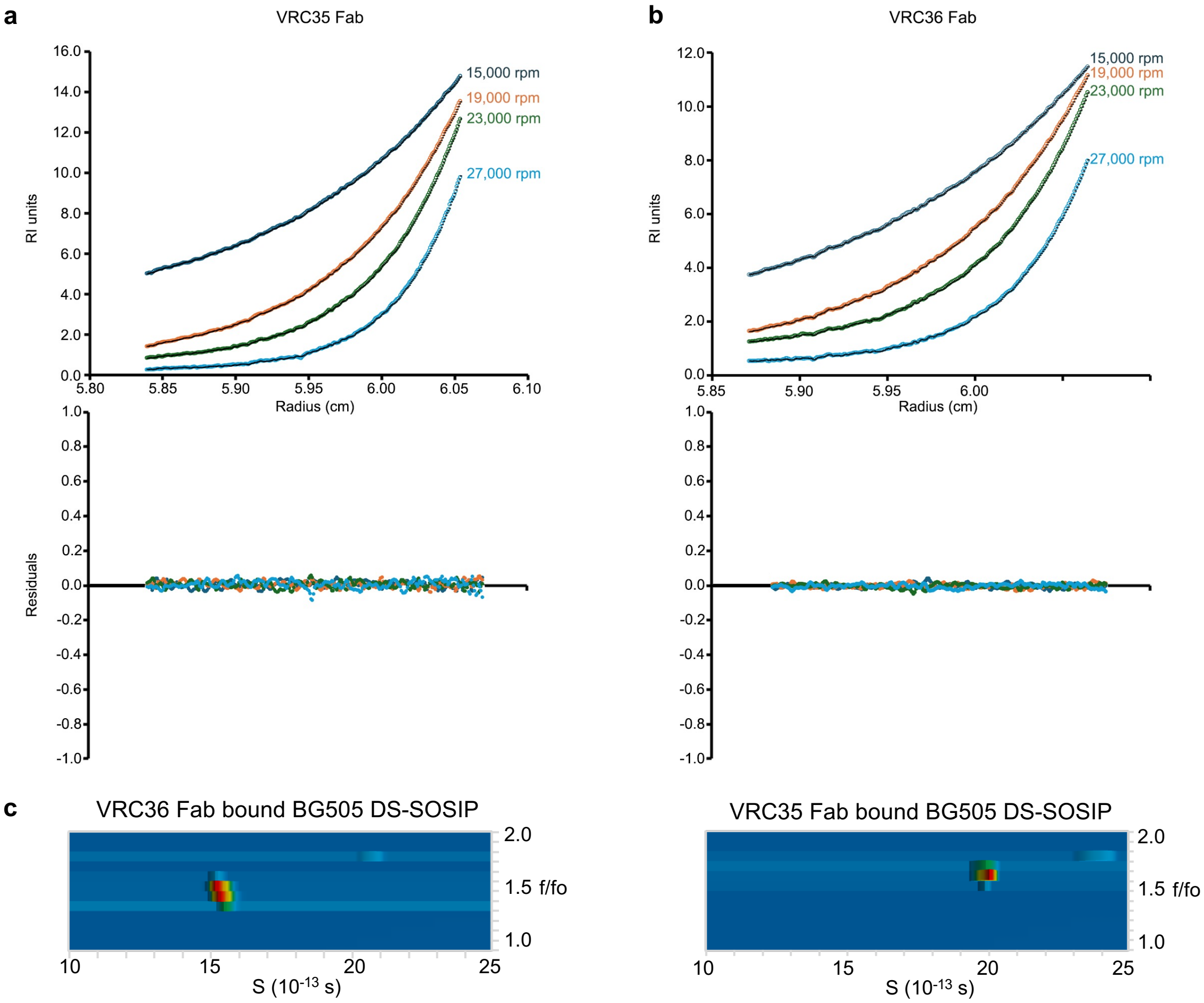

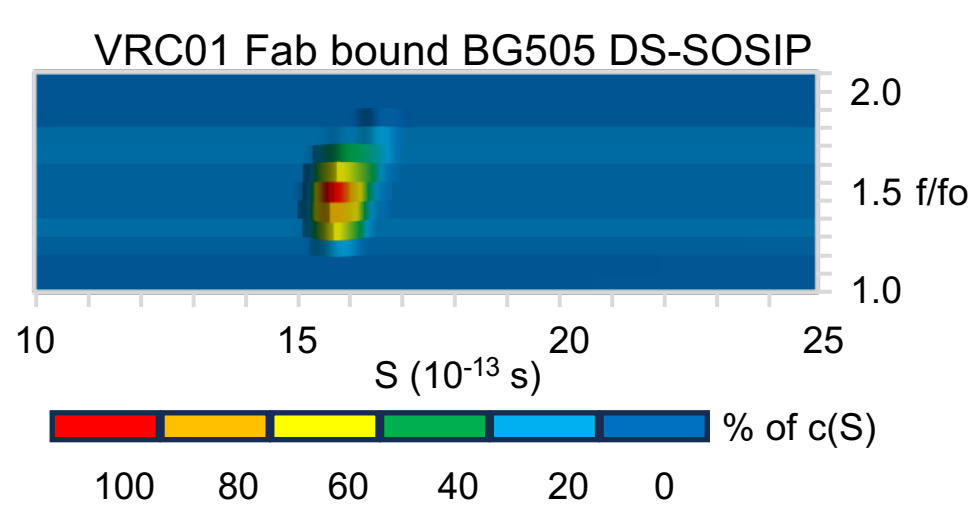

d

Fab	S (10^{-13} s)	M.W. (kDa)	N*
VRC35	20.1	890	11.9
VRC36	15.2	490	3.4
VRC01	15.8	460	2.8

Supplementary Figure 3. Analytical ultracentrifugation analysis of VRC35 Fab, VRC36 Fab, and their complex with BG505 DS-SOSIP.664. a-b Sedimentation equilibrium analytical ultracentrifugation measurements of VRC35 Fab and VRC36 Fab. Both Fabs are at the concentration of 2 mg/ml. The colored rings are the raw data, and the black dots are the fitted data to monomeric dispersed molecule. Samples are run for four speeds, 15,000 rpm, 19,000 rpm, 23,000 rpm and 27,000 rpm, respectively. **c** Analytical ultracentrifugation sedimentation velocity measurements of VRC35 Fab, VRC36 Fab, and VRC01 Fab bound to BG505 DS-SOSIP.664. Three-dimensional plots of sedimentation coefficient distributions (S, f/fo and c(S)) are shown. **d** The binding thermodynamic parameters together with the stoichiometries of binding are summarized in the table. *Stoichiometry, N, expressed as the number of Fab per Trimer.

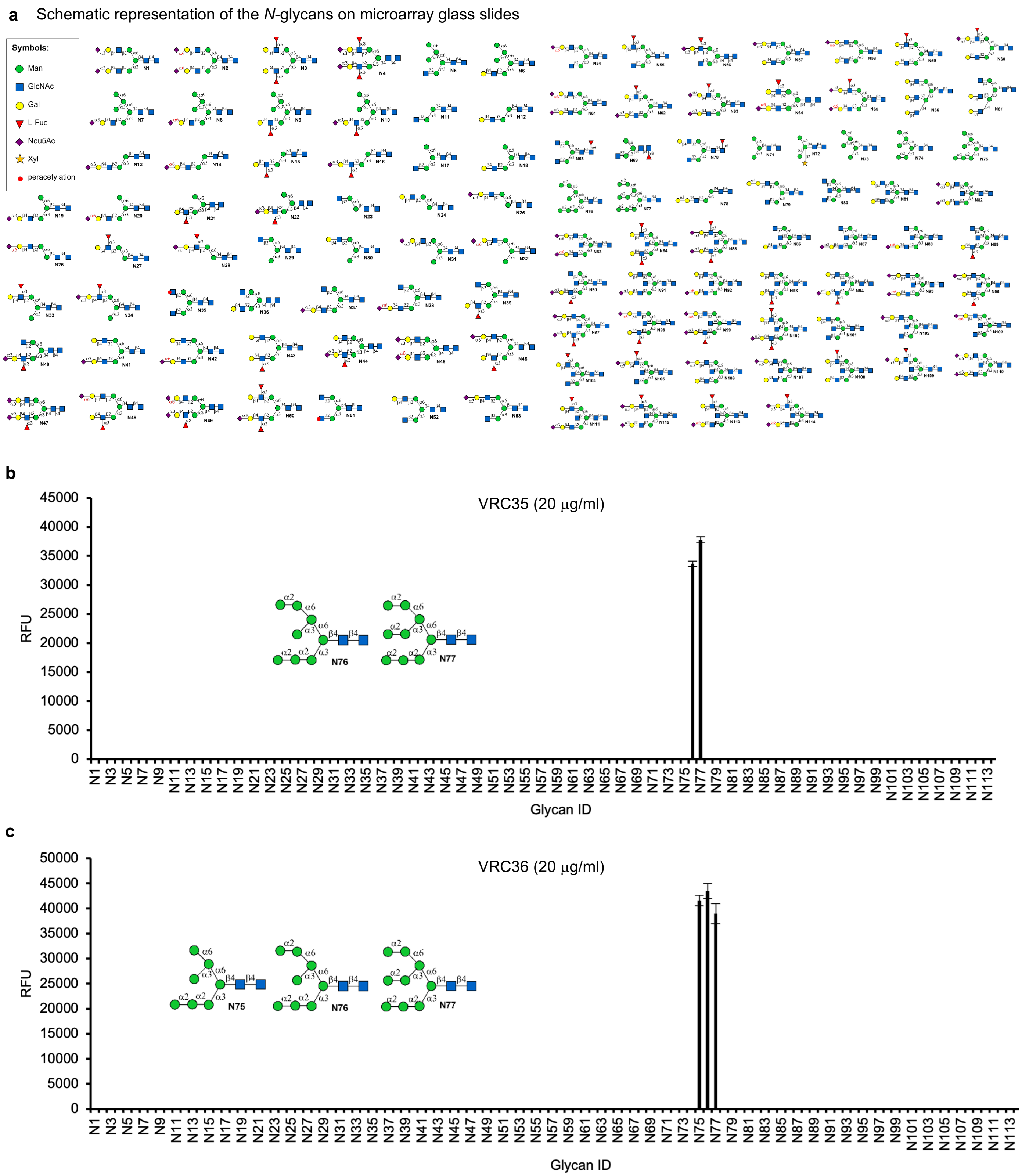

Supplementary Figure 4. *N*-glycan binding profiles of VRC35 and VRC36. a Schematic representation of the *N*-glycans in microarray assay. **b** VRC35 binds to high mannose *N*-glycans N76 (Man_8) and N77 (Man_9). **c** VRC36 binds to high mannose *N*-glycans N75 (Man_7), N76 (Man_8), and N77 (Man_9). All glycans contain dual α2 linked mannose residues on the α3 arm of the glycan.

a Polyreactivity profiles

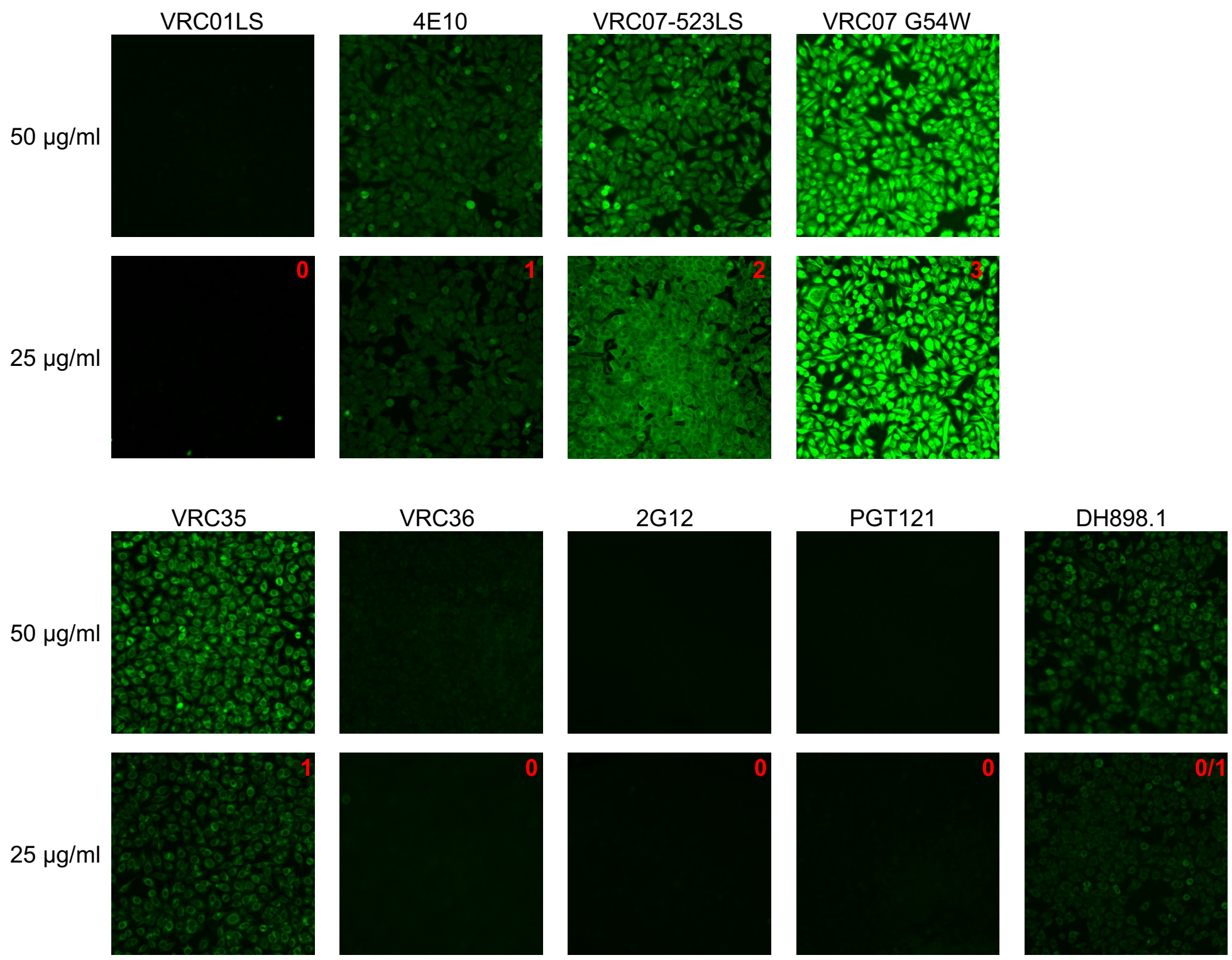

b Cardiolipin binding analysis

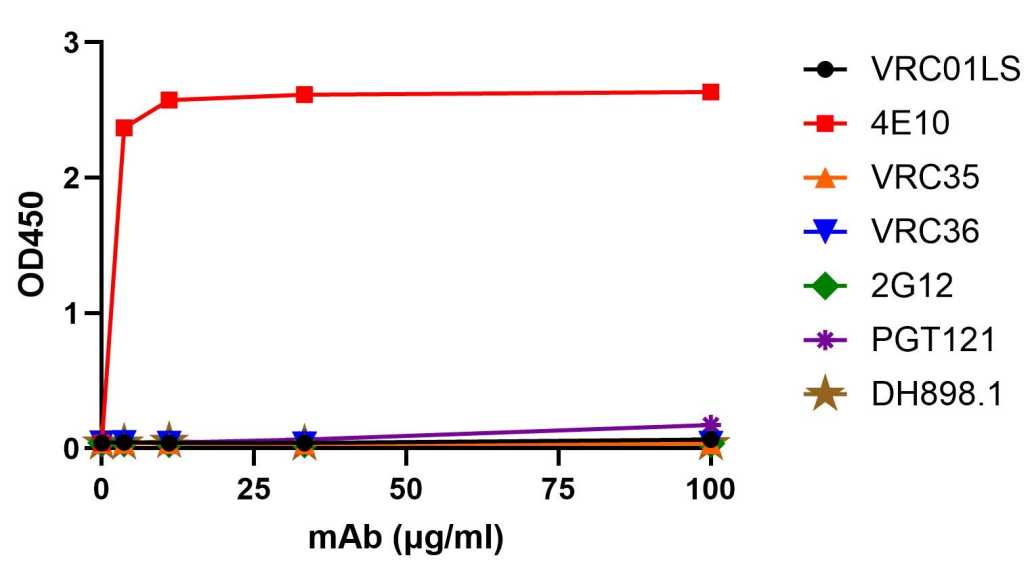

Supplementary Figure 5. Polyreactivity profiles of VRC35 and VRC36. a Representative HEp-2 test images of VRC35 and VRC36. As expected, negative control antibody VRC01LS didn't exhibit polyreactivity to HEp-2 cells (scaled as 0) when tested at at 25 µg/ml. In contrast, at the same concentration, antibodies 4E10, VRC07-523LS, and VRC07 G54W showed polyreactivity with scores of 1, 2, and 3, respectively. Duplicate tests revealed mild autoreactivity for VRC35 and DH898.1 antibodies, whereas VRC36, 2G12, and PGT121 did not show detectable binding to HEp-2 cells. Each antibody was tested at 50 and 25 µg/ml. A polyreactivity scale for 0/1 indicates a level between 0 and 1. **b** Negative cardiolipin binding of VRC35 and VRC36. Antibodies 4E10 and VRC01LS were used as positive and negative controls, respectively. Data points represent mean values from two independent experiments for VRC35, VRC36, 2G12, PGT121, and DH898.1, none of which showed detectable binding to cardiolipin.

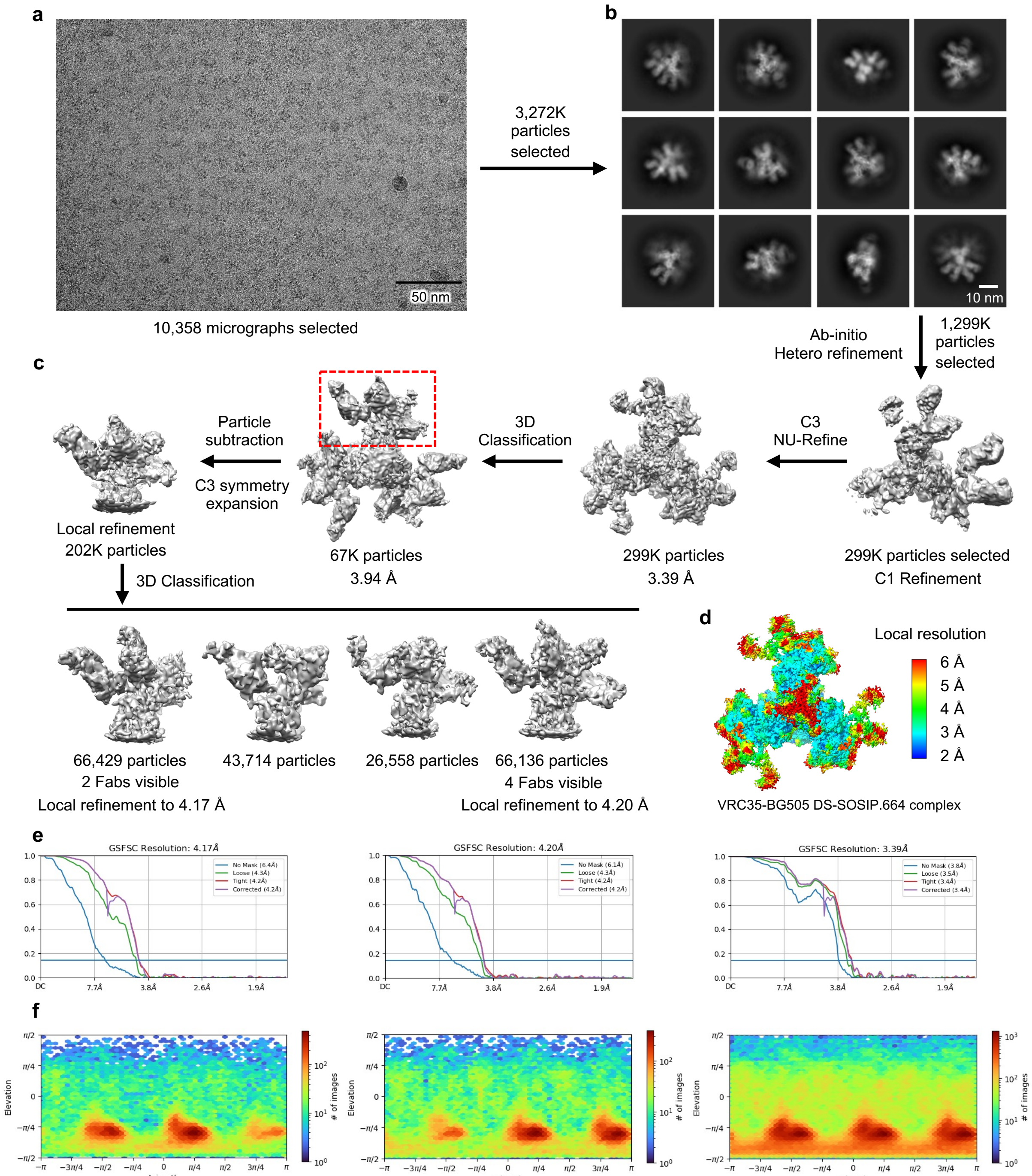

Supplementary Figure 6. Image processing of the VRC35 Fab in complex with BG505 DS-SOSIP.664 dataset. a A representative raw micrograph of cryo-EM data. **b-c** Workflow of image processing of VRC35 in complex with BG505 DS-SOSIP.664 particles (See Materials and Methods for details). **d** The local resolution of the VRC35-BG505 DS-SOSIP.664 map is shown. Resolution estimation was generated through cryoSPARC using an FSC cutoff of 0.143. **e** The gold-standard Fourier shell correlation resulted in a resolution of 4.17 Å local map of two VRC35 Fabs visible, 4.2 Å local map for four VRC35 Fabs visible, and 3.39 Å the overall map using non-uniform refinement with C3 symmetry for VRC35-BG505 DS-SOSIP.664 complex. **f** The orientation of all particles used in the final refinement are shown as a heatmap for two VRC35 Fabs visible local map, four VRC35 Fabs visible local map, and VRC35-BG505 DS-SOSIP.664 complex.

a ITC of BG505 DS-SOSIP.664 with VRC35 Fab

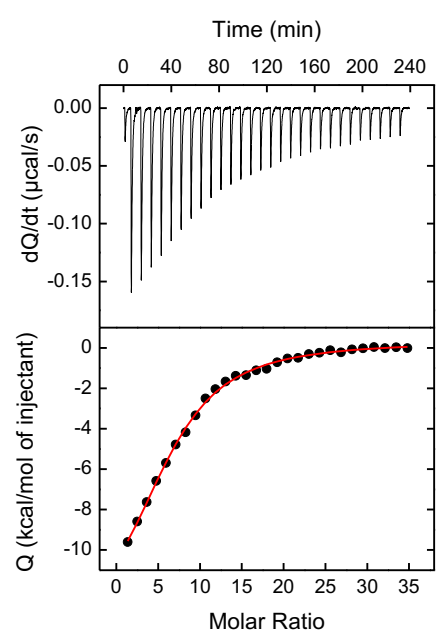

VRC35 Fab	
K_D (nM)	1,450 ± 120
ΔG (kcal/mol)	-8.0 ± 0.1
ΔH (kcal/mol)	-15.1 ± 0.7
-TΔS (kcal/mol)	+7.1 ± 0.4
N*	11.1 ± 0.3

b ITC of BG505 DS-SOSIP.664 with VRC36 Fab

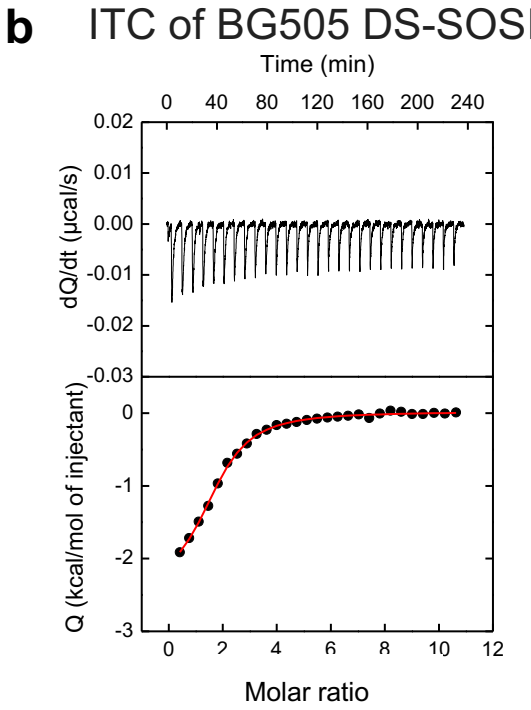

VRC36 Fab	
K_D (nM)	370 ± 28
ΔG (kcal/mol)	-8.8 ± 0.1
ΔH (kcal/mol)	-2.5 ± 0.1
-TΔS (kcal/mol)	-6.3 ± 0.3
N*	2.8 ± 0.1

Supplementary Figure 7. Isothermal titration calorimetry of VRC35 Fab and VRC36 Fab with HIV-1 Env BG505 DS-SOSIP.664. a-b ITC data for titrations of BG505 DS-SOSIP.664 in complex with VRC35 Fab and VRC36 Fab. The binding thermodynamic parameters together with the stoichiometries of binding are summarized in the table. *Stoichiometry, N, expressed as the number of Fab per Trimer.

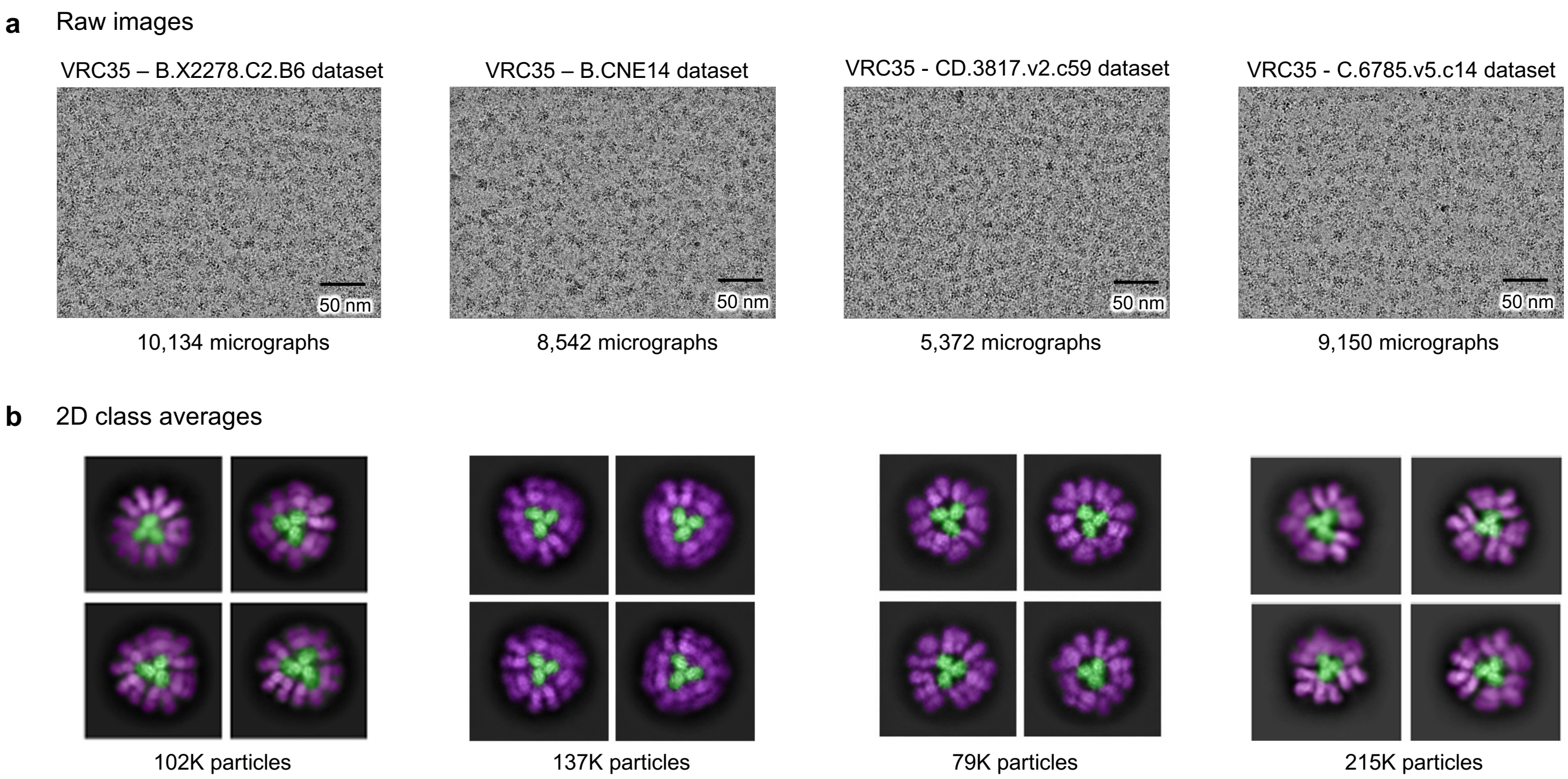

c

IC50

Virus ID	Clade	VRC35	PGT121	PGT128	PGT135	2G12	BG18	10-1074	DH270
3817.v2.c59	CD	0.018	22.2000	0.017	>50	4.2100	0.190	0.248	0.736
6785.V5.C14	C	0.064	0.050	0.016	0.335	>50	0.0050	0.031	0.247
X2278.C2.B6	B	0.313	0.026	0.006	>50	0.084	0.003	0.032	0.855
CNE14	B	0.067	0.010	0.014	0.117	>50	0.0040	0.011	0.066
BG505.W6M.C2	A	>50	0.051	0.048	>50	>50	>50	>50	>50
BG505.T332N	A	0.329	0.049	0.015	1.5600	0.491	0.0020	0.071	0.202

<0.01 | 0.01-0.1 | 0.1-1.0 | 1.0-10 | 10-50 | >50
IC_{50} (µg/ml)

IC80

Virus ID	Clade	VRC35	PGT121	PGT128	PGT135	2G12	BG18	10-1074	DH270
3817.v2.c59	CD	0.049	>50	0.055	>50	29.7000	0.923	1.3700	2.7600
6785.V5.C14	C	0.374	0.108	0.047	0.499	>50	0.012	0.067	0.525
X2278.C2.B6	B	0.752	0.072	0.023	>50	0.485	0.0060	0.097	4.5800
CNE14	B	0.474	0.024	0.049	0.404	>50	0.0100	0.028	0.153
BG505.W6M.C2	A	>50	0.165	0.569	>50	>50	>50	>50	>50
BG505.T332N	A	1.2700	0.107	0.036	22.0000	2.4000	0.0060	0.199	0.711

<0.01 | 0.01-0.1 | 0.1-1.0 | 1.0-10 | 10-50 | >50
IC_{80} (µg/ml)

Supplementary Figure 8. Structural validation of VRC35 binding to genetically diverse HIV-1 Env trimers. a Representative raw micrographs used for cryo-EM analysis of VRC35 in complex with B.X2278.C2.B6, B.CNE14, CD.3817.v2.c59, and C.6785.v5.c14 Env trimers. **b** Representative 2D class averages of VRC35-Env complexes. VRC35 Fabs are shown in purple, and HIV-1 Env trimers are shown in green. The class averages reveal four VRC35 Fab densities per Env protomer, corresponding to a total stoichiometry of twelve Fabs per Env trimer, consistent with the VRC35-BG505 DS-SOSIP.664 structure. **c** Neutralization activity of VRC35 and reference glycan-V3 antibodies against the HIV-1 strains selected for structural analysis. Values are shown as IC_{50} (µg/ml) and IC_{80} (µg/ml).

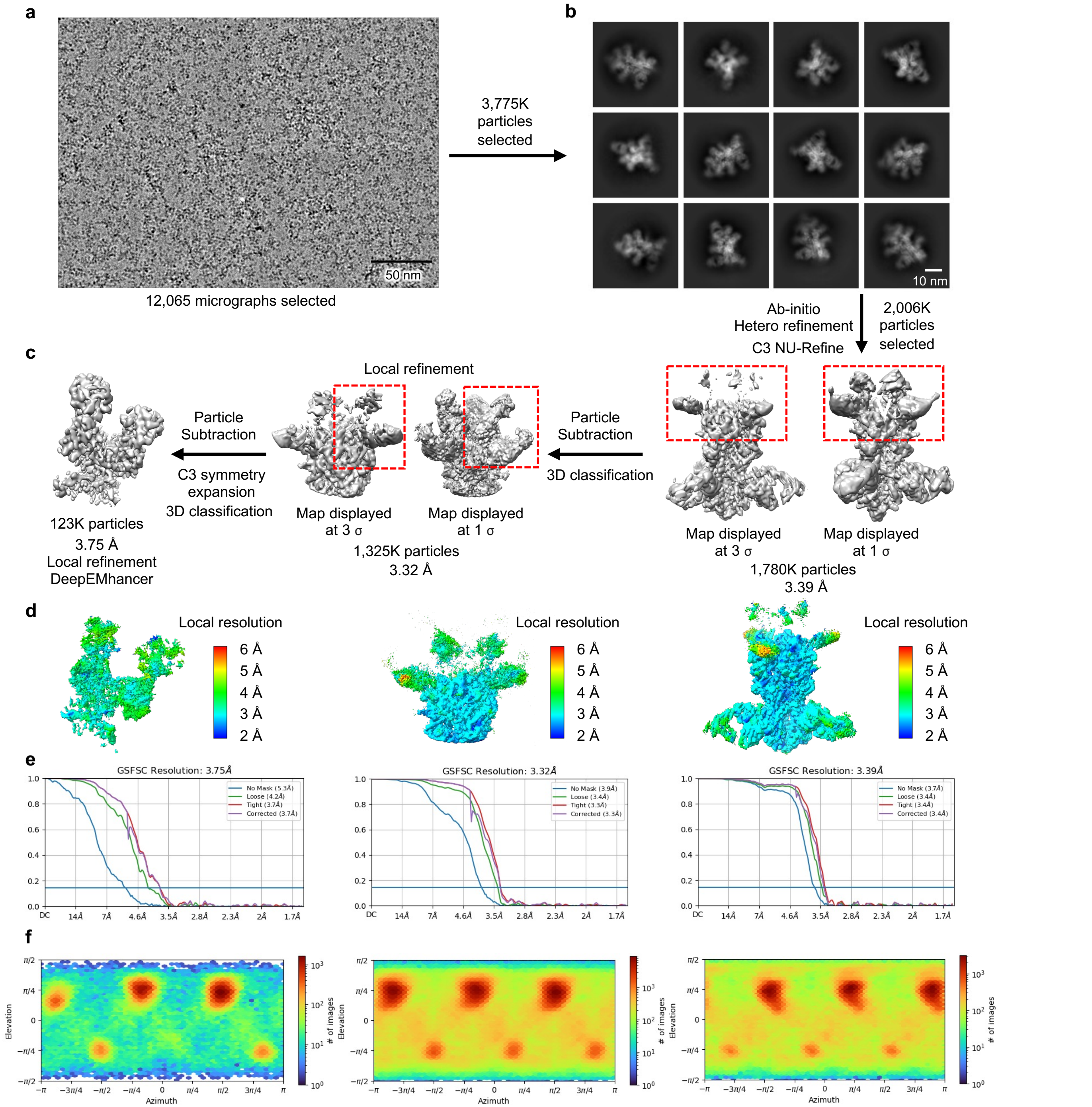

Supplementary Figure 9. Image processing of the VRC35 Fab and MEDI8852 Fab in complex with influenza H3N2 Victoria 2011 hemagglutinin dataset. a A representative raw micrograph of cryo-EM data. **b-c** Workflow of image processing of VRC35 Fab and MEDI8852 Fab in complex with influenza H3N2 Victoria 2011 hemagglutinin head (See Materials and Methods for details). **d** The local resolution of the dimerized VRC35 Fabs, VRC35 Fabs, VRC35-MEDI8852-H3N2 Vic11 HA cryo-EM maps were shown. Resolution estimation was generated through cryoSPARC using an FSC cutoff of 0.143. **e** The gold-standard Fourier shell correlation resulted in a resolution of 3.75 Å for dimerized VRC35 Fabs, 3.32 Å for the VRC35 Fabs, and 3.23 Å for the VRC35-MEDI8852-H3N2 Vic11 HA maps. **f** The orientation of all particles used in the final refinement are shown as a heatmap.

N-glycosylation profiling of influenza virus H3N2 Victoria 2011 hemagglutinin

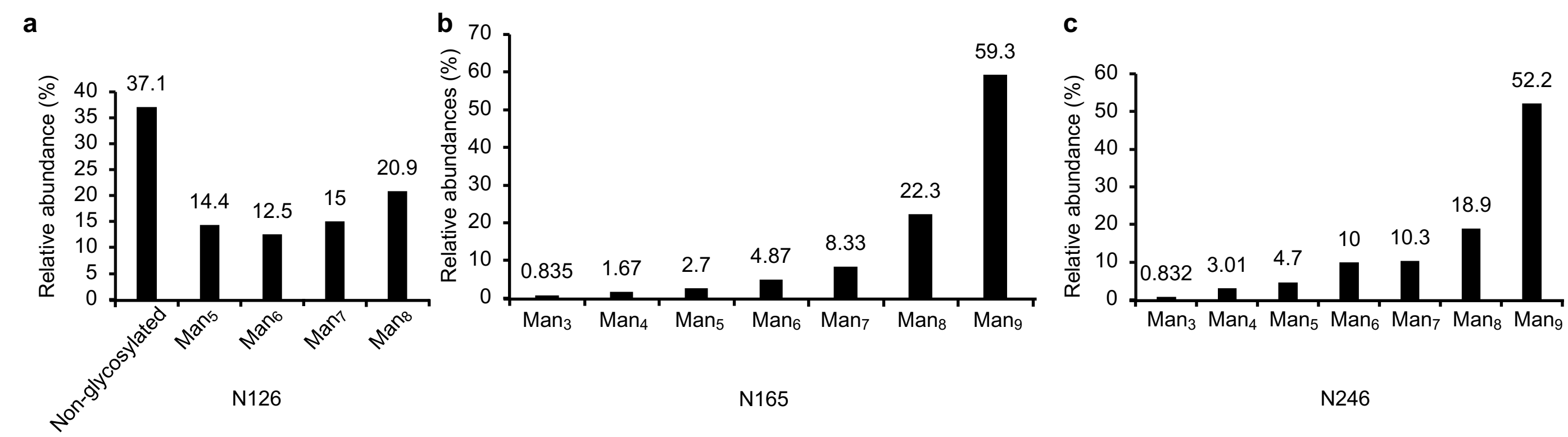

N-glycosylation profiling of SARS-CoV-2 LP.8.1.9 spike

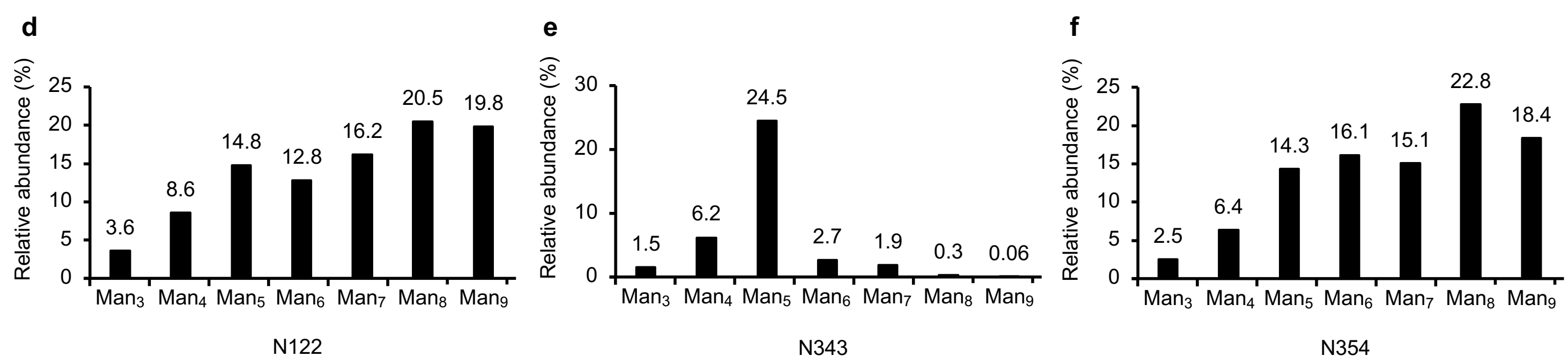

Supplementary Figure 10. Relative abundance of *N*-glycosylation profiling of glycan sites on Influenza virus H3N2 Victoria 2011 hemagglutinin and SARS-CoV-2 LP.8.1.9 spike. *N*-glycosylation were observed on H3N2 Vic11 N126, N165 and N246. All three sites showed high mannose glycans. **a** High mannose type N-glycans Man_5, Man_6, Man_7, and Man_8 were observed on N126. Non-glycosylated peptides are predominant at this site. **b-c** High mannose type N-glycans were observed on N165 and N246. Man_9 is the predominant type of glycan at both sites. **d-f** Site-specific N-glycosylation profiling of SARS-CoV-2 LP.8.1.9 spike. N122 contains a mixed population of high-mannose glycans with Man8 as the predominant species. N343 is predominantly occupied by Man5, whereas N354 is enriched in high-mannose glycans, including Man8 and Man9.

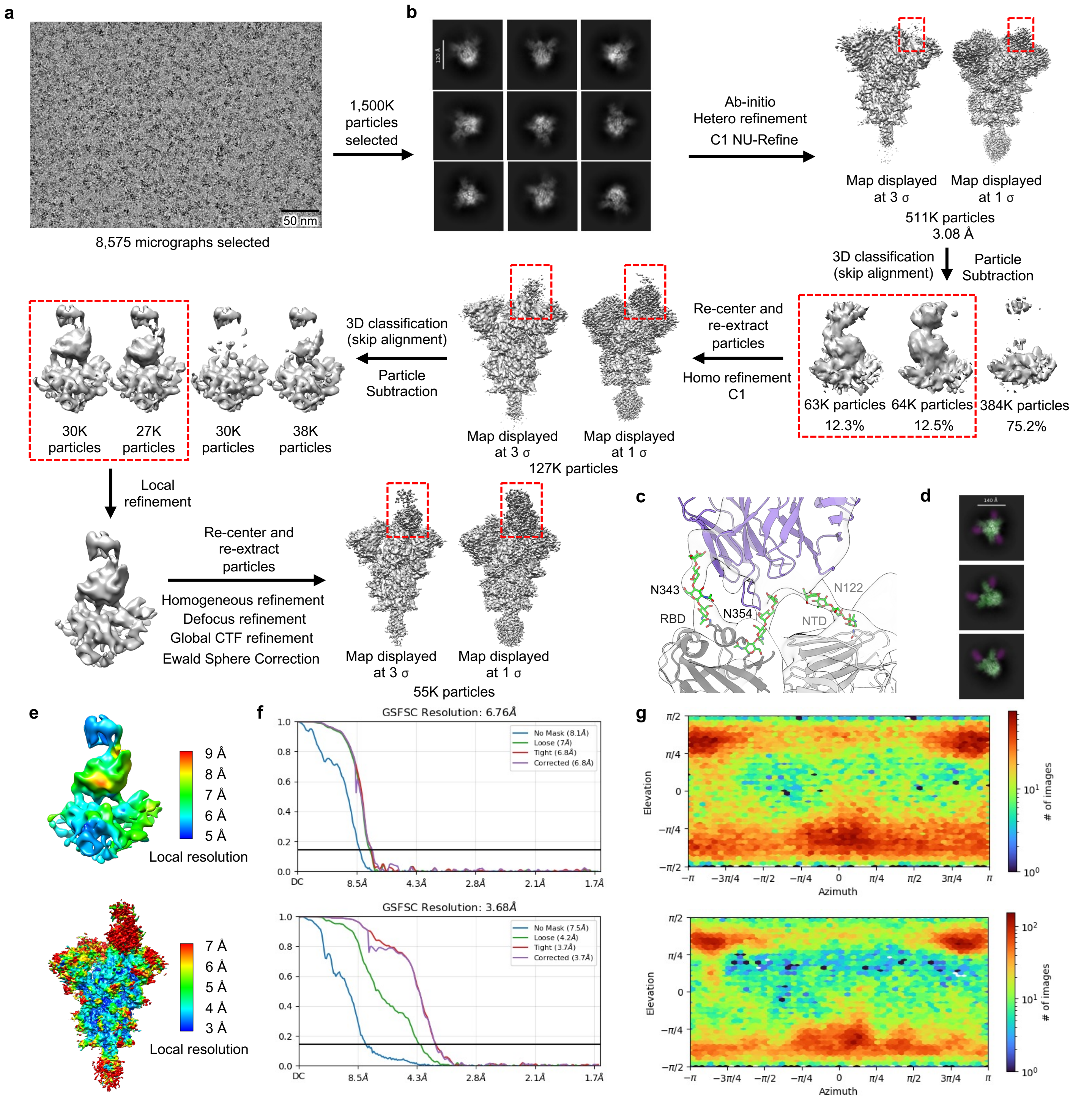

Supplementary Figure 11. Image processing of the VRC35 Fab in complex with SARS-CoV-2 LP.8.1.9 spike dataset. a A representative raw micrograph of cryo-EM dataset. **b** Workflow of image processing of VRC35 Fab in complex with SARS-CoV-2 LP.8.1.9 spike (See Materials and Methods for details). **c** Local density of glycan region for N343 and N354 in RBD and N122 in NTD from the final local refinement cryo-EM density map. **d** 2D class averages revealed weak VRC35 Fab density. Spike region was highlighted in green and VRC35 Fab was highlighted in purple. **e** The local resolution of VRC35 Fab with three glycans and VRC35-SARS-CoV-2 LP.8.1.9 spike cryo-EM map were shown. Resolution estimation was generated through cryoSPARC using an FSC cutoff of 0.143. **f** The gold-standard Fourier shell correlation resulted in a resolution of 6.76 Å for VRC35-glycan local cryo-EM map and 3.68 Å for VRC35-SARS-CoV-2 LP.8.1.9 spike overall cryo-EM map. **g** The orientation of all particles used in the final refinement are shown as a heatmap.

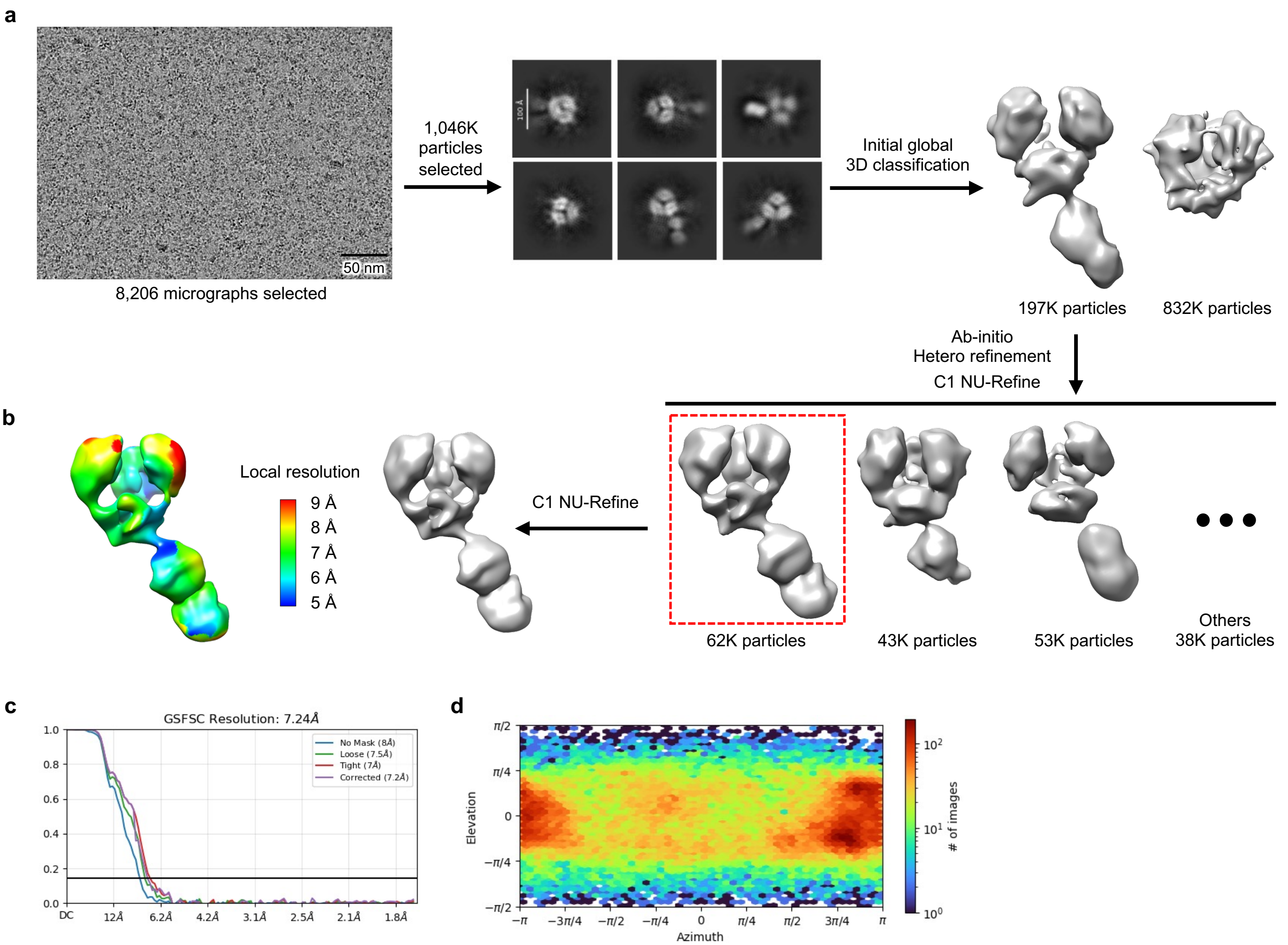

Supplementary Figure 12. Image processing of the VRC35 Fab in complex with LASV Josiah GPC dataset. a A representative raw micrograph of cryo-EM dataset. Workflow of image processing of VRC35 Fab in complex with LASV Josiah GPC (See Materials and Methods for details). **b** The local resolution of VRC35 Fab in complex with LASV Josiah GPC cryo-EM map was shown. Resolution estimation was generated through cryoSPARC using an FSC cutoff of 0.143. **c** The gold-standard Fourier shell correlation resulted in a resolution of 6 Å for VRC35-LASV Josiah GPC cryo-EM map. **d** The orientation of all particles used in the final refinement are shown as a heatmap.

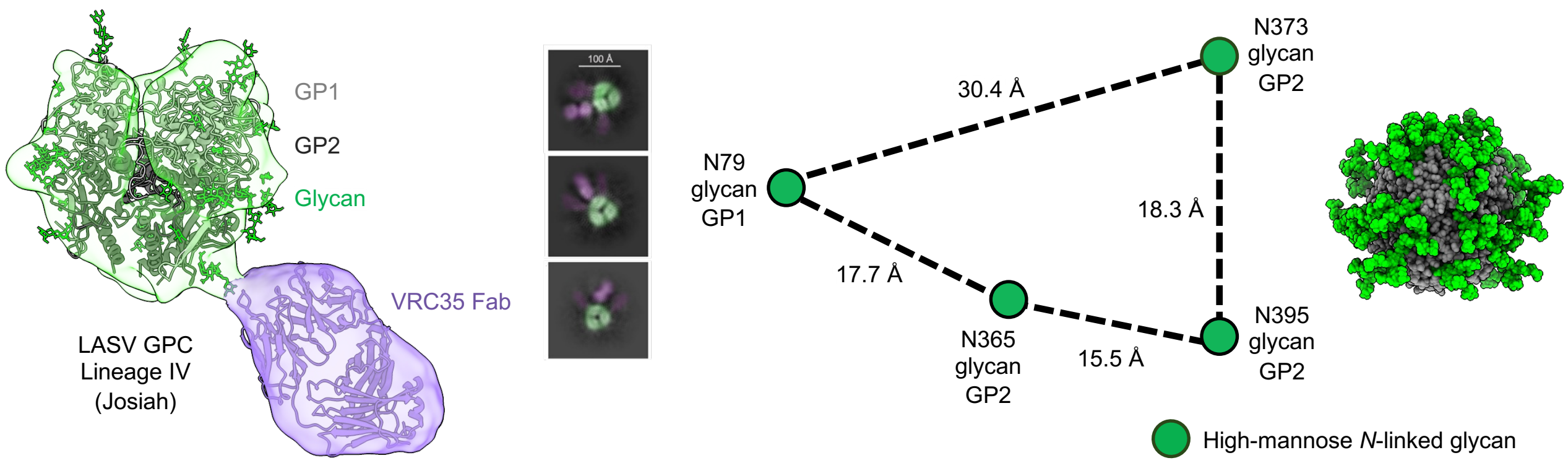

Supplementary Figure 13. Cryo-EM structure of LASV GPC (Josiah, lineage IV) in complex with VRC35 Fab. Cryo-EM reconstruction of LASV GPC (Josiah, lineage IV) reveals one dominant VRC35 Fab density and additional weak Fab densities in 2D class averages that were not resolved in the final 3D reconstruction. The glycan matrix highlights *N*-linked oligomannose glycans that may be accessible to VRC35. A fully glycosylated model of LASV GPC (Josiah, Lineage IV) illustrates glycan density and was generated using the GLYCAM-Web prediction tool.

a In vitro microneutralization assay showing VRC35 neutralizes influenza H3 strains, while VRC36 exhibits weak neutralization of influenza H3 strains

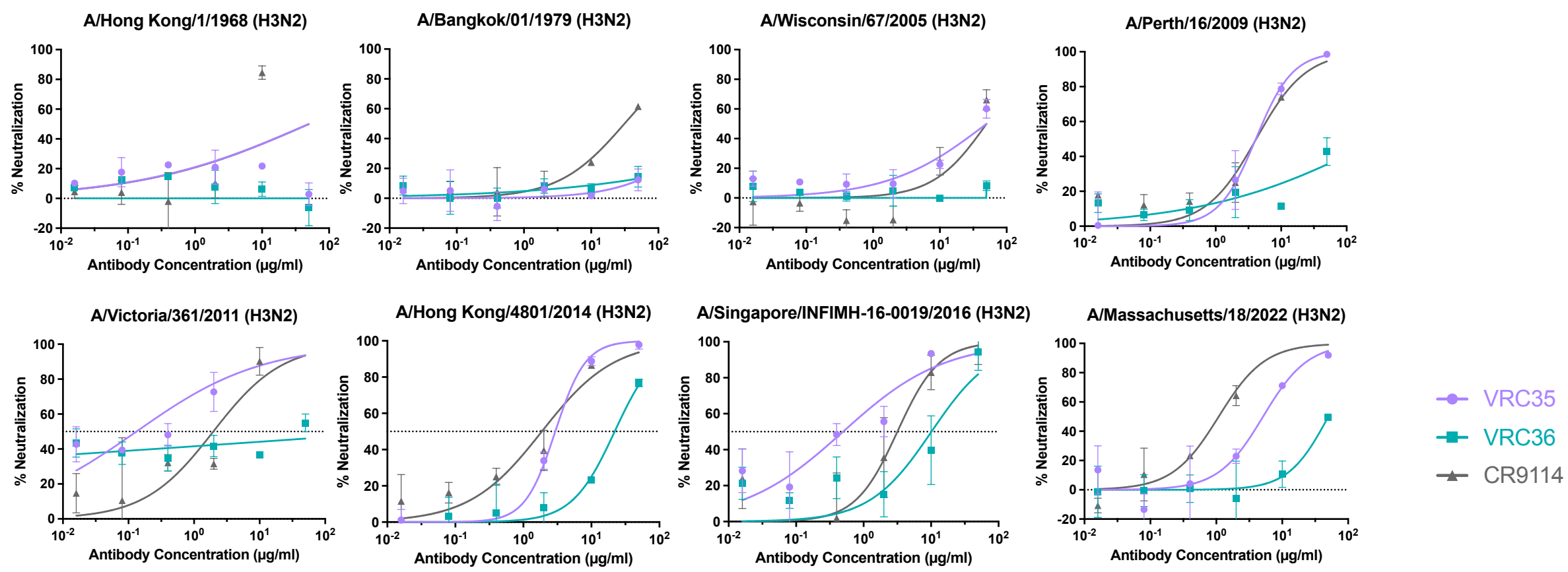

IC50 (µg/ml)	A/Hong Kong/1968 (H3N2)	A/Bangkok/1979 (H3N2)	A/Wisconsin/2005 (H3N2)	A/Perth/2009 (H3N2)	A/Victoria/2011 (H3N2)	A/Hong Kong/2014 (H3N2)	A/Singapore/2016 (H3N2)	A/Massachusetts/2022 (H3N2)
VRC35	>50	>50	34.54	4.10	0.13	2.95	0.50	5.13
VRC36	>50	>50	>50	>50	>50	22.03	10.14	>50
CR9114	4.93	31.34	28.77	4.16	1.97	1.87	3.17	1.09

0.1-1.0	1.0-10	10-50	>50	Color scale of IC_{50} (µg/ml)

b Reporter virus assay of VRC35 against diverse influenza strains

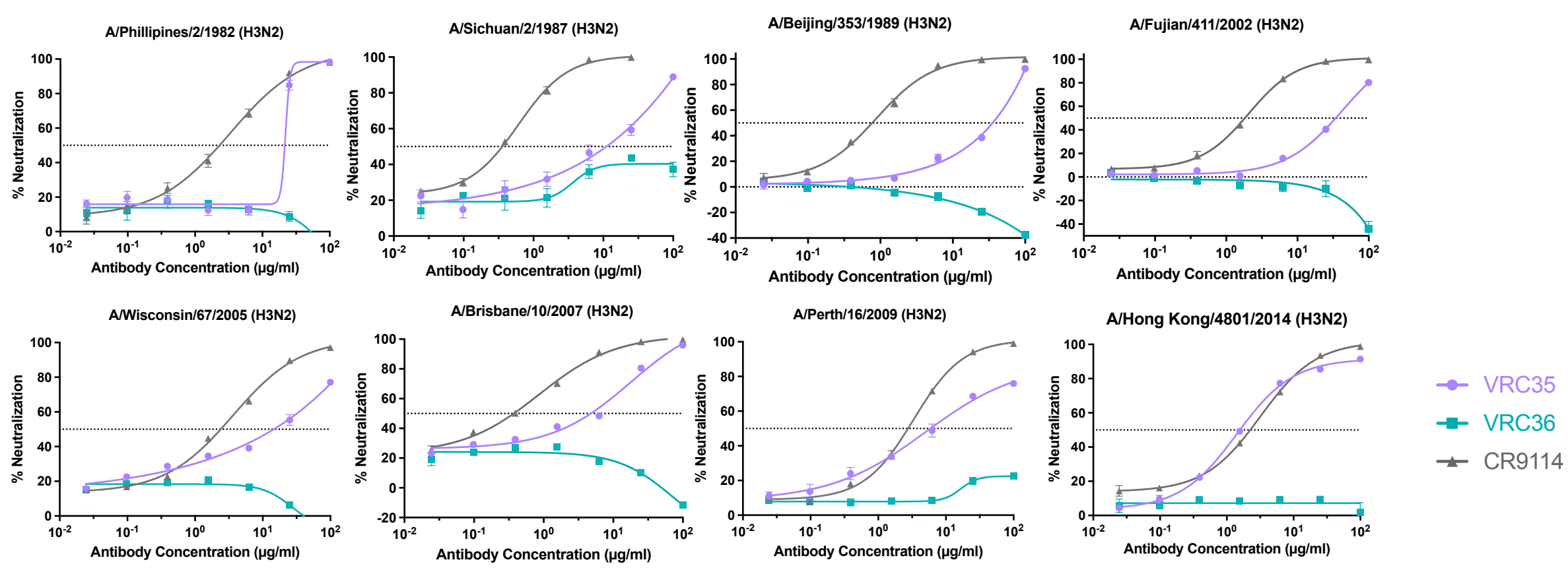

c VRC35 and VRC36 did not neutralize SARS-CoV-2 variants

IC50 (µg/ml)	WA.1	BA.2.86	JN.1	KP.3	KP.3.1.1	LF.7.2.1	LP.8.1.9	NB.1.8.1	BA.3.2
VRC35	>100	>100	>100	>100	>100	>100	>100	>100	>100
VRC36	>100	>100	>100	>100	>100	>100	>100	>100	>100

d VRC35 and VRC36 did not neutralize LASV lineages

IC50 (µg/ml)	Josiah (IV)	AV (IV)	GA391 (III)	803213 (II)	TOGO (2016)	Pinneo (I)
VRC35	>100	>100	>100	>100	>100	>100
VRC36	>100	>100	>100	>100	>100	>100

Supplementary Figure 14. VRC35 neutralize influenza but not LASV and SARS-CoV-2. a VRC35 neutralizes several influenza H3N2 strains, while VRC36 exhibits weak neutralization of influenza H3 strains in an in vitro microneutralization assay. **b** VRC35 neutralizes multiple H3N2 influenza strains in reporter virus assays using an independent virus panel spanning multiple decades of H3N2 evolution. Similar results were observed across both assay platforms. **c** VRC35 and VRC36 did not neutralize SARS-CoV-2 variants. **d** VRC35 and VRC36 did not neutralize LASV lineages.

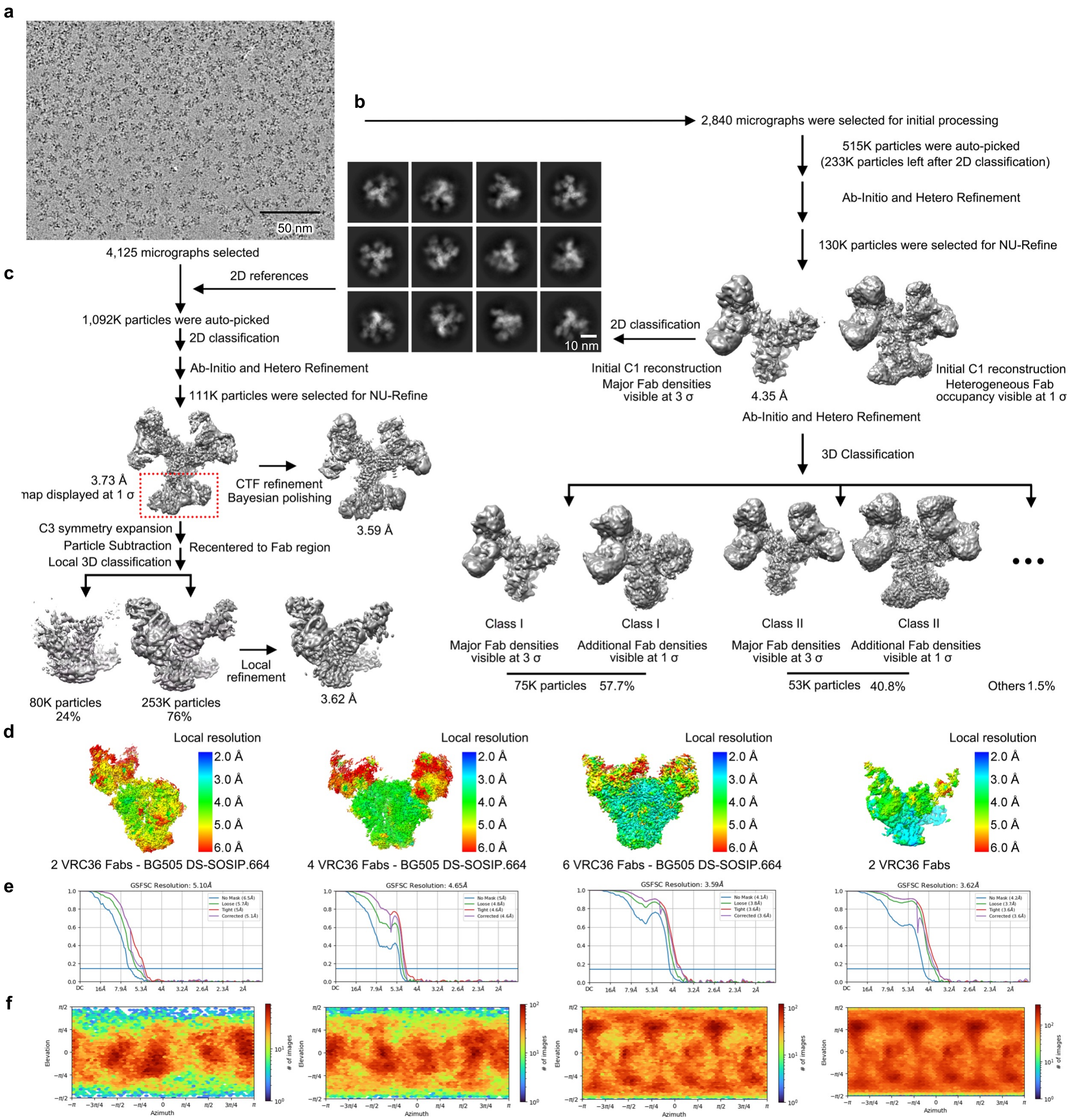

Supplementary Figure 15. Image processing of the VRC36 Fab in complex with BG505 DS-SOSIP.664 dataset. a A representative raw micrograph of cryo-EM data. **b-c** Workflow of image processing of VRC36 in complex with BG505 DS-SOSIP.664 particles (See Materials and Methods for details). Initial reconstructions revealed heterogeneous Fab occupancy, prompting additional rounds of particle classification. Distinct particle populations corresponding to 2-, 4-, and 6-Fab occupancy states were identified through Ab-initio Reconstruction, Heterogeneous Refinement, C3 symmetry expansion, particle subtraction, and focused local 3D classification. Contour levels shown in the workflow are provided for visualization purposes only and were not used to assign Fab stoichiometry. **d** The local resolutions of 2 VRC36 Fabs-BG505 DS-SOSIP.664 map, 4 VRC36-BG505 DS-SOSIP.664 map, 6 VRC36-BG505 DS-SOSIP.664 map and VRC36 local map were shown. Resolution estimation were generated through cryoSPARC using an FSC cutoff of 0.143. **e** The gold-standard Fourier shell correlation resulted in a resolution of 5.1 Å for the overall map using non-uniform refinement with C1 symmetry for 2 VRC36 Fabs-BG505 DS-SOSIP.664 complex, 4.65 Å for the overall map using non-uniform refinement with C1 symmetry for 4 VRC36 Fabs-BG505 DS-SOSIP.664 complex, 3.59 Å for the overall map using non-uniform refinement with C3 symmetry for 6 VRC36 Fabs-BG505 DS-SOSIP.664 complex, and 3.62 Å for the VRC36 dimerized Fab local density map. **f** The orientation of all particles used in the final refinement are shown as a heatmap.

Supplementary Table 1. Glycan-V3 antibody heavy chain and light chain genetic characteristics

Antibody	PDB	VH Gene	CDRH3 Sequence	VL Gene	CDRL3 Sequence
VRC35	9OM5	VH4-34	CARGRGTVATELAMW	VK2-24	CMQATQFPRTF
VRC36	9NPM	VH3-15	CVHLREKGDDW	VK3-11	CQQRGEWPFTF
QA013.2	7N65	VH3-7	CAREAYHLVYDDRIPRGNWFDPW	VL1-40	CQSYDTTLTASVF
PGT121	7UOJ	VH4-59	CARTLHGRRIYGIVAFNEWFTYFYMDVW	VL3-21	CHIWDSRVPTKWVF
PGT128	6OPA	VH4-39	CARFGGEVLRYTDWPKPAWVDLW	VL2-8	CGSLVGNWDVIF
PGT135	4JM2	VH4-39	CHRHHDVFMLVPIAGWFDVW	VK3-15	CQQYEEWPRTF
10-1074	7UCG	VH4-4 or VH4-59	CATARRGQRIYGVVSFGEFFYYYSMDVW	VL3-21	CHMWDSRSGFSWSF
BG18	6CH7	VH4-4	CARNAIRIYGVVALGEWFHYGMDVW	VL3-25	CQSSDTSDSYKMF
2G12	6N32	VH3-21	ARKGSDRLSDNDPFDA	VL1-5	QHYAGYSAT
DH270	6UM6	VH1-2	CTTGGWISLYYDSSYYPNFDHW	VL2-23	CCSFGGSATVVF
DH475	8D3A	VH3-23	CVKDSSTAWWDKPAASEFDFW	VL4-69	CQAWTADLHVF

Supplementary Table 2. IC50 values (μg/ml) of HIV-1 glycan-V3-specific bNAbs

<u>IC50</u>

Virus ID	Clade	N26-VRC35	N26-VRC36	CH0765-VRC41.01	PGT121	PGT128	PGT135	VRC24	N170-VRC29.03	2G12	BG18	10-1074	DH270
0260.v5.c36	A	0.978	0.446	0.361	0.059	0.052	0.855	>50	>50	>50	0.005	0.179	2.32
0330.v4.c3	A	2.48	9.36	>50	0.060	0.639	>50	>50	>50	1.06	0.510	0.054	>50
0439.v5.c1	A	18.8	0.137	>50	>50	1.84	0.805	>50	13.1	>50	>50	>50	>50
3365.v2.c20	A	>50	0.416	0.128	0.112	>50	0.109	>50	>50	>50	0.004	0.083	>50
3415.v1.c1	A	2.78	1.70	0.182	>50	0.010	0.369	>50	>50	1.46	0.025	>50	>50
3718.v3.c11	A	4.96	>50	>50	0.559	>50	>50	>50	>50	>50	>50	>50	>50
398-F1_F6_20	A	1.27	1.24	0.020	0.022	0.003	>50	5.00	1.48	29.6	0.0010	0.013	0.038
BB201.B42	A	1.58	0.557	0.038	0.009	0.004	>50	>50	>50	0.439	0.004	0.012	>50
BB539.2B13	A	>50	>50	>50	>50	>50	>50	>50	>50	>50	>50	>50	>50
BG505.W6M.C2	A	6.21	12.2	>50	0.051	0.048	>50	>50	>50	>50	>50	>50	>50
BI369.9A	A	0.499	0.179	1.84	0.028	0.032	0.114	>50	>50	1.68	0.004	0.050	0.407
BS208.B1	A	>50	>50	0.126	>50	>50	0.231	>50	>50	>50	>50	>50	>50
KER2008.12	A	>50	3.58	>50	0.553	1.53	>50	>50	>50	2.89	0.191	0.179	14.6
KER2018.11	A	>50	0.560	>50	>50	>50	>50	>50	>50	>50	>50	>50	>50
KNH1209.18	A	1.91	>50	0.074	0.012	0.007	0.083	0.017	0.332	1.28	0.003	0.013	0.085
MB201.A1	A	>50	3.68	>50	0.009	0.024	>50	0.277	>50	>50	>50	>50	>50
MB539.2B7	A	16.2	1.90	>50	>50	1.02	>50	>50	>50	>50	>50	>50	>50
MI369.A5	A	1.37	0.334	0.356	0.051	0.051	0.193	>50	0.701	4.53	0.005	0.059	0.501
MS208.A1	A	>50	0.168	1.71	>50	>50	0.696	>50	>50	>50	>50	>50	>50
Q23.17	A	6.60	1.36	0.076	0.011	0.015	9.84	0.159	0.057	>50	0.003	0.011	0.058
Q259.17	A	>50	>50	>50	>50	>50	>50	>50	>50	>50	>50	>50	>50
Q769.d22	A	>50	18.3	>50	>50	>50	>50	>50	>50	>50	>50	>50	>50
Q769.h5	A	>50	>50	>50	>50	>50	>50	>50	>50	>50	>50	>50	>50
Q842.d12	A	19.5	8.83	>50	0.022	0.029	>50	>50	8.92	>50	>50	>50	>50
QH209.14M.A2	A	>50	>50	>50	>50	>50	>50	>50	>50	>50	>50	>50	>50
RW020.2	A	1.31	>50	0.063	0.010	0.003	>50	6.98	0.061	>50	0.002	0.005	0.051
UG037.8	A	>50	>50	1.07	0.092	0.022	>50	>50	>50	>50	0.042	0.149	1.53
246-F3.C10.2	AC	>50	2.11	>50	>50	0.004	>50	>50	29.5	>50	>50	>50	>50
3301.V1.C24	AC	>50	>50	0.308	0.022	0.143	>50	>50	>50	>50	0.017	0.023	0.587
3589.V1.C4	AC	1.47	1.12	>50	>50	0.015	6.62	>50	5.88	1.96	18.7	>50	>50
6540.v4.c1	AC	>50	0.977	>50	>50	0.344	>50	>50	>50	>50	>50	>50	>50
6545.V4.C1	AC	>50	6.59	>50	>50	13.2	>50	>50	>50	>50	>50	>50	>50
0815.V3.C3	ACD	0.059	0.907	0.247	0.032	0.027	>50	0.442	5.59	>50	1.55	0.065	0.635
6095.V1.C10	ACD	>50	0.088	>50	1.98	3.77	30.0	>50	3.87	>50	>50	>50	>50
3468.V1.C12	AD	2.22	>50	38.2	0.076	9.62	>50	>50	>50	>50	0.103	0.012	>50
Q168.a2	AD	>50	13.4	>50	>50	>50	>50	>50	>50	>50	>50	>50	>50
Q461.e2	AD	18.6	>50	>50	>50	5.20	>50	>50	>50	>50	>50	>50	>50
620345.c1	AE	>50	>50	>50	>50	>50	>50	>50	>50	>50	>50	>50	>50
BJOX009000.02.4	AE	26.8	0.260	>50	0.751	0.012	>50	>50	>50	>50	>50	>50	>50
BJOX010000.06.2	AE	>50	1.34	>50	>50	1.01	>50	>50	>50	>50	>50	>50	>50
BJOX025000.01.1	AE	3.52	6.16	>50	>50	>50	>50	>50	>50	>50	>50	>50	>50
BJOX028000.10.3	AE	1.13	22.4	>50	>50	0.016	>50	>50	>50	2.58	7.24	>50	>50
C1080.c3	AE	29.1	>50	>50	>50	0.053	>50	>50	>50	>50	>50	>50	>50
C2101.c1	AE	>50	3.20	>50	>50	0.017	>50	>50	>50	>50	>50	>50	>50
C3347.c11	AE	>50	1.54	>50	>50	0.007	>50	>50	>50	>50	>50	>50	>50
C4118.09	AE	>50	>50	>50	>50	>50	>50	>50	>50	>50	>50	>50	>50
CM244.ec1	AE	>50	27.9	>50	>50	0.011	>50	>50	>50	>50	>50	>50	>50
CNE3	AE	>50	0.848	>50	>50	>50	>50	>50	>50	>50	>50	>50	>50
CNE5	AE	>50	6.79	>50	>50	0.075	>50	>50	>50	>50	>50	>50	>50
CNE55	AE	>50	0.522	>50	>50	>50	>50	>50	>50	>50	>50	>50	>50
CNE56	AE	9.65	0.451	>50	>50	1.45	>50	>50	>50	>50	>50	>50	>50
CNE59	AE	>50	1.52	>50	>50	>50	>50	>50	>50	>50	>50	>50	>50
CNE8	AE	>50	1.88	>50	>50	0.011	>50	>50	>50	>50	>50	>50	>50
M02138	AE	>50	0.215	>50	>50	>50	>50	>50	>50	>50	>50	>50	>50
R1166.c1	AE	6.57	>50	>50	>50	>50	>50	>50	>50	>50	>50	>50	>50
R2184.c4	AE	19.1	2.76	>50	>50	0.654	>50	>50	>50	>50	>50	>50	>50
R3265.c6	AE	2.00	>50	>50	>50	>50	>50	>50	>50	>50	>50	>50	>50
TH023.6	AE	>50	>50	>50	>50	2.87	>50	>50	>50	>50	>50	>50	>50
TH966.8	AE	>50	>50	>50	>50	0.017	>50	>50	>50	>50	>50	>50	>50
TH976.17	AE	>50	>50	>50	>50	>50	>50	>50	>50	>50	>50	>50	>50
235-47	AG	0.433	0.344	0.239	0.132	>50	2.97	>50	>50	0.700	0.005	0.041	3.11
242-14	AG	>50	1.21	>50	>50	>50	>50	>50	>50	>50	>50	>50	>50
263-8	AG	2.49	1.61	>50	0.484	0.236	>50	>50	>50	4.50	0.610	0.302	>50
269-12	AG	>50	0.802	0.071	0.128	0.019	>50	>50	>50	>50	0.015	0.061	2.74
271-11	AG	>50	>50	>50	>50	>50	>50	5.15	3.08	>50	>50	>50	>50
928-28	AG	>50	2.20	4.03	5.76	>50	>50	>50	>50	>50	0.432	0.601	>50
DJ263.8	AG	0.074	1.55	0.371	0.058	6.04	>50	>50	>50	0.756	0.016	0.085	0.201
T250-4	AG	22.5	10.1	0.095	0.004	0.009	>50	0.181	0.092	19.1	0.004	0.003	>50
T251-18	AG	2.52	0.872	1.75	3.05	7.18	>50	>50	>50	9.96	0.077	0.434	>50
T253-11	AG	5.70	2.66	1.16	>50	5.61	>50	>50	>50	>50	1.10	2.42	>50
T255-34	AG	>50	6.45	>50	2.04	>50	>50	>50	>50	>50	>50	>50	>50
T257-31	AG	3.79	>50	>50	>50	17.4	>50	>50	>50	>50	>50	>50	>50
T266-60	AG	>50	>50	0.818	0.143	0.013	>50	>50	>50	>50	>50	0.233	6.21
T278-50	AG	>50	14.0	>50	>50	0.089	>50	>50	>50	>50	7.33	0.188	>50
T280-5	AG	1.18	2.28	0.111	0.016	0.006	0.071	>50	>50	>50	0.002	0.013	0.044
T33-7	AG	>50	>50	>50	>50	>50	>50	>50	>50	>50	>50	>50	>50
3988.25	B	6.31	0.135	0.060	0.013	0.006	>50	>50	0.864	0.516	0.004	0.015	0.090
5768.04	B	>50	0.145	>50	0.118	0.780	>50	>50	4.48	2.26	>50	1.41	>50
6101.10	B	0.293	6.47	0.032	0.012	0.007	0.146	1.57	0.234	1.72	0.003	0.020	0.124
6535.3	B	1.53	15.1	0.067	0.006	0.013	>50	0.005	0.339	2.57	0.002	0.013	0.102
7165.18	B	2.80	0.042	0.302	0.036	0.003	>50	>50	3.87	0.617	0.005	0.070	0.281
45_01dG5	B	0.112	0.039	0.033	0.011	0.017	0.095	>50	0.225	0.396	0.005	0.019	0.099
89.6.DG	B	>50	>50	0.345	0.034	0.010	>50	>50	3.40	0.397	0.009	0.019	0.267
AC10.29	B	>50	0.356	0.283	0.067	0.008	>50	0.877	3.18	>50	0.002	0.032	1.18
ADA.DG	B	3.03	0.320	0.214	0.033	0.017	13.5	0.811	0.400	10.5	0.009	0.041	0.463
BaL.01	B	>50	3.67	0.576	0.011	0.046	0.303	0.905	0.597	1.74	0.034	0.032	0.176
BaL.26	B	18.4	0.809	0.469	0.023	0.025	0.168	0.039	0.407	0.787	0.003	0.026	0.221
BG1168.01	B	>50	9.37	>50	>50	>50	>50	>50	>50	>50	>50	>50	>50
BL01.DG	B	0.170	>50	>50	19.6	>50	7.91	>50	>50	4.98	0.960	3.41	>50
BR07.DG	B	>50	0.052	>50	0.132	0.705	0.308	>50	>50	2.72	0.016	0.095	>50
BX08.16	B	3.96	0.088	0.110	0.009	0.005	0.018	2.71	0.337	6.55	0.003	0.008	0.074
CAAN.A2	B	>50	0.193	0.254	0.019	0.113	3.26	0.290	>50	>50	0.013	0.016	0.088
CNE10	B	3.93	0.119	0.201	0.019	0.008	3.10	>50	0.152	0.081	0.003	0.015	>50
CNE12	B	1.43	0.097	0.131	0.015	0.020	0.130	0.223	0.351	>50	0.005	0.019	0.128
CNE14	B	0.067	0.072	0.057	0.010	0.014	0.117	0.237	0.129	>50	0.004	0.011	0.066
CNE4	B	3.27	>50	1.88	1.90	1.60	0.215	>50	>50	15.2	>50	0.150	>50
CNE57	B	0.394	39.6	0.087	0.028	0.012	>50	>50	2.60	>50	0.009	0.045	0.135
HO86.8	B	>50	0.205	>50	>50	>50	>50	0.016	>50	>50	>50	>50	>50
HT593.1	B	>50	0.095	>50	>50	>50	6.82	>50	>50	4.50	0.032	0.960	>50
HXB2.DG	B	10.2	0.494	>50	>50	6.83	0.413	>50	>50	0.626	0.005	0.667	>50
JRCSF.JB	B	9.92	1.57	0.119	0.056	0.008	0.237	>50	0.427	0.626	0.005	0.036	1.17
JRFL.JB	B	>50	0.184	0.182	0.040	0.016	>50	5.56	0.212	0.360	0.006	0.026	0.075
MN.3	B	18.8	>50	>50	>50	>50	>50	>50	>50	>50	>50	0.673	>50
PVO.04	B	1.38	2.33	0.812	0.189	0.014	>50	>50	>50	1.41	0.007	0.140	0.447
SIVmac251.30		>50	>50	>50	>50	>50	>50	>50	>50	>50	>50	>50	>50

<u>IC50</u>

Virus ID	Clade	N26-VRC35	N26-VRC36	CH0765-VRC41.01	PGT121	PGT128	PGT135	VRC24	N170-VRC29.03	2G12	BG18	10-1074	DH270
QH0515.01	B	0.410	0.092	8.02	2.83	>50	0.212	>50	>50	0.038	0.053	0.301	>50
QH0692.42	B	1.59	0.645	1.38	0.972	0.040	>50	>50	>50	4.99	0.024	0.198	3.96
REJO.67	B	>50	>50	>50	1.96	>50	>50	0.134	19.2	>50	>50	>50	>50
RHPA.7	B	0.924	>50	0.470	0.036	0.043	>50	>50	>50	>50	3.13	0.058	0.285
SC422.8	B	2.55	0.080	0.869	0.115	0.324	4.38	>50	>50	5.65	0.020	0.095	0.646
SF162.LS	B	4.79	3.31	0.180	0.007	0.003	0.126	0.038	0.069	0.596	0.003	0.008	0.036
SS1196.01	B	0.730	0.633	0.309	0.012	0.014	>50	>50	1.80	13.3	0.004	0.009	0.080
THRO.18	B	>50	>50	>50	>50	>50	>50	>50	>50	>50	>50	>50	>50
TRJO.58	B	12.4	0.223	0.492	1.63	0.018	>50	>50	>50	>50	3.28	0.193	>50
TRO.11	B	0.425	0.030	0.207	0.020	0.013	0.107	0.548	2.13	0.358	0.003	0.034	0.189
WITO.33	B	1.73	0.807	0.616	0.479	>50	>50	>50	>50	0.735	0.094	0.253	>50
X2278.C2.B6	B	0.313	0.084	0.194	0.026	0.006	>50	2.36	0.304	0.084	0.003	0.032	0.855
YU2.DG	B	0.265	17.2	0.666	0.040	0.114	19.7	10.5	1.11	>50	0.040	0.100	0.634
BJOX002000.03.2	BC	>50	0.624	0.079	0.026	0.017	>50	5.59	6.25	>50	0.008	0.012	0.096
CH038.12	BC	3.69	0.199	0.083	0.011	0.005	0.202	12.2	2.69	0.069	0.003	0.020	0.217
CH070.1	BC	10.6	2.12	0.084	0.014	0.032	0.070	15.2	>50	>50	0.003	0.032	0.621
CH117.4	BC	>50	>50	>50	>50	>50	>50	0.276	>50	>50	>50	>50	>50
CH119.10	BC	>50	0.807	0.176	0.031	0.024	>50	>50	8.75	>50	0.012	0.017	0.235
CH181.12	BC	3.05	2.08	0.145	0.031	0.037	0.063	8.19	>50	>50	0.006	0.057	0.773
CNE15	BC	>50	>50	>50	6.65	>50	>50	>50	>50	>50	>50	>50	>50
CNE19	BC	>50	0.890	0.457	0.009	>50	1.56	>50	3.69	>50	>50	0.103	>50
CNE20	BC	11.4	>50	0.029	0.005	0.003	0.008	>50	>50	>50	0.002	0.007	>50
CNE21	BC	3.52	8.10	0.058	0.014	0.011	0.099	>50	>50	>50	0.003	0.049	0.535
CNE40	BC	1.27	17.4	>50	0.281	>50	>50	>50	>50	>50	0.173	0.446	>50
CNE7	BC	>50	>50	7.72	0.048	0.063	>50	>50	>50	>50	>50	0.108	>50
286.36	C	0.642	>50	0.031	0.012	0.013	0.167	2.09	0.539	>50	0.011	0.006	0.119
288.38	C	11.7	0.103	0.158	0.016	0.016	0.057	11.8	0.463	29.0	0.003	0.021	0.148
0013095-2.11	C	0.803	>50	>50	>50	>50	>50	>50	>50	>50	6.03	4.22	>50
001428-2.42	C	>50	0.686	0.084	0.034	0.035	0.106	>50	6.32	>50	0.007	0.061	>50
0077_V1.C16	C	>50	43.8	>50	>50	>50	>50	>50	>50	>50	>50	>50	>50
00836-2.5	C	>50	>50	>50	>50	>50	>50	>50	>50	>50	>50	>50	>50
0921.V2.C14	C	>50	36.3	>50	>50	>50	0.019	>50	>50	>50	>50	>50	>50
16055-2.3	C	>50	10.0	>50	0.312	>50	>50	>50	>50	>50	>50	>50	>50
16845-2.22	C	>50	0.030	2.55	2.72	0.162	9.60	>50	>50	>50	0.019	0.828	>50
16936-2.21	C	>50	1.16	0.082	0.011	0.041	0.071	>50	5.12	>50	0.009	0.008	0.725
25710-2.43	C	1.55	14.8	0.237	0.023	0.021	1.72	>50	>50	>50	0.004	0.060	2.07
25711-2.4	C	3.72	1.39	0.100	0.017	0.026	>50	>50	>50	>50	0.004	0.023	0.200
25925-2.22	C	10.9	0.060	0.143	0.045	0.015	>50	>50	0.020	>50	0.005	0.019	>50
26191-2.48	C	>50	>50	0.067	0.085	0.038	>50	>50	>50	>50	>50	0.072	>50
3168.V4.C10	C	0.205	0.158	15.1	0.446	>50	1.42	>50	>50	>50	0.005	0.531	0.856
3637.V5.C3	C	2.34	>50	>50	>50	43.0	0.746	>50	>50	>50	0.008	>50	>50
3873.V1.C24	C	>50	0.677	0.052	0.045	0.026	0.300	>50	>50	>50	0.005	0.093	0.374
426c	C	>50	0.327	>50	>50	>50	44.2	>50	>50	>50	>50	0.064	>50
6322.V4.C1	C	>50	2.92	>50	>50	>50	>50	>50	>50	>50	>50	>50	>50
6471.V1.C16	C	0.970	6.63	0.419	>50	>50	>50	>50	>50	>50	2.16	0.559	>50
6631.V3.C10	C	5.79	5.17	17.7	>50	1.10	>50	>50	>50	>50	1.43	0.210	>50
6644.V2.C33	C	0.241	1.63	0.099	0.037	0.048	0.047	3.83	17.3	>50	0.004	0.038	0.128
6785.V5.C14	C	0.064	0.235	0.153	0.050	0.016	0.335	0.092	0.384	>50	0.005	0.031	0.247
6838.V1.C35	C	>50	>50	>50	0.061	>50	>50	1.30	>50	>50	>50	>50	>50
96ZM651.02	C	>50	>50	0.140	0.021	0.038	>50	5.67	14.9	>50	>50	0.033	0.660
BR025.9	C	>50	0.175	0.061	0.010	0.005	0.030	0.008	0.217	0.344	0.003	0.007	0.060
CAP210.E8	C	1.15	1.60	9.44	4.28	>50	>50	>50	>50	>50	2.27	>50	>50
CAP244.D3	C	>50	0.461	>50	>50	>50	>50	0.666	>50	>50	>50	>50	>50
CAP256.206.C9	C	7.07	>50	0.130	0.020	0.016	>50	>50	>50	>50	0.012	0.027	0.128
CAP45.G3	C	>50	>50	>50	0.866	>50	>50	>50	>50	>50	>50	>50	>50
Ce1176.A3	C	2.23	0.621	0.141	0.024	0.015	>50	>50	7.00	>50	0.006	0.092	0.294
Ce703010217.B6	C	3.08	>50	0.128	0.007	0.037	>50	>50	>50	>50	>50	0.011	0.457
CNE30	C	5.89	14.5	1.36	0.114	0.342	>50	>50	10.7	>50	0.017	0.363	3.02
CNE31	C	22.0	>50	0.517	0.654	2.04	13.6	>50	>50	>50	>50	0.372	>50
CNE53	C	>50	0.298	0.229	0.026	0.027	0.144	3.76	6.36	>50	0.007	0.029	0.406
CNE58	C	>50	0.120	>50	9.34	1.22	>50	>50	>50	>50	0.015	0.119	2.20
DU123.06	C	>50	>50	0.367	0.048	0.049	>50	>50	>50	>50	0.033	0.098	0.531
DU151.02	C	>50	>50	0.039	0.012	0.010	>50	>50	1.63	>50	0.029	0.014	>50
DU156.12	C	>50	>50	0.082	0.015	0.026	>50	9.28	4.14	>50	0.004	0.027	0.234
DU172.17	C	15.1	33.0	0.197	0.065	0.034	>50	>50	>50	>50	>50	0.100	13.8
DU422.01	C	>50	>50	0.273	0.062	0.070	>50	>50	5.82	>50	0.057	0.088	0.554
MW965.26	C	1.20	>50	0.160	0.018	1.32	3.66	>50	>50	>50	>50	0.014	0.135
SO18.18	C	0.008	0.331	0.336	0.005	0.042	0.060	>50	>50	>50	0.003	0.011	0.073
TV1.29	C	>50	3.74	2.15	0.146	0.053	>50	>50	>50	10.8	0.011	0.664	8.94
TZA125.17	C	6.35	>50	1.06	1.45	0.464	48.2	>50	>50	>50	>50	1.21	>50
TZBD.02	C	10.1	6.00	5.90	0.026	>50	0.052	>50	>50	>50	0.005	0.035	1.82
ZA012.29	C	>50	>50	0.268	0.012	0.030	>50	>50	>50	>50	0.113	0.021	9.33
ZM106.9	C	>50	0.227	0.121	0.013	0.012	0.725	>50	3.84	>50	0.004	0.018	0.127
ZM109.4	C	>50	>50	>50	1.96	>50	>50	>50	>50	>50	>50	>50	>50
ZM135.10a	C	>50	17.6	18.5	0.748	6.63	>50	>50	>50	>50	0.030	0.113	2.12
ZM176.66	C	8.91	7.86	0.276	4.10	0.035	>50	>50	>50	>50	0.095	0.305	>50
ZM197.7	C	>50	>50	>50	>50	>50	>50	>50	>50	>50	>50	>50	>50
ZM214.15	C	>50	>50	0.713	0.392	0.880	>50	>50	>50	>50	>50	0.522	46.9
ZM215.8	C	>50	>50	0.387	0.031	0.048	0.510	>50	>50	>50	1.10	0.038	5.13
ZM233.6	C	1.58	>50	>50	0.731	>50	0.615	0.002	>50	>50	>50	0.112	>50
ZM249.1	C	0.142	>50	6.22	>50	1.66	>50	>50	1.90	>50	>50	>50	>50
ZM53.12	C	>50	1.57	>50	0.003	>50	>50	>50	>50	>50	>50	>50	>50
ZM55.28a	C	>50	>50	1.78	0.081	0.028	>50	49.5	0.825	>50	1.33	0.055	0.145
3326.V4.C3	CD	37.9	>50	>50	>50	>50	>50	>50	>50	>50	>50	>50	>50
3337.V2.C6	CD	1.55	>50	8.19	1.39	0.004	>50	>50	>50	>50	>50	0.057	34.0
3817.v2.c59	CD	0.018	>50	1.99	22.2	0.017	>50	>50	>50	4.21	0.190	0.248	0.736
191821.E6.1	D	0.223	9.50	>50	>50	0.029	>50	0.121	>50	>50	>50	>50	>50
231965.c1	D	2.28	>50	>50	>50	>50	>50	>50	>50	>50	>50	>50	>50
247-23	D	0.677	3.08	>50	>50	>50	>50	>50	>50	>50	>50	>50	>50
3016.v5.c45	D	>50	0.419	>50	>50	>50	>50	>50	>50	>50	>50	>50	>50
57128.vrc15	D	0.376	3.88	0.803	1.27	0.115	>50	>50	>50	1.88	0.368	0.288	17.4
6405.v4.c34	D	>50	0.108	0.734	0.042	2.12	21.2	>50	>50	12.1	0.005	0.045	>50
A03349M1.vrc4a	D	>50	>50	>50	0.075	0.021	>50	>50	>50	>50	>50	0.071	>50
A07412M1.vrc12	D	>50	>50	>50	0.042	15.6	>50	>50	>50	>50	0.182	0.017	>50
NKU3006.ec1	D	16.3	>50	>50	>50	9.99	>50	>50	>50	>50	>50	>50	>50
UG021.16	D	3.38	0.240	>50	0.916	>50	0.369	>50	>50	5.43	0.014	0.039	>50
UG024.2	D	12.6	0.126	>50	>50	>50	0.004	>50	>50	0.162	0.013	1.11	>50
P0402.c2.11	G	>50	>50	0.069	0.012	0.015	>50	>50	>50	>50	0.012	0.014	14.8
P1981.C5.3	G	4.47	>50	0.041	0.007	0.013	>50	1.69	0.177	>50	0.006	0.006	0.047
X1193.c1	G	1.92	0.368	0.198	0.046	0.017	0.114	>50	>50	>50	0.016	0.139	31.3
X1254.c3	G	>50	0.311	0.098	0.035	0.023	2.16	7.20	>50	>50	0.006	0.083	0.259
X1632.S2.B10	G	>50	>50	>50	>50	>50	>50	>50	>50	>50	>50	>50	>50
X2088.c9	G	1.81	7.73	0.272	0.006	14.4	0.066	>50	>50	>50	0.004	0.008	0.158
X2131.C1.B5	G	0.376	0.754	0.094	0.013	0.015	0.106	>50	>50	>50	0.005	0.010	0.972
MuLV		>50	>50	>50	>50	>50	>50	>50	>50	>50	>50	>50	>50

Supplementary Table 3. IC80 values (μg/ml) of HIV-1 glycan-V3-specific bNAbs

IC80

Virus ID	Clade	N26-VRC35	N26-VRC36	CH0765-VRC41.01	PGT121	PGT128	PGT135	VRC24	N170-VRC29.03	2G12	BG18	10-1074	DH270
0260.v5.c36	A	2.88	1.64	1.54	0.159	0.135	19.2	>50	>50	>50	0.014	0.403	5.38
0330.v4.c3	A	20.2	18.9	>50	0.211	3.27	>50	>50	>50	10.4	>50	0.155	>50
0439.v5.c1	A	>50	0.262	>50	>50	7.85	14.7	>50	>50	>50	>50	>50	>50
3365.v2.c20	A	>50	4.71	0.480	0.639	>50	0.319	>50	>50	>50	0.015	0.247	>50
3415.v1.c1	A	8.82	7.65	0.591	>50	0.034	1.53	>50	>50	7.31	0.147	>50	>50
3718.v3.c11	A	17.7	>50	>50	4.76	>50	>50	>50	>50	>50	>50	>50	>50
398-F1_F6_20	A	6.96	9.84	0.104	0.053	0.010	>50	>50	6.48	>50	0.004	0.038	0.101
BB201.B42	A	10.7	2.34	0.132	0.024	0.011	>50	>50	>50	2.77	0.008	0.030	>50
BB539.2B13	A	>50	>50	>50	>50	>50	>50	>50	>50	>50	>50	>50	>50
BG505.W6M.C2	A	>50	>50	>50	0.165	0.569	>50	>50	>50	>50	>50	>50	>50
BI369.9A	A	2.71	0.573	>50	0.085	0.081	0.320	>50	>50	15.7	0.009	0.161	1.01
BS208.B1	A	>50	>50	>50	>50	>50	>50	>50	>50	>50	>50	>50	>50
KER2008.12	A	>50	13.9	>50	6.56	10.3	>50	>50	>50	>50	0.548	0.889	>50
KER2018.11	A	>50	2.84	>50	>50	>50	>50	>50	>50	>50	>50	>50	>50
KNH1209.18	A	6.87	>50	0.222	0.029	0.022	0.206	0.089	0.803	5.99	0.007	0.031	0.205
MB201.A1	A	>50	>50	>50	0.028	0.101	>50	3.09	>50	>50	>50	>50	>50
MB539.2B7	A	>50	9.61	>50	>50	4.76	>50	>50	>50	>50	>50	>50	>50
MI369.A5	A	6.81	1.11	0.889	0.128	0.117	0.478	>50	1.79	35.4	0.012	0.151	1.32
MS208.A1	A	>50	0.587	>50	>50	>50	3.47	>50	>50	>50	>50	>50	>50
Q23.17	A	>50	4.47	0.273	0.027	0.043	>50	2.36	0.197	>50	0.010	0.024	0.138
Q259.17	A	>50	>50	>50	>50	>50	>50	>50	>50	>50	>50	>50	>50
Q769.d22	A	>50	>50	>50	>50	>50	>50	>50	>50	>50	>50	>50	>50
Q769.h5	A	>50	>50	>50	>50	>50	>50	>50	>50	>50	>50	>50	>50
Q842.d12	A	>50	14.2	>50	0.060	0.101	>50	>50	35.7	>50	>50	>50	>50
QH209.14M.A2	A	>50	>50	>50	>50	>50	>50	>50	>50	>50	>50	>50	>50
RW020.2	A	4.81	>50	0.226	0.018	0.011	>50	39.1	0.174	>50	0.005	0.013	0.116
UG037.8	A	>50	>50	4.65	0.307	0.101	>50	>50	>50	>50	0.403	0.379	7.61
246-F3.C10.2	AC	>50	>50	>50	>50	0.012	>50	>50	>50	>50	>50	>50	>50
3301.V1.C24	AC	>50	>50	2.05	0.050	0.734	>50	>50	>50	>50	0.050	0.055	1.50
3589.V1.C4	AC	4.99	3.09	>50	>50	0.055	>50	>50	25.1	11.4	>50	>50	>50
6540.v4.c1	AC	>50	2.41	>50	>50	2.20	>50	>50	>50	>50	>50	>50	>50
6545.V4.C1	AC	>50	12.4	>50	>50	>50	>50	>50	>50	>50	>50	>50	>50
0815.V3.C3	ACD	0.273	5.31	0.902	0.112	0.096	>50	2.78	22.9	>50	>50	0.181	1.64
6095.V1.C10	ACD	>50	0.280	>50	>50	>50	>50	>50	>50	>50	>50	>50	>50
3468.V1.C12	AD	10.1	>50	>50	0.413	>50	>50	>50	>50	>50	0.644	0.031	>50
Q168.a2	AD	>50	>50	>50	>50	>50	>50	>50	>50	>50	>50	>50	>50
Q461.e2	AD	>50	>50	>50	>50	>50	>50	>50	>50	>50	>50	>50	>50
620345.c1	AE	>50	>50	>50	>50	>50	>50	>50	>50	>50	>50	>50	>50
BJOX009000.02.4	AE	>50	0.850	>50	9.02	0.027	>50	>50	>50	>50	>50	>50	>50
BJOX010000.06.2	AE	>50	5.38	>50	>50	10.2	>50	>50	>50	>50	>50	>50	>50
BJOX025000.01.1	AE	17.5	20.5	>50	>50	>50	>50	>50	>50	>50	>50	>50	>50
BJOX028000.10.3	AE	4.62	>50	>50	>50	0.066	>50	>50	>50	29.1	17.8	>50	>50
C1080.c3	AE	>50	>50	>50	>50	0.328	>50	>50	>50	>50	>50	>50	>50
C2101.c1	AE	>50	37.4	>50	>50	0.067	>50	>50	>50	>50	>50	>50	>50
C3347.c11	AE	>50	5.80	>50	>50	0.029	>50	>50	>50	>50	>50	>50	>50
C4118.09	AE	>50	>50	>50	>50	>50	>50	>50	>50	>50	>50	>50	>50
CM244.ec1	AE	>50	>50	>50	>50	0.036	>50	>50	>50	>50	>50	>50	>50
CNE3	AE	>50	1.73	>50	>50	>50	>50	>50	>50	>50	>50	>50	>50
CNE5	AE	>50	>50	>50	>50	0.376	>50	>50	>50	>50	>50	>50	>50
CNE55	AE	>50	1.22	>50	>50	>50	>50	>50	>50	>50	>50	>50	>50
CNE56	AE	>50	1.92	>50	>50	16.7	>50	>50	>50	>50	>50	>50	>50
CNE59	AE	>50	3.28	>50	>50	>50	>50	>50	>50	>50	>50	>50	>50
CNE8	AE	>50	18.3	>50	>50	0.029	>50	>50	>50	>50	>50	>50	>50
M02138	AE	>50	0.477	>50	>50	>50	>50	>50	>50	>50	>50	>50	>50
R1166.c1	AE	>50	>50	>50	>50	>50	>50	>50	>50	>50	>50	>50	>50
R2184.c4	AE	>50	13.6	>50	>50	3.32	>50	>50	>50	>50	>50	>50	>50
R3265.c6	AE	4.77	>50	>50	>50	>50	>50	>50	>50	>50	>50	>50	>50
TH023.6	AE	>50	>50	>50	>50	>50	>50	>50	>50	>50	>50	>50	>50
TH966.8	AE	>50	>50	>50	>50	0.077	>50	>50	>50	>50	>50	>50	>50
TH976.17	AE	>50	>50	>50	>50	>50	>50	>50	>50	>50	>50	>50	>50
235-47	AG	1.25	0.965	1.21	0.614	>50	48.7	>50	>50	1.97	0.016	0.126	12.1
242-14	AG	>50	6.53	>50	>50	>50	>50	>50	>50	>50	>50	>50	>50
263-8	AG	13.3	4.72	>50	2.50	1.07	>50	>50	>50	35.1	3.28	2.18	>50
269-12	AG	>50	1.78	0.188	0.627	0.049	>50	>50	>50	>50	0.037	0.138	9.15
271-11	AG	>50	>50	>50	>50	>50	>50	>50	27.3	>50	>50	>50	>50
928-28	AG	>50	5.89	10.3	29.9	>50	>50	>50	>50	>50	3.06	2.14	>50
DJ263.8	AG	0.269	>50	1.34	0.202	>50	>50	>50	>50	3.70	0.135	0.257	0.865
T250-4	AG	>50	>50	0.321	0.012	0.015	>50	2.14	0.287	>50	0.017	0.007	>50
T251-18	AG	13.3	3.95	5.82	23.3	>50	>50	>50	>50	>50	0.230	2.03	>50
T253-11	AG	>50	15.0	6.65	>50	>50	>50	>50	>50	>50	9.61	>50	>50
T255-34	AG	>50	>50	>50	>50	>50	>50	>50	>50	>50	>50	>50	>50
T257-31	AG	13.4	>50	>50	>50	>50	>50	>50	>50	>50	>50	>50	>50
T266-60	AG	>50	>50	2.08	0.548	0.035	>50	>50	>50	>50	>50	0.881	30.9
T278-50	AG	>50	>50	>50	>50	0.376	>50	>50	>50	>50	>50	1.32	>50
T280-5	AG	5.03	9.87	0.464	0.038	0.022	0.550	>50	>50	>50	0.005	0.032	0.169
T33-7	AG	>50	>50	>50	>50	>50	>50	>50	>50	>50	>50	>50	>50
3988.25	B	>50	0.695	0.139	0.029	0.017	>50	>50	3.25	1.99	0.007	0.031	0.184
5768.04	B	>50	0.368	>50	0.917	3.94	>50	>50	19.8	32.9	>50	>50	>50
6101.10	B	1.43	>50	0.144	0.035	0.022	0.553	>50	0.685	7.35	0.007	0.060	0.327
6535.3	B	5.56	>50	0.160	0.022	0.038	>50	0.024	1.36	31.5	0.006	0.037	0.276
7165.18	B	11.5	0.106	1.04	0.103	0.031	>50	>50	12.1	3.60	0.010	0.165	0.815
45_01dG5	B	0.458	0.093	0.226	0.026	0.060	0.318	>50	0.843	2.05	0.009	0.035	0.301
89.6.DG	B	>50	>50	1.33	0.078	0.025	>50	>50	19.1	9.21	0.019	0.060	0.628
AC10.29	B	>50	1.27	1.19	0.149	0.027	>50	21.5	9.36	>50	0.006	0.112	4.47
ADA.DG	B	12.5	1.72	0.937	0.109	0.071	>50	>50	1.61	>50	0.018	0.099	1.97
Bal.01	B	>50	>50	>50	0.028	24.2	4.01	40.7	1.90	>50	>50	0.090	0.636
BaL.26	B	>50	2.15	2.28	0.060	0.157	0.984	0.995	1.47	12.1	0.032	0.099	0.541
BG1168.01	B	>50	>50	>50	>50	>50	>50	>50	>50	>50	>50	>50	>50
BL01.DG	B	0.691	>50	>50	>50	>50	26.1	>50	>50	27.1	3.68	14.1	>50
BR07.DG	B	>50	0.143	>50	0.596	6.25	0.850	>50	>50	>50	0.072	0.325	>50
BX08.16	B	21.0	0.260	0.379	0.021	0.016	0.051	16.8	0.916	>50	0.005	0.017	0.198
CAAN.A2	B	>50	0.521	0.717	0.050	>50	28.2	>50	>50	>50	0.042	0.043	0.267
CNE10	B	14.5	0.394	0.616	0.048	0.035	>50	>50	0.617	0.309	0.007	0.040	>50
CNE12	B	4.98	0.259	0.461	0.035	0.057	0.366	22.7	1.14	>50	0.011	0.048	0.325
CNE14	B	0.474	0.223	0.214	0.024	0.049	0.404	12.8	0.397	>50	0.010	0.028	0.153
CNE4	B	11.7	>50	39.0	25.3	19.7	1.01	>50	>50	>50	>50	0.597	>50
CNE57	B	1.42	>50	0.322	0.078	0.028	>50	>50	9.33	>50	0.019	0.105	0.291
HO86.8	B	>50	0.480	>50	>50	>50	>50	10.0	>50	>50	>50	>50	>50
HT593.1	B	>50	0.224	>50	>50	>50	>50	>50	>50	37.6	0.120	4.50	>50
HXB2.DG	B	>50	1.39	>50	>50	>50	10.6	>50	>50	1.65	0.012	4.73	>50
JRCSF.JB	B	>50	4.99	0.320	0.162	0.020	0.801	>50	1.31	3.70	0.013	0.109	4.55
JRFL.JB	B	>50	1.05	0.620	0.107	0.048	>50	>50	0.547	2.14	0.013	0.081	0.202
MN.3	B	>50	>50	>50	>50	>50	>50	>50	>50	>50	>50	>50	>50
PVO.04	B	4.16	6.04	2.90	0.498	0.033	>50	>50	>50	8.19	0.018	0.332	1.39
SIVmac251.30		>50	>50	>50	>50	>50	>50	>50	>50	>50	>50	>50	>50

IC80

Virus ID	Clade	N26-VRC35	N26-VRC36	CH0765-VRC41.01	PGT121	PGT128	PGT135	VRC24	N170-VRC29.03	2G12	BG18	10-1074	DH270
QH0515.01	B	1.44	0.285	>50	>50	>50	0.709	>50	>50	0.169	0.435	0.757	>50
QH0692.42	B	11.1	1.23	3.92	5.80	0.135	>50	>50	>50	16.6	0.072	0.674	24.1
REJO.67	B	>50	>50	>50	>50	>50	>50	3.40	>50	>50	>50	>50	>50
RHPA.7	B	3.50	>50	1.44	0.093	0.181	>50	>50	>50	>50	>50	0.157	1.20
SC422.8	B	6.92	0.163	1.90	0.300	1.39	>50	>50	>50	>50	0.163	0.221	1.82
SF162.LS	B	>50	9.40	0.938	0.021	0.015	0.762	0.136	0.206	14.1	0.006	0.016	0.096
SS1196.01	B	2.41	1.99	1.10	0.031	0.036	>50	>50	6.90	>50	0.008	0.024	0.214
THRO.18	B	>50	>50	>50	>50	>50	>50	>50	>50	>50	>50	>50	>50
TRJO.58	B	>50	0.627	1.83	18.6	0.048	>50	>50	>50	>50	>50	0.714	>50
TRO.11	B	1.50	0.075	0.683	0.058	0.052	0.376	15.8	10.3	1.20	0.007	0.091	0.510
WITO.33	B	8.89	2.26	2.00	2.02	>50	>50	>50	>50	4.29	0.324	0.972	>50
X2278.C2.B6	B	0.752	0.348	0.516	0.072	0.023	>50	>50	1.13	0.485	0.006	0.097	4.58
YU2.DG	B	1.58	>50	2.28	0.126	1.63	>50	>50	2.58	>50	>50	0.253	1.81
BJOX002000.03.2	BC	>50	1.43	0.264	0.068	0.053	>50	>50	27.5	>50	0.019	0.035	0.236
CH038.12	BC	18.7	0.603	0.240	0.029	0.013	3.07	>50	10.2	0.243	0.008	0.053	0.674
CH070.1	BC	>50	7.54	0.315	0.042	0.082	0.182	>50	>50	>50	0.010	0.082	1.70
CH117.4	BC	>50	>50	>50	>50	>50	>50	>50	>50	>50	>50	>50	>50
CH119.10	BC	>50	2.47	0.475	0.094	0.067	>50	>50	36.7	>50	0.023	0.058	0.412
CH181.12	BC	27.7	5.86	0.397	0.083	0.112	0.203	>50	>50	>50	0.015	0.164	2.52
CNE15	BC	>50	>50	>50	>50	>50	>50	>50	>50	>50	>50	>50	>50
CNE19	BC	>50	4.41	31.8	0.041	>50	7.79	>50	23.6	>50	>50	>50	>50
CNE20	BC	>50	>50	0.089	0.013	0.008	0.020	>50	>50	>50	0.006	0.019	>50
CNE21	BC	18.4	>50	0.176	0.038	0.027	0.349	>50	>50	>50	0.006	0.110	1.61
CNE40	BC	5.05	>50	>50	1.21	>50	>50	>50	>50	>50	0.962	29.7	>50
CNE7	BC	>50	>50	>50	0.121	0.177	>50	>50	>50	>50	>50	0.302	>50
286.36	C	3.16	>50	0.157	0.027	0.035	1.23	>50	1.73	>50	0.031	0.029	0.342
288.38	C	>50	0.515	0.429	0.044	0.043	0.245	>50	1.36	>50	0.008	0.047	0.390
0013095-2.11	C	3.62	>50	>50	>50	>50	>50	>50	>50	>50	36.8	>50	>50
001428-2.42	C	>50	1.57	0.541	0.108	0.108	0.337	>50	17.0	>50	0.022	0.157	>50
0077_V1.C16	C	>50	>50	>50	>50	>50	>50	>50	>50	>50	>50	>50	>50
00836-2.5	C	>50	>50	>50	>50	>50	>50	>50	>50	>50	>50	>50	>50
0921.V2.C14	C	>50	>50	>50	>50	>50	0.168	>50	>50	>50	>50	>50	>50
16055-2.3	C	>50	19.8	>50	2.78	>50	>50	>50	>50	>50	>50	>50	>50
16845-2.22	C	>50	0.081	8.94	18.6	0.558	>50	>50	>50	>50	0.053	2.07	>50
16936-2.21	C	>50	4.71	0.300	0.024	0.112	0.932	>50	45.2	>50	0.151	0.022	2.05
25710-2.43	C	8.01	>50	0.671	0.066	0.058	>50	>50	>50	>50	0.008	0.160	8.80
25711-2.4	C	21.0	2.33	0.428	0.050	0.070	>50	>50	>50	>50	0.011	0.055	1.50
25925-2.22	C	>50	0.160	0.590	0.087	0.042	>50	>50	0.059	>50	0.011	0.052	>50
26191-2.48	C	>50	>50	0.740	0.236	0.081	>50	>50	>50	>50	>50	0.186	>50
3168.V4.C10	C	1.44	0.371	>50	1.43	>50	7.20	>50	>50	>50	0.014	1.47	4.14
3637.V5.C3	C	15.7	>50	>50	>50	>50	4.93	>50	>50	>50	0.022	>50	>50
3873.V1.C24	C	>50	1.74	0.171	0.113	0.066	1.83	>50	>50	>50	0.012	0.247	0.976
426c	C	>50	1.07	>50	>50	>50	>50	>50	>50	>50	>50	0.173	>50
6322.V4.C1	C	>50	9.50	>50	>50	>50	>50	>50	>50	>50	>50	>50	>50
6471.V1.C16	C	2.55	17.7	1.96	>50	>50	>50	>50	>50	>50	15.2	3.33	>50
6631.V3.C10	C	25.2	14.9	>50	>50	6.10	>50	>50	>50	>50	10.6	0.719	>50
6644.V2.C33	C	1.26	10.2	0.372	0.140	1.60	0.192	>50	>50	>50	0.014	0.100	0.328
6785.V5.C14	C	0.374	0.681	0.514	0.108	0.047	0.499	2.36	1.60	>50	0.012	0.067	0.525
6838.V1.C35	C	>50	>50	>50	0.210	>50	>50	37.5	>50	>50	>50	>50	>50
96ZM651.02	C	>50	>50	0.532	0.061	>50	>50	>50	>50	>50	>50	0.097	>50
BR025.9	C	>50	0.683	0.230	0.025	0.012	0.091	0.038	0.682	2.10	0.006	0.015	0.127
CAP210.E8	C	4.73	3.55	>50	>50	>50	>50	>50	>50	>50	19.5	>50	>50
CAP244.D3	C	>50	1.07	>50	>50	>50	>50	>50	>50	>50	>50	>50	>50
CAP256.206.C9	C	24.9	>50	0.322	0.046	0.045	>50	>50	>50	>50	0.029	0.073	0.325
CAP45.G3	C	>50	>50	>50	>50	>50	>50	>50	>50	>50	>50	>50	>50
Ce1176.A3	C	11.6	2.34	0.386	0.066	0.055	>50	>50	27.5	>50	0.011	0.225	0.755
CE703010217.B6	C	26.8	>50	0.428	0.017	0.137	>50	>50	>50	>50	>50	0.027	1.81
CNE30	C	35.0	>50	4.14	0.296	1.69	>50	>50	>50	>50	0.042	0.887	6.49
CNE31	C	>50	>50	2.23	2.08	10.7	>50	>50	>50	>50	>50	0.972	>50
CNE53	C	>50	0.603	0.641	0.072	0.081	0.570	>50	24.1	>50	0.015	0.075	1.14
CNE58	C	>50	0.347	>50	>50	6.07	>50	>50	>50	>50	0.047	0.419	10.3
DU123.06	C	>50	>50	1.31	0.135	0.174	>50	>50	>50	>50	0.167	0.285	1.72
DU151.02	C	>50	>50	0.148	0.028	0.023	>50	>50	5.93	>50	0.175	0.032	>50
DU156.12	C	>50	>50	0.269	0.037	0.071	>50	>50	15.9	>50	0.011	0.059	0.710
DU172.17	C	>50	>50	0.783	0.410	0.093	>50	>50	>50	>50	>50	0.323	>50
DU422.01	C	>50	>50	0.920	0.176	0.181	>50	>50	14.3	>50	0.390	0.196	1.30
MW965.26	C	2.32	>50	0.528	0.093	38.1	>50	>50	>50	>50	>50	0.046	0.773
SO18.18	C	0.030	1.14	0.908	0.017	0.273	0.173	>50	>50	>50	0.007	0.030	0.167
TV1.29	C	>50	10.8	9.68	0.438	0.193	>50	>50	>50	>50	0.051	2.02	26.1
TZA125.17	C	38.7	>50	3.14	9.43	2.71	>50	>50	>50	>50	>50	7.57	>50
TZBD.02	C	>50	26.9	>50	0.137	>50	0.136	>50	>50	>50	0.023	0.112	10.4
ZA012.29	C	>50	>50	0.870	0.035	0.108	>50	>50	>50	>50	1.49	0.056	>50
ZM106.9	C	>50	0.669	0.386	0.032	0.061	4.11	>50	14.2	>50	0.008	0.041	0.316
ZM109.4	C	>50	>50	>50	>50	>50	>50	>50	>50	>50	>50	>50	>50
ZM135.10a	C	>50	>50	>50	3.69	40.0	>50	>50	>50	>50	0.132	0.385	18.7
ZM176.66	C	>50	>50	0.714	27.6	0.080	>50	>50	>50	>50	0.402	0.929	>50
ZM197.7	C	>50	>50	>50	>50	>50	>50	>50	>50	>50	>50	>50	>50
ZM214.15	C	>50	>50	2.60	1.57	8.07	>50	>50	>50	>50	>50	1.79	>50
ZM215.8	C	>50	>50	1.25	0.084	0.253	>50	>50	>50	>50	>50	0.119	31.1
ZM233.6	C	8.88	>50	>50	3.60	>50	14.1	0.025	>50	>50	>50	0.280	>50
ZM249.1	C	0.512	>50	>50	>50	20.4	>50	>50	9.54	>50	>50	>50	>50
ZM53.12	C	>50	7.15	>50	0.008	>50	>50	>50	>50	>50	>50	>50	>50
ZM55.28a	C	>50	>50	10.7	0.216	0.071	>50	>50	4.24	>50	>50	0.165	0.329
3326.V4.C3	CD	>50	>50	>50	>50	>50	>50	>50	>50	>50	>50	>50	>50
3337.V2.C6	CD	22.5	>50	>50	>50	0.014	>50	>50	>50	>50	>50	0.150	>50
3817.v2.c59	CD	0.049	>50	6.87	>50	0.055	>50	>50	>50	29.7	0.923	1.37	2.76
191821.E6.1	D	0.635	34.7	>50	>50	0.115	>50	5.32	>50	>50	>50	>50	>50
231965.c1	D	7.56	>50	>50	>50	>50	>50	>50	>50	>50	>50	>50	>50
247-23	D	4.47	>50	>50	>50	>50	>50	>50	>50	>50	>50	>50	>50
3016.v5.c45	D	>50	1.26	>50	>50	>50	>50	>50	>50	>50	>50	>50	>50
57128.vrc15	D	1.45	12.3	2.78	>50	1.22	>50	>50	>50	12.3	>50	1.28	>50
6405.v4.c34	D	>50	0.267	6.01	0.136	6.81	>50	>50	>50	>50	0.019	0.119	>50
A03349M1.vrc4a	D	>50	>50	>50	0.359	0.116	>50	>50	>50	>50	>50	0.320	>50
A07412M1.vrc12	D	>50	>50	>50	0.208	>50	>50	>50	>50	>50	4.66	0.038	>50
NKU3006.ec1	D	>50	>50	>50	>50	>50	>50	>50	>50	>50	>50	>50	>50
UG021.16	D	>50	0.679	>50	>50	>50	5.83	>50	>50	>50	0.029	0.280	>50
UG024.2	D	>50	0.240	>50	>50	>50	0.018	>50	>50	0.545	0.046	>50	>50
P0402.c2.11	G	>50	>50	0.255	0.030	0.017	>50	>50	>50	>50	0.036	0.034	>50
P1981.C5.3	G	>50	>50	0.118	0.013	0.073	>50	>50	1.38	>50	0.027	0.017	0.108
X1193.c1	G	13.1	2.51	0.545	0.147	0.050	0.325	>50	>50	>50	0.045	0.331	>50
X1254.c3	G	>50	0.819	0.363	0.106	0.059	26.4	>50	>50	>50	0.014	0.171	0.727
X1632.S2.B10	G	>50	>50	>50	>50	>50	>50	>50	>50	>50	>50	>50	>50
X2088.c9	G	6.59	20.8	0.749	0.019	>50	0.209	>50	>50	>50	0.009	0.016	0.406
X2131.C1.B5	G	1.62	3.69	0.339	0.036	0.052	0.507	>50	>50	>50	0.015	0.024	5.13
MuLV		>50	>50	>50	>50	>50	>50	>50	>50	>50	>50	>50	>50

Supplementary Table 4. Cryo-EM Data Collection, Refinement and Validation Statistics for the VRC35-BG505.T332N DS-SOSIP.664 complex

	**Global map of twelve VRC35 Fabs bound to HIV-1 BG505.T332N DS-SOSIP.664 Env trimer** (EMD-48131)	**Local map of 4 VRC35 Fabs bound to HIV-1 BG505.T332N DS-SOSIP.664 Env trimer** (EMD-48133)	**Local map of 2 VRC35 Fabs bound to HIV-1 BG505.T332N DS-SOSIP.664 Env trimer** (EMD-48134)
**Data collection and processing**			
Magnification	105,000	105,000	105,000
Voltage (kV)	300	300	300
Electron exposure (e^{-}/Å^{2})	54.5	54.5	54.5
Defocus range (μm)	-0.7 to -2.0	-0.7 to -2.0	-0.7 to -2.0
Pixel size (Å)	0.83	0.83	0.83
Symmetry imposed	C3	C1	C1
Final particle images (no.)	299K	66K	66K
Map resolution (Å)	3.39	4.20	4.17
FSC threshold	0.143	0.143	0.143

Supplementary Table 5. Cryo-EM Data Collection, Refinement and Validation Statistics for the VRC35-MEDI8852-H3N2 Victoria 2011 HA complex

	**Composite map of six VRC35 Fabs and three MEDI8852 Fabs bound to influenza H3N2 Victoria 2011 hemagglutinin** (EMD-70607) (PDB-9OM5)	**Global map of six VRC35 Fabs and three MEDI8852 Fabs bound to influenza H3N2 Victoria 2011 hemagglutinin** (EMD-49964)	**Local map of six VRC35 Fabs bound to influenza H3N2 Victoria 2011 hemagglutinin head** (EMD-48426)	**Local map of dimeric VRC35 Fabs bound to *N*-linked glycans N126, N165, and N246 on influenza H3N2 Victoria 2011 hemagglutinin head** (EMD-48427)
**Data collection and processing**				
Magnification	105,000	105,000	105,000	105,000
Voltage (kV)	300	300	300	300
Electron exposure (e^{-}/Å^2)	54.5	54.5	54.5	54.5
Defocus range (μm)	-0.7 to -2.0	-0.7 to -2.0	-0.7 to -2.0	-0.7 to -2.0
Pixel size (Å)	0.83	0.83	0.83	0.83
Symmetry imposed	C3	C3	C3	C1
Final particle images (no.)	n/a	1,780K	1,325K	123K
Map resolution (Å)	3.5	3.39	3.32	3.75
FSC threshold	0.143	0.143	0.143	0.143
**Refinement**				
Initial model used (PDB code)	5JW4			
Map sharpening B factor (Å^2)	n/a	-180.7	-118.2	-131.3
**Model composition**				
Non-hydrogen atoms	42,927			
Protein residues	5424			
B factors (Å^2)(mean)				
Protein	236.33			
R.m.s. deviations				
Bond lengths (Å)	0.024			
Bond angles (°)	0.921			
Validation				
MolProbity score	1.8			
Clash score	6.95			
Poor rotamers (%)	3.52			
Ramachandran plot				
Favored (%)	93.71			
Allowed	6.09			
Disallowed	0.2			

Supplementary Table 6. Cryo-EM Data Collection, Refinement and Validation Statistics for the VRC35-SARS-CoV-2 LP.8.1.9 spike complex

	**Cryo-EM map of VRC35 Fab bound to SARS-CoV-2 LP.8.1.9 spike** (EMD-74798)	**Local density map of VRC35 Fab bound to N-linked glycans on the SARS-CoV-2 LP.8.1.9 spike protein** (EMD-74801)
**Data collection and processing**		
Magnification	105,000	105,000
Voltage (kV)	300	300
Electron exposure (e^{-}/Å^2)	54.2	54.2
Defocus range (μm)	-0.7 to -2.0	-0.7 to -2.0
Pixel size (Å)	0.83	0.83
Symmetry imposed	C1	C1
Final particle images (no.)	55K	56K
Map resolution (Å)	3.68	6.76
FSC threshold	0.143	0.143
**Refinement**		
Map sharpening B factor (Å^2)	-57.4	-672.4

Supplementary Table 7. Cryo-EM Data Collection, Refinement and Validation Statistics for the VRC35-LASV Josiah GPC complex.

	**Cryo-EM map of VRC35 Fab bound to LASV Josiah GPC** (EMD-74843)
**Data collection and processing**	
Magnification	105,000
Voltage (kV)	300
Electron exposure (e^{-}/Å^2)	54.2
Defocus range (μm)	-0.7 to -2.0
Pixel size (Å)	0.83
Symmetry imposed	C1
Final particle images (no.)	62K
Map resolution (Å)	7.24
FSC threshold	0.143
**Refinement**	
Initial model used (PDB code)	
Map sharpening B factor (Å^2)	-648.3

Supplementary Table 8. Cryo-EM Data Collection, Refinement and Validation Statistics for the VRC36-BG505.T332N DS-SOSIP.664 complex

	Composite map of six VRC36 Fabs bound to HIV-1 BG505.T332N DS-SOSIP.664 Env trimer (EMD-49628) (PDB 9NPM)	Global map of six VRC36 Fabs bound to HIV-1 BG505.T332N DS-SOSIP.664 Env trimer (EMD-49633)	Global map of four VRC36 Fabs bound to HIV-1 BG505.T332N DS-SOSIP.664 Env trimer (EMD-47895)	Global map of two VRC36 Fabs bound to HIV-1 BG505.T332N DS-SOSIP.664 Env trimer (EMD-47893)	Local map of dimeric VRC36 Fabs (EMD-47885)
**Data collection and processing**					
Magnification	45,000	45,000	45,000	45,000	45,000
Voltage (kV)	200	200	200	200	200
Electron exposure ($e^{-}/Å^2$)	70.53	70.53	70.53	70.53	70.53
Defocus range (μm)	-0.7 to -2.0	-0.7 to -2.0	-0.7 to -2.0	-0.7 to -2.0	-0.7 to -2.0
Pixel size (Å)	0.88	0.88	0.88	0.88	0.88
Symmetry imposed	C3	C3	C1	C1	C1
Final particle images (no.)	n/a	111K	53K	75K	253K
Map resolution (Å)	3.7	3.59	4.65	5.1	3.62
FSC threshold	0.143	0.143	0.143	0.143	0.143
**Refinement**					
Initial model used (PDB code)	4ZMJ				
Map sharpening B factor ($Å^2$)	n/a	-141.8	-116.9	-246	-159.3
**Model composition**					
Non-hydrogen atoms	35931				
Protein residues	4362				
B factors ($Å^2$)(mean)					
Protein	174.47				
R.m.s. deviations					
Bond lengths (Å)	0.006				
Bond angles (°)	1.188				
Validation					
MolProbity score	1.77				
Clash score	5.86				
Poor rotamers (%)	0.81				
Ramachandran plot					
Favored (%)	92.95				
Allowed	6.86				
Disallowed	0.19				
